# Mono-*N*-alkylation of Amphotericin B and Nystatin A_1_ and Its Amides: Effect on the In Vitro Activity, Cytotoxicity and Permeabilization of Model Membranes

**DOI:** 10.3390/antibiotics13121177

**Published:** 2024-12-04

**Authors:** Olga Omelchuk, Elena Bychkova, Svetlana Efimova, Natalia Grammatikova, George Zatonsky, Lyubov Dezhenkova, Svetlana Solovieva, Olga Ostroumova, Anna Tevyashova, Andrey Shchekotikhin

**Affiliations:** 1 Gause Institute of New Antibiotics, 11 B. Pirogovskaya, Moscow 119021, Russia; 2 Institute of Cytology of Russian Academy of Sciences, 4 Tikhoretsky Ave., St. Petersburg 194064, Russia; 3 School of Science, Constructor University, Campus Ring 1, 28759 Bremen, Germany

**Keywords:** amphotericin B, nystatin, polyene antibiotics, antifungal activity, antibiofilm activity, membrane permeabilization, hemolysis, semi-synthetic derivatives, calcein leakage, large unilamellar vesicles

## Abstract

**Objectives**: In 2022, the World Health Organization highlighted the necessity for the development of new antifungal agents. Polyene antibiotics are characterized by a low risk of drug resistance; however, their use is limited by low solubility and severe side effects. **Methods**: A series of *N*-alkylated derivatives of amphotericin B and nystatin A_1_ as well as their *N*-(2-hydroxyethyl)amides were synthesized. Their antifungal activity was evaluated against various *Candida* strains and *Aspergillus fumigatus* using the broth microdilution method. Cytotoxicity was assessed using an MTT assay on human embryonic kidney cells HEK293 and human skin fibroblast cells hFB-hTERT6, as well as a hemolysis assay on erythrocytes. Membrane activity was analyzed by fluorimetric measurement of calcein leakage from model liposomes. **Results**: Derivatives containing the *N*-(hydroxyethyl)amino)ethyl fragment (compounds **3** and **4**) exhibited relatively high antifungal activity, as did *N*-(2-hydroxyethyl)amides **5** and **9**. Bis-modified compounds **6** and **10** did not outperform their mono-modified analogues in terms of activity or cytotoxicity. The mono-*N*-alkylated compound **3** showed the highest activity/toxicity ratio, which correlated well with its selectivity for ergosterol-containing model membranes. **Discussion**: Combining two successful modifications does not necessarily improve the activity/toxicity ratio of polyenes. Further studies can be performed for the optimization of carboxyl group of **3**.

## 1. Introduction

Nosocomial fungal infections pose a significant threat to patients, particularly those with compromised immune systems [1]. Despite modern advancements in global pharmaceuticals, these infections have a profound impact, with an increasing percentage of treatment failures [2]. In 2022, the World Health Organization (WHO) underscored the urgency of addressing this issue by publishing a list of priority fungal pathogens [3]. This list is considered a critical call to action for the global health community, highlighting the urgent need for intensified research and development efforts in antifungal therapies. The development of new antifungal agents is complicated by the emergence of resistant fungal pathogens, which are becoming increasingly common in clinical settings. The three main classes of antifungal drugs are azoles, echinocandins, and polyene antibiotics. Among these, polyene antibiotics are particularly noteworthy due to their lower propensity for inducing drug resistance compared to azoles and echinocandins [4]. This characteristic makes natural polyenes a promising scaffold for the development of a new generation of antifungal drugs. Despite their potential, polyenes are associated with significant toxicity, especially nephrotoxicity, which has limited their clinical use. Therefore, ongoing research is focused on developing novel polyene derivatives that retain antifungal efficacy while minimizing adverse effects [5,6,7,8,9,10].

**Figure 1:**
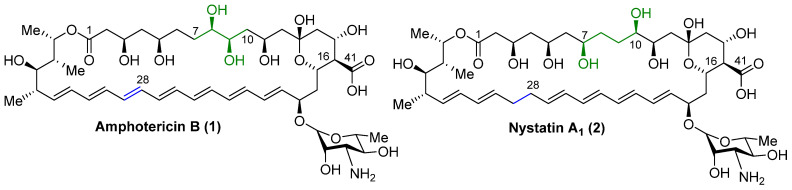
Structures of antifungal polyene antibiotics.

The antifungal effect of polyene antibiotics (amphotericin B (AmB), nystatin A_1_ (Nys), Figure 1) is achieved through their binding to ergosterol, a component specific to fungal cell membranes. This interaction disrupts normal membrane function and leads to fungal cell death [11]. However, the exact mechanism of action of AmB remains a topic of debate [12]. While the formation of transmembrane channels via oligomerization of AmB molecules has been extensively studied [13,14], some studies suggest that this may not be the primary mechanism but rather an enhancer of the antibiotic’s overall effect [15,16,17]. One approach to reducing the toxicity of polyene antibiotics is to increase their selectivity for fungal cells. To achieve this goal, various structural modifications to AmB have been proposed, along with the development of novel dosage forms, both of which have had significant success. For instance, disrupting the hydrogen bonds between the carboxyl group at the C16 position of the aglycone and the amino group in the sugar residue has resulted in an improved pharmacological profile for both AmB and Nys [18,19]. Several amides of these antibiotics [20,21,22,23], carbamide analogues [24], bis-*N*-alkylated derivatives [25,26], and *N*-acylated derivatives of AmB [5,7] are recommended for further in-depth preclinical studies. Additionally, the liposomal formulation of AmB in Ambisome offers significant advantages over the classic drug Fungizone. Its improved safety profile makes amphotericin B the drug of choice for treating severe systemic mycoses, such as mucormycosis, even in pediatric patients [27,28].

Our research group has consistently investigated the structure–activity relationship in polyene antibiotics [9,20,29,30,31]. In this study, we describe a combination of two modification strategies targeting key pharmacophoric positions of AmB and Nys. We also evaluated the antifungal activity, cytotoxicity, and effects of the corresponding derivatives on liposomes designed to mimic fungal and mammalian cell membranes.

## 2. Results

### 2.1. Chemistry

**Scheme 1:**
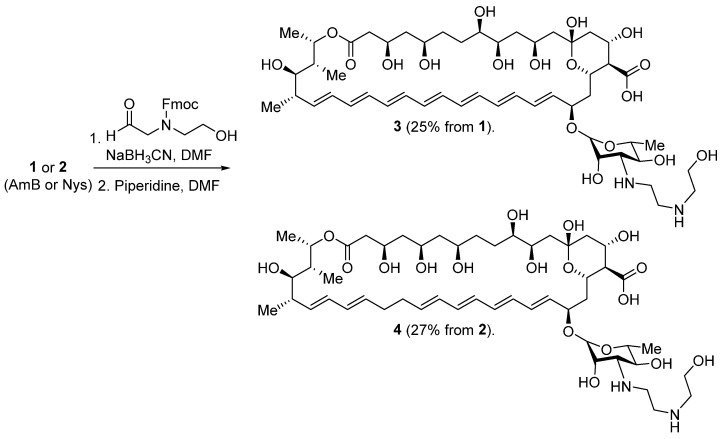
*N*-alkylation of natural polyenes amphotericin B and nystatin A_1_.

We began our research by analyzing the existing literature. Bis-*N*-alkylated derivatives of both natural polyenes and their amides have been previously described [25,26], and it was found that such modifications increased the selectivity of antifungal action. However, the biological properties of polyene derivatives that are mono-*N*-alkylated on the mycosamine moiety have not been extensively studied before. In the first stage of our work, we performed a reductive amination reaction of (9*H*-fluoren-9-yl)methyl (2-hydroxyethyl)(2-oxoethyl)carbamate (synthesized from diethanolamine in two steps, see Supporting Information, p. S59) with the natural polyenes AmB (**1**) and Nys (**2**) using sodium cyanoborohydride in dimethylformamide (DMF). We found that under these conditions, the reaction proceeded slowly and resulted in incomplete conversion of the starting material. The main products were the mono-alkylated derivatives **3** and **4**, while bis-substituted polyenes formed only in trace amounts (Scheme 1). Isolation and purification of both Fmoc-substituted intermediates and final products were challenging due to their low solubility and the difficulty of chromatographic purification using both reverse and normal phase techniques. The most suitable method for preparing derivatives **3** and **4** involved purifying the Fmoc-substituted intermediates by normal phase liquid chromatography, followed by removal of the protective group with piperidine in DMF. This approach allowed us to avoid further chromatographic purification of the final products.

**Scheme 2:**
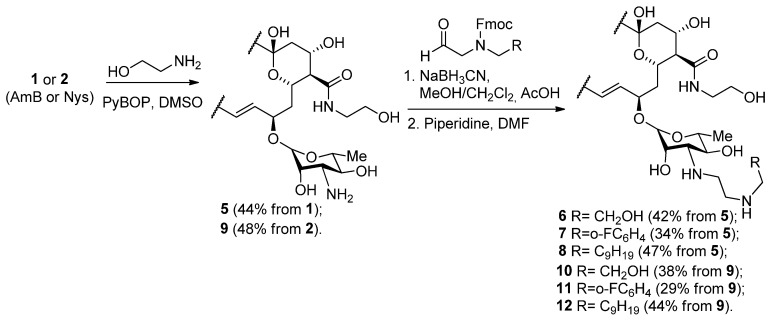
Preparation of 2-hydrohyethylamides of AmB and Nys (**5** and **9**) and their subsequent *N*-alkylation (**6**–**8** and **10**–**12**).

Since these derivatives demonstrated good activity (as discussed in the following sections) but had limited synthetic availability, we pursued a similar modification on the *N*-(2-hydroxyethyl)amides of AmB and Nys as starting materials. It is known that the *N*-(2-hydroxyethyl)amide of Nys exhibits a better activity–toxicity ratio compared to the original antibiotic [21]. The *N*-(2-hydroxyethyl)amide of AmB has also been described, demonstrating slightly higher activity than the parent antibiotic, although no data on its toxicity have been reported [32]. Based on these observations, we decided to synthesize this amide, along with its *N*-alkylated derivatives, for further biological evaluation. Unlike natural polyenes, amides exhibit higher solubility in organic solvents, which allowed us to perform the reductive amination reaction under classical conditions. The *N*-alkylation of the amides of AmB **5** and Nys **9** with NaBH_3_CN in methanol proceeded faster, achieving complete conversion of the starting materials. However, the formation of bis-*N*-akylated products was observed only in trace amounts. To evaluate the effect of different substituents introduced into the mycosamine residue, reactions were also carried out with (9*H*-fluoren-9-yl)methyl 2-fluorobenzyl(2-oxoethyl)carbamate (synthesized from 2-((2-fluorobenzyl)amino)ethanol in two steps, see Supporting Information, p. S59)—compounds **7** and **11**—and (9*H*-fluoren-9-yl)methyldecyl(2-oxoethyl)carbamate (synthesized by method [33])—compounds **8** and **12** (Scheme 2). In these cases, isolation and purification of the target products were performed via normal phase liquid chromatography of the Fmoc-substituted intermediates. The protective group was subsequently removed using piperidine in DMF.

All new derivatives of AmB and Nys **3**–**12** were characterized using HRMS and NMR spectroscopy, including two-dimensional spectra (^1^H-^1^H COSY, TOCSY, HSQC, HMBC, and H2BC). The assignment of signals in the NMR spectra for compounds **3**–**12** is provided in Tables S1 and S2, Supporting Information. Purity was determined by HPLC analysis.

### 2.2. Antifungal Activity

The activity of the obtained derivatives of polyene antibiotics was evaluated against fungal strains listed as critical and high priority by the WHO, including *Aspergillus fumigatus*, *Candida albicans*, *Candida parapsilosis*, *Candida glabrata* and *Candida tropicalis*. Additionally, a clinical isolate strain of *Candida krusei* with natural resistance to fluconazole [34] was tested. The effect of the antibiotics on fungal viability was assessed by determining the minimum inhibitory concentration (MIC) values using the microdilution method in broth with 96-well plates (Table 1).

The results showed that, as expected, *N*-ethanolamides of polyene antibiotics **5** and **9** exhibited superior activity compared to their natural counterparts against all tested fungal strains, which is consistent with previously reported data [21,32]. Mono-*N*-alkylated derivatives containing the *N*-(hydroxyethyl)amino)ethyl fragment in mycosamine residue (compounds **3** and **4**) also demonstrated fairly high antifungal activity. However, contrary to our expectations, combining these two successful modifications into a single structure (compounds **6**, **10**) did not result in a synergistic effect. Other bis-modified derivatives **7**, **8**, **11**, **12** were much less active than the parent antibiotics.

Further biological studies were conducted on the most potent derivatives **3**–**6**, **9**, **10** to evaluate the individual effects of each modification compared to their combined effect.

For AmB derivatives **3**, **5**, **6** antifungal potency against biofilms formed by *Candida* spp. was additionally evaluated. The ability to form biofilms is one of the factors contributing to *Candida* virulence in fungal infections. Clinical data indicate that non-*albicans Candida* isolates form biofilms more frequently than *C. albicans*. Biofilm formation is particularly prevalent among *C. tropicalis* (89.5%) compared to *C. parapsilosis* (41.3%), *C. albicans* (28.6%) and *C. glabrata* (27%) [35]. Additionally, *C. tropicalis* forms aggressive, rapidly developing biofilms consisting of a dense network of yeast cells, pseudohyphae, and hyphae with intensive budding [36]. The study included clinical isolates capable of forming biofilms with high density: *Candida parapsilosis* 58L, *Candida tropicalis* 56-05 and an average density strain of *C. albicans* 604M. For these strains, MIC values for derivatives **3**, **5** and **6** were evaluated using the microdilution method in broth. SMIC_50_ (the concentration resulting in a 50% reduction in the relative metabolic activity of biofilm cells) and SMIC_80_ (the concentration resulting in an 80% reduction) were determined using the MTT assay (Table 2).

The mono-*N*-alkylated derivative of AmB, compound **3,** demonstrated superior antibiofilm activity compared to the original antibiotic, while amide **5** exhibited comparable activity. However, combining these two modifications in compound **6** significantly reduced the antibiofilm activity of the antibiotic.

### 2.3. Evaluation of In Vitro Cytotoxicity

To evaluate the cytotoxicity of the studied polyene antibiotics, we assessed their effects on the viability of human embryonic kidney cells (HEK293) and human skin fibroblast cells hFB-hTERT6 using the MTT assay (Table 3).

As expected, Nys amide **9** demonstrated a significant reduction in in vitro toxicity, consistent with previously published data [21]. The mono-*N*-alkylated derivative of *N*-(2-hydroxyethyl)amide of Nys **10** was also found to be slightly less toxic than the corresponding derivative of the natural antibiotic **4** but more toxic than *N*-(2-hydroxyethyl)amide of Nys **9**. For the AmB derivatives, all tested compounds suppressed cell activity with comparable efficacy; however, the bis-modified derivative exhibited the highest cytotoxicity.

Additionally, the cytotoxicity of AmB derivatives **3**, **5** and **6** was assessed using a hemolysis test. The HR_50_ value, defined as the concentration at which erythrocyte hemolysis reaches 50%, was determined for AmB and its derivatives **3**, **5** and **6** (Table 4).

Unexpectedly, among all the tested derivatives, the mono-*N*-alkylated derivative of AmB **3** showed the lowest cytotoxicity toward erythrocytes, while amide **5** exhibited the highest hemolytic activity.

### 2.4. Fluorimetric Studies on Membrane Models

The effect of the tested polyene antibiotics on the permeability of model membranes at concentrations close to their MIC (5 µM) was investigated by measuring calcein leakage from liposomes. Large unilamellar vesicles were prepared from two lipid compositions: 1-palmitoyl-2-oleoyl-sn-glycero-3-phosphocholine (POPC)/cholesterol (CHOL) (67/33 mol.%), which models mammalian cell membranes, and POPC/ergosterol (ERG) (67/33 mol.%), which models fungal cell membranes. For a first approximation, the kinetics of dye leakage from liposomes induced by the tested polyenes was described using a two-exponential model. The characteristic parameters of this model, maximum leakage (*RF_max_*) and the times associated with the fast and slow components (*t*_1_ and *t*_2_, respectively), are presented in Table 5. The efficacy of the tested AmB derivatives (**3**, **5** and **6**) in releasing the fluorescent marker from POPC/CHOL and POPC/ERG vesicles was higher than that of the paternal antibiotic. In contrast, Nys derivatives exhibited a lower capacity to release calcein from lipid vesicles, with the exception of *N*-(2-hydrohyethyl)amide of Nys **9**, which demonstrated enhanced activity on POPC/ERG liposomes (Table 5).

Interestingly, regardless of the chemical modifications introduced or the composition of the target membrane, all AmB and Nys derivatives demonstrated a tendency to exhibit faster calcein leakage compared to their parent molecules, as evidenced by lower *t*_1_ and *t*_2_ values (Table 5). Since the *t*_1_ value reflects molecular sorption onto membranes and the *t_2_* value corresponds to pore formation, the observed reduction in these parameters compared to the initial molecules is likely due to the faster formation of polyene–sterol complexes. The efficacy of the tested AmB derivatives (**3**, **5** and **6**) to disengage the fluorescence marker from POPC/CHOL and POPC/ERG vesicles (*RF_max_*) was higher than of the paternal antibiotic, and mono-*N*-alkylated derivative **3** showed the best result in selectivity to ergosterol-containing membranes (Table 5). The Nys derivatives showed reduced ability to release calcein from lipid vesicles of both compositions, except for 2-hydroxyethyl amide **9** in POPC/ERG liposomes, which exhibited higher selectivity for ergosterol-containing membranes. These results correlate well with data on in vitro activity and cytotoxicity.

## 3. Discussion

The development of new polyene antibiotics based on AmB and Nys, or their analogues, has been a focus of research for various scientific groups and pharmaceutical companies [8,37,38]. Over the past decades, significant progress has been made in improving the properties of these unique antifungal agents. Several semi-synthetic and genetically engineered derivatives of polyene antibiotics have been proposed as candidates for next-generation drugs [5,18,20,21]. However, to the best of our knowledge, none of these candidates, except for BSG005 (ClinicalTrials.gov ID NCT04921254), have advanced to clinical trials. Although the polyene antibiotic BSG005 has been known for over 15 years [39], human trials only began in 2021. This delay can likely be attributed to insufficient funding for antifungal drug development and limited investor interest, as the shortage in the existing arsenal to combat fungal infections has only recently gained widespread recognition. Consequently, there is now a resurgence—a revival of interest in developing effective antifungal agents. Therefore, it is more relevant than ever to analyze the existing structure–activity relationships among polyene antibiotics and pursue the rational design of new derivatives based on these insights.

Previously, E. Carreira and his scientific group noted the positive effects of bis-*N*-alkylation of the mycosamine residue in polyene antibiotics on their biological properties [26]. They identified the 3-aminopropyl fragment as the most effective substitute in this context. In one study [25], a wide range (30+) of *N*-alkylated derivatives of AmB were described, and only one was mono-*N*-alkylated. In that study, a reductive amination reaction was initially performed, followed by the synthesis of several amides and a methyl ester from the most promising bis-*N*-alkylated derivative. The authors found that all amides obtained were less toxic (based on hemolysis tests) than AmB and their analogues with a free carboxyl group.

Based on these findings, we decided to investigate the biological properties of *N*-alkylated polyene derivatives containing an additional *N*-substituted amino group—modifications that had not been studied by E. Carreira’s research group. Starting with the reductive amination of AmB using (9H-fluoren-9-yl)methyl(2-hydroxyethyl)(2-oxoethyl) carbamate, we found that the reactivity of this aldehyde allowed us to obtain only mono-alkylation products under conditions similar to those described in [25]. Nevertheless, the derivatives **3** and **4** exhibited high antifungal activity.

Since modification of the carboxyl group is known to positively affect the properties of polyene antibiotics [21,23,29], we decided to continue studying mono-*N*-alkylated derivatives using synthetically accessible amides obtained in one step by condensing AmB and Nys with ethanolamine. This approach yielded amide derivatives containing an *N*-(2-aminoethyl)amino fragment in the mycosamine residue, further *N′*-substituted with a 2-hydroxyethyl group (compounds **6**, **10**), a 2-fluorobenzyl fragment (compounds **7**, **11**), and a decyl alkyl chain (compounds **8**, **12**).

The highest antifungal activity was observed for *N*-(2-hydroxyethyl)aminoethyl derivatives **3**, **4**, **6** and **10**. Conversely, the introduction of aromatic or lipophilic alkyl chains negatively affected activity. Additionally, combining two successful modifications did not result in a synergistic effect, contrary to expectations based on the existing literature. In the case of AmB, modification of the carboxyl group led to decreased antifungal potential in compound **6** and increased cytotoxicity (compared to compound **3**). Unexpectedly, *N*-(2-hydroxyethyl)amide of AmB **5** proved to be even more toxic than the original AmB.

Studies on model membranes have shown that biologically active derivatives generally retain membrane activity comparable to that of the original antibiotics [9,20,30,31]. For the mono-*N*-alkylated derivatives studied, the ratio of their membrane activity between the ERG-enriched membrane model (representing fungal membranes and predictive of therapeutic efficacy) and the CHOL-containing membrane model (representing mammalian membranes and indicative of toxicity) correlated well with their in vitro activity and cytotoxicity. However, for the amides AmB and Nys, such a clear correlation has not been observed [31]. Derivative **3** stands out as a lead compound, warranting further investigation to optimize the substituent on the carboxyl group to improve solubility while preserving its desirable biological properties.

## 4. Materials and Methods

### 4.1. Chemistry

AmB, Nys, ethanolamine, diethanolamine, and Dess–Martin periodinane were purchased from abcr GmbH (Karlsruhe, Germany). Sodium cyanoborohydride, benzotriazol-1-yl-oxytripyrrolidinophosphonium hexafluorophosphate (PyBOP), (2-fluorobenzyl)aminoethanol, *N*-(9H-fluoren-9-yl methoxycarbonyloxy)succinimide (Fmoc-OSu), piperidine (Pip), acetonitrile (MeCN), methanol (MeOH), dichloromethane (CH_2_Cl_2_), dimethylsulfoxide (DMSO), dimethyl formamide (DMF), and diethyl ester were purchased from Sigma-Aldrich^®^ (Merck KGaA, Darmstadt, Germany). Water was distilled and deionized using a RiOs-DI^®^ 3 UV Water Purification System (Merck Millipore KGaA, Darmstadt, Germany). Thin-layer chromatography (TLC) analysis was conducted on 0.20 mm silica gel 60 F_254_ plates (aluminum sheets 20 × 20 cm) Macherey-nagel (Duren, Germany) using *n*-PrOH–EtOAc–NH_4_OH, 3:3:2. Flash chromatography was performed using an Isolera Prime purification system (Biotage^®^ SNAP, Stockholm, Sweden) with C18 silica gel cartridges (Biotage^®^ SNAP, Stockholm, Sweden) and SepaFlash columns packed with UltraPure silica gel (Santai Science Inc., Montreal, QC, Canada). High-performance liquid chromatography (HPLC) was carried out on a HPLC instrument LC 20AD series (Shimadzu Corp., Kyoto, Japan). Samples (0.01–0.02 mg/mL, 20 μL) were injected into a Kromasil-100 C18 column (4.6 × 250 mm, particle size 5 μm, Akzo Nobel, Bohus, Sweden) and eluted at a flow rate of 1.0 mL/min using a gradient of 0.2% HCOONH_4_ buffer (pH 4.5) and acetonitrile (30–70%) over 30 min. Detection wavelengths were set at 408 nm for AmB derivatives and 320 nm for Nys derivatives. Bruker Avance III 500 MHz NMR spectrometer (BrukerDaltonics GmbH, Bremen, Germany) was used to record the NMR spectra. Resonance frequencies for ^1^H and ^13^C were 500 MHz and 125 MHz, respectively. Samples were dissolved in DMSO-d6 (residual solvents signals: 2.50 ppm for ^1^H and 39.50 ppm for ^13^C) and spectra were recorded at 303 K with mixing times ranging from 10 to 180 ms. High-resolution mass spectrometry (HRMS ESI) was performed using a Bruker microTOF-Q II™ instrument (BrukerDaltonics GmbH, Bremen, Germany). Unless otherwise stated, chemical modifications of polyenes were carried out under an argon flow and protected from light.

#### 4.1.1. General Procedure for Synthesis of Mono-*N*-alkylated Polyene Derivatives

AmB or Nys (0.216 mmol) and *N*-Fmoc aldehyde (0.650 mmol) were added to 4 mL of DMF, and the mixture was stirred at room temperature for 30 min. Then, NaBH_3_CN (0.041 g, 0.650 mmol) and AcOH (0.05 mL) were added (TLC reaction control system I). The reaction mixture was stirred 24 h, diluted with diethyl ether (50 mL), and the precipitate was filtered, washed with diethyl ether, and dried. The crude product was purified by normal phase liquid column chromatography on silica gel with elution system (CHCl_3_:MeOH:H_2_O:AcOH 5:1:0.05:0.02). Fractions containing the pure compound were combined, evaporated and dried. The resulting product was dissolved in a solution of piperidine (0.6 mL) in DMF (3 mL) and stirred at room temperature. After 2 h, the reaction mixture was diluted with diethyl ether (200 mL), and the precipitate was filtered, washed with diethyl ether, and dried under vacuum for 24 h.

*N*-(2-((2-Hydroxyethyl)amino)ethyl)amphotericin B (**3**) Yield 54 mg (25%), orange powder; Rt 11.21 min; purity 93%; HRMS (ESI) *m*/*z*: [M + H]^+^ Calc. for C_51_H_82_N_2_O_18_^+^ 1011.5635, Found 1011.5624.

*N*-(2-((2-Hydroxyethyl)amino)ethyl)nystatin (**4**) Yield 59 mg (27%); beige powder; Rt 10.97 min; purity 91%; HRMS (ESI) *m*/*z*: [M + H]^+^ Calc. for C_51_H_82_N_2_O_18_^+^ 1013.5792, Found 1013.5785.

#### 4.1.2. General Procedure for Synthesis of *N*-(2-Hydroxyethyl)amides of AmB and Nys

AmB or Nys (0.54 mmol) was dissolved in DMSO (7.0 mL). Then, PyBOP (562.0 mg, 1.08 mmol) and ethanolamine (164.9 mg, 2.7 mmol) were added. The reaction mixture was stirred for 4 h, and then Et_2_O (50 mL) was added. The reaction mixture was shaken vigorously, and the Et_2_O layer was decanted; this procedure was repeated until thick viscous oil was formed. Then, methanol (10 mL) was added, and the mixture was stirred until dissolution; then, Et_2_O (100 mL) was added, and the formed precipitate was filtered off, washed with Et_2_O and dried. The crude product was added to Biotage Sfar C18 (30 g) cartridges and eluted with (A) H_2_O then 0.5% aq. AcOH and (B) acetonitrile (0–30%, 6 CV; 30–55%, 12 CV; 50–100%, 3 CV). Fractions with pure product were combined and evaporated. The residue was dissolved in MeOH with a drop of AcOH; then, Et_2_O (150 mL) was added, and the precipitate of an acetate of a targeted compound was filtered off, washed twice with Et_2_O, and dried under vacuum for 24 h.

*N*-(2-Hydroxyethyl)amide of AmB (**5**): yield: 190 mg (44%); orange powder; Rf 0.49; Rt 15.37 min; purity 96%; HRMS (ESI) *m*/*z*: [M + H]^+^ Calc. for C_49_H_79_N_2_O_17_^+^ 967.5373, Found 967.5405.

*N*-(2-Hydroxyethyl)amide of Nys (**9**): yield: 200 mg (48%); beidge powder; Rf 0.51; Rt 15.51 min; purity 93%; HRMS (ESI) *m*/*z*: [M + H]^+^ Calc. for C_54_H_82_N_3_O_16_^+^ 969.5530, Found 969.5562.

#### 4.1.3. General Procedure for Synthesis of Mono-*N*′-Alkylated *N*-(2-Hydroxyethyl)amides of AmB and Nys

*N*-(2-hydroxyethyl)amide of AmB **5** or Nys **9** (0.20 mmol) was partly dissolved in MeOH (3.0 mL), and solution of the corresponding 2-substituted (9*H*-fluoren-9-yl)methyl (3-oxopropyl)carbamate (0.40 mmol) in dichloromethane (1 mL) was added. The reaction mixture was stirred for 1 h, and then NaBH_3_CN (38.0 mg, 0.60 mmol) was added, followed by the addition of AcOH (0.1 mL). The reaction mixture was stirred for another 24 h. After the reaction was complete, the solvent was removed using a rotary evaporator. The crude product was purified by normal phase liquid column chromatography on silica gel with elution system (CHCl_3_:MeOH:H_2_O:AcOH 5:1:0.5:0.02). Fractions containing the pure compound were combined, evaporated, and dried. The residue was dissolved in a 20% solution of piperidine in DMF (3 mL) and stirred for 2.5 h, followed by addition of Et_2_O (50 mL). The formed precipitate was filtered off and dried.

*N*-(2-Hydroxyethyl)amide of *N′*-(2-((2-hydroxyethyl)amino)ethyl)AmB (**6**): yield: 87 mg (42%); orange powder; Rf 0.45; Rt 15.17 min; purity 91%; HRMS (ESI) *m*/*z*: [M + H]^+^ Calc. for C_53_H_88_N_3_O_18_^+^ 1054.6057, Found 1054.6041.

*N*-(2-Hydroxyethyl)amide of *N′*-(2-((2-fluorobenzyl)amino)ethyl)AmB (**7**): yield: 75 mg (34%); orange powder; Rf 0.52; Rt 14.85 min; purity 96%; HRMS (ESI) *m*/*z*: [M + H]^+^ Calc. for C_54_H_82_N_3_O_16_^+^ 1118.6171, Found 1118.6190.

*N*-(2-Hydroxyethyl)amide of *N′*-(2-(decylamino)ethyl)AmB (**8**): yield: 105 mg (47%); orange powder; Rf 0.54; Rt 23.40 min; purity 98%; HRMS (ESI) *m*/*z*: [M + H]^+^ Calc. for C_54_H_82_N_3_O_16_^+^ 1150.7360, Found 1150.7343.

(2-Hydroxyethyl)amide of *N′*-((2-((2-hydroxyethyl)amino)ethyl)Nys (**10**): yield: 82 mg (38%); beidge powder; Rf 0.47; Rt 16.32 min; purity 93%; HRMS (ESI) *m*/*z*: [M + H]^+^ Calc. for C_54_H_82_N_3_O_16_^+^ 1056.6214, Found 1056.6264.

*N*-(2-Hydroxyethyl)amide of *N′*-(2-((2-fluorobenzyl)amino)ethyl)Nys (**11**): yield: 68 mg (29%); beidge powder; Rf 0.55; Rt 14.47 min; purity 92%; HRMS (ESI) *m*/*z*: [M + H]^+^ Calc. for C_54_H_82_N_3_O_16_^+^ 1120.6327, Found 1120.6377.

*N*-(2-Hydroxyethyl)amide of *N′*-(2-(decylamino)ethyl)Nys (**12**): yield: 104 mg (44%); beidge powder; Rf 0.54; Rt 22.66 min; purity 94%; HRMS (ESI) *m*/*z*: [M + H]^+^ Calc. for C_54_H_82_N_3_O_16_^+^ 1152.7517, Found 1152.7515.

### 4.2. Antifungal Activity Testing

The method for determining antifungal activity was previously described in earlier works [9,31] and was used unchanged in this study. Briefly, fungi strains of *Candida* spp. and *Aspergillus fumigatus* ATCC 46645 were originated from the State Scientific Center for Antibiotics (Moscow, Russia). The antifungal activity of polyenes was assessed using the EUCAST broth microdilution method, following the E.DEF 7.3.2 and E.DEF 9.3.2 protocols. The study was conducted in sterile, disposable 96-well flat-bottom plates with a capacity of 300 µL per well (Corning Inc., Corning, NY, USA). PRMI 1640 medium supplemented with 2% (*m*/*v*) glucose, L-glutamine and without bicarbonate (Merck KGaA, Darmstadt, Germany) was used as the liquid nutrient medium. After incubation for 24 h for *Candida* spp. and 48–72 h for *A. fumigatus*, the minimum inhibitory concentration (MIC) was determined as the lowest concentration at which complete inhibition of microbial growth was observed.

### 4.3. Cell Culture and Antiproliferative Activity

The method for determining antifungal activity was previously described in earlier works [9,31] and was used unchanged in this study. Briefly, Dulbecco’s modified Eagle’s medium (PanEco, Moscow, Russia) supplemented with 10% fetal calf serum (HyClone, Logan, UT, USA), 2 mM L-glutamine, 100 U/mL penicillin, and 100 mg/mL streptomycin was used for cultivation of cell lines HEK293 and hFB-hTERT6. An MTT cell proliferation assay was used to determine the cytotoxic effect of tested samples. The IC_50_ (50% growth inhibitory concentration) was defined as the concentration of the compound that inhibited MTT conversion by 50%.

### 4.4. Hemolysis Assay

The studies were carried out according to the method described by Sæbø et al. [40]. Samples were dissolved in DMSO to a concentration of 10,000 µg/mL, and then diluted in phosphate-buffered saline (PBS) to concentrations ranging from 40 µg/mL to 1.25 µg/mL. Fresh human donor blood, collected no more than 24 h before analysis, was centrifuged at 2000 rpm for 5 min. The supernatant was removed, and PBS was added to restore the original volume. Washing was repeated until the supernatant was clear. Red blood cells were diluted to a 1% suspension in PBS. A mixture of 0.5 mL erythrocyte suspension and 0.5 mL sample solution was added to the tubes. A 4% Triton X-100 solution served as a positive control, while PBS was used as a negative control. The prepared sample tubes were incubated at 37 °C for 60 min. After incubation, the samples were centrifuged at 3000 rpm for 5 min. Then, 100 µL of each solution was transferred to the wells of the plates. Optical density measurements were performed at a wavelength of 405 nm, and the hemolysis ratio (HR) was determined using the following Equation (1), taking into account the positive and negative controls:

(1) $$\mathrm{HR}\left(\%\right)=\frac{\mathrm{ODtest}-\mathrm{ODneg}}{\mathrm{ODpos}-\mathrm{ODneg}}\times 100\%,$$

where ODtest—test compound, ODneg—negative control (PBS), ODpos—positive control (100% lysis). The concentration causing 50% hemolysis (HR_50_) was calculated graphically using dose–response values.

### 4.5. Antibiofilm Activity

Clinical strains with high-density biofilm formation included in the study were *C. parapsilosis* 58L, *C. tropicalis* 56-05, and medium-density *C. albicans* 604M. The biofilms were formed in 96-well plates using RPMI-MOPS medium (SIGMA, St. Louis, MO, USA) as previously described [9]. An inoculum was prepared at a concentration of 10^6^ CFU/mL, added to the 100 µL wells of the plate, and incubated for 2 h at 36 °C. After this period, unattached cells were removed by washing twice with 200 µL of sterile physiological solution per well. Biofilms were then formed in RPMI-MOPS medium at 36 °C for 24–48 h, depending on the study objectives. After 24 h of incubation, the planktonic cells were removed, washed twice with 200 µL of saline solution, and 100 µL of fresh medium was added.

Biofilm morphology at different growth stages was assessed using a light microscope (IMF-020 with a digital camera and Capture 2.4 software (Lacopa, Moscow, Russia)). The tested drugs were added to the biofilms at concentrations ranging from 64 to 0.5 µg/mL and incubated for 24 h at 36 °C. Efficacy was evaluated using an MTT assay. For this purpose, the liquid phase was removed, and an MTT solution (50 µL, 1 mg/mL) was added to each well. The plates were incubated for 2 h at 36 °C, and then the liquid was removed followed by the addition of DMSO (100 µL) to each well. To ensure complete dissolution of the formazan, the plates were incubated in the dark at room temperature for an additional 2 h. Optical density (OD) at 580 nm was measured using a FlexA-200HT flatbed spectrophotometer with Readerlt-I software (V1.0.2 221020) (Hangzhou Allsheng Instruments Co., Hangzhou, China). The OD values of control biofilms without exposure were set as 100%. Logistic regression models were used for dose–effect approximation in Microsoft Excel; with polynomials of 2–3 degrees, the R^2^ reliability was between 0.96 and 0.98. Concentrations for determining the minimum inhibitory concentration (SMIC) of 50% and 80% were derived from the graphical data. Each experiment was performed in 4–6 replicates and repeated independently 2–3 times.

### 4.6. Calcein Release from Unilamellar Vesicles

All chemicals were purchased from Avanti Research, Inc. (Alabaster, AL, USA) and Sigma-Aldrich Company Ltd. (Gillingham, UK). Briefly, liposomes were prepared from POPC/Chol (67/33 mol%) and POPC/Erg (67/33 mol%) by extrusion using Avanti Research (Alabaster, AL, USA), and tested compounds were added to calcein-loaded liposomes from stock solution (10 mM in DMSO) in a final concentration of 5 μM. The degree of calcein release was detected at 25 °C using a “Fluorat-02-Panorama” spectrofluorimeter (Lumex, Saint-Petersburg, Russia). A time-dependent increase in fluorescence of released calcein was measured over 30−60 min. Triton X-100 (1%) was added as a positive control at the end of each experiment (for completion of calcein release and detection of maximal fluorescence).

The membrane activity of the tested polyenes was evaluated in relation to the intensity of calcein fluorescence in liposomes (*RF*, %), using the following Formula (2):

(2) $$\mathrm{RF}=\frac{I-I_{0}}{I_{\max}/0.9-I_{0}}\cdot 100\%,$$

where *I*—the calcein fluorescence intensity in the sample treated with polyene, *I*_0_—the calcein fluorescence intensity in the untreated sample (negative control), and *I*_max_—the maximal calcein fluorescence intensity in the sample treated with Triton X-100 (positive control). In order to calculate the dilution of the sample by Triton X-100, the factor of 0.9 was introduced.

Time series data of calcein release were modelled using two-exponential functions with characteristics times *t*_1_ and *t*_2_ corresponding to the fast and slow components of calcein release, respectively.

The values of *RF_max_*, *t*_1_ and *t*_2_ were reported as mean ± standard error (s.e.), with statistical significance set at *p* ≤ 0.05.

## Figures and Tables

**Table 1:** Minimum inhibitory concentrations (MICs, µg/mL) of AmB (**1**), Nys (**2**) and its derivatives **3**, **5**–**12** against *Candida* spp. strains and *Aspergillus fumigatus*.

MIC, µg/mL												
Tested Strains	Compound											
	1	3	5	6	7	8	2	4	9	10	11	12
*C. parapsilosis* ATCC 22019	1	0.5	0.06	0.125	4	4	4	2	0.5	1	8	4
*C. parapsilosis* 58L	2	1	2	2	4	4	4	4	1	2	16	8
*C. albicans* ATCC 10231	1	0.25	0.25	0.25	2	2	2	1	1	1	4	4
*C. albicans* 8R	1	0.5	0.5	1	2	2	2	2	1	0.5	8	4
*C. albicans* 604M	0.5	0.5	0.5	1	2	4	2	1	2	1	8	4
*C. krusei* 432M	2	1	1	2	2	4	4	4	2	4	8	4
*C. tropicalis* 3010	0.5	0.25	0.125	0.25	2	4	2	1	0.5	1	8	4
*C. glabrata* 61L	1	0.5	1	1	2	2	2	1	1	1	4	4
*A. fumigatus* ATCC 46645	2	1	1	1	2	2	4	2	1	2	8	4

**Table 3:** Cytotoxicity of AmB (**1**), Nys (**2**) and its derivatives **3**–**6**, **9**, **10** against human embryonic kidney cells (HEK293) and human skin fibroblast cells hFB-hTERT6.

IC_50_, μM		
	HEK293	hFB-hTERT6
AmB (**1**)	16.5 ± 4.2	2.1 ± 0.3
**3**	19.6 ± 0.4	3.9 ± 0.2
**5**	14.1 ± 0.5	3.5 ± 0.4
**6**	9.2 ± 0.2	1.4 ± 0.2
Nys (**2**)	29.1 ± 3.7	40.3 ± 5.8
**4**	55.6 ± 7.7	63.4 ± 8.2
**9**	110.5 ± 14.3	220.5 ± 30.8
**10**	48.1 ± 5.8	95.0 ± 11.4

**Table 2:** Antibiofilm activity of AmB (**1**) and its derivatives **3**, **5**, **6**.

	MIC, μg/mL	SMIC_50_, μg/mL	SMIC_80_, μg/mL
*C. albicans* 604M			
AmB (**1**)	1	1.46 (±0.08)	7.46 (±1.10)
**3**	0.5/1	1.25 (±0.12)	5.11 (±0.45)
**5**	1	1.58 (±0.05)	8.55 (±0.95)
**6**	1	1.99 (±0.17)	11.23 (±0.46)
*C. tropicalis* 56-05			
AmB (**1**)	2	3.54 (±0.63)	7.16 (±0.84)
**3**	1/2	2.20 (±0.05)	5.34 (±0.48)
**5**	1	3.83 (±0.54)	8.46 (±1.20)
**6**	1	7.32 (±0.78)	15.31 (±0.94)
*C. parapsilosis* 58L			
AmB (**1**)	2	1.71 (±0.72)	6.67 (±0.73)
**3**	1/2	1.15 (±0.08)	3.70 (±0.18)
**5**	2	2.57 (±0.54)	8.52 (±0.58)
**6**	2	3.81 (±0.84)	12.33 (±0.42)

**Table 4:** The hemolytic activity of amphotericin B and its derivatives **3**, **5** and **6**.

	HR_50_, μg/mL
AmB (**1**)	10.05 ± 1.91
**3**	23.71 ± 3.15
**5**	2.14 ± 0.16
**6**	3.01 ± 0.34

**Table 5:** The characteristic parameters of the dependence of calcein release from unilamellar vesicles of indicated composition induced by 5 μM of various polyenes on time.

	POPC/CHOL (67/33 mol.%)			POPC/ERG (67/33 mol.%)			*RF_ERG_*/*RF_CHOL_*
Polyene	*RF_max_*, % ^†^	*t*_1_, min ^††^	*t*_2_, min ^††^	*RF_max_*, % ^†^	*t*_1_, min ^††^	*t*_2_, min ^††^	
AmB (**1**)	6 ± 1	0.6 ± 0.3	12.4 ± 8.1	9 ± 1	0.3 ± 0.1	5.8 ± 1.1	1.5 ± 0.4
**3**	14 ± 2 *	0.8 ± 0.2	7.4 ± 0.4	25 ± 2 *	0.1 ± 0.1	1.8 ± 0.3 *	1.8 ± 0.4
**5**	16 ± 3 *	0.2 ± 0.1	2.7 ± 0.3 *	24 ± 3 *	0.2 ± 0.1	1.7 ± 0.2 *	1.5 ± 0.5
**6**	19 ± 4 *	0.1 ± 0.1 *	0.5 ± 0.2 *	33 ± 2 *	0.2 ± 0.1	1.6 ± 0.3 *	1.7 ± 0.5
Nys (**2**)	10 ± 2	0.9 ± 0.3	5.7 ± 1.9	19 ± 3	0.7 ± 0.1	5.4 ± 0.5	1.9 ± 0.1
**4**	4 ± 2 *	0.5 ± 0.2	2.4 ± 0.2 *	14 ± 2	0.2 ± 0.1 *	2.6 ± 0.5 *	2.3 ± 1.1
**9**	5 ± 1 *	0.6 ± 0.2	2.5 ± 0.7 *	22 ± 3	0.5 ± 0.2	2.4 ± 0.4 *	3.7 ± 1.7
**10**	7 ± 2	0.4 ± 0.1 *	3.0 ± 0.3 *	12 ± 2 *	0.3 ± 0.1 *	4.3 ± 1.0	2.0 ± 1.0

^†^ *RF_CHOL_*, *RF_ERG_*—maximal polyene-induced leakage of calcein from unilamellar vesicles composed of POPC/CHOL (67/33 mol.%) and POPC/ERG (67/33 mol.%), respectively; ^††^ *t*_1_, *t*_2_—characteristic times referring to fast and slow components, respectively, of two-exponential dependence fitting the time dependence of calcein release. * The difference between the values of parent AmB or Nys and their derivatives was assessed by one-way ANOVA followed by Tukey HSD tests and was statistically significant for compounds marked with an asterisk (*p* < 0.05).

## Data Availability

Data are contained within the article and Supplementary Materials.

## Supplementary Materials

The following supporting information can be downloaded at: https://www.mdpi.com/article/10.3390/antibiotics13121177/s1, Table S1: ^1^H and ^13^C spectra assignment for amphotericin B derivatives **3**, **5**–**8**; Table S2: ^1^H and ^13^C spectra assignment for nystatin A_1_ derivatives **4**, **9**–**12**; Figures S1–S4. NMR spectra of the AmB derivative **3**; Figures S5–S8: NMR spectra of the Nys derivative **4**; Figures S9–S12: NMR spectra of the AmB derivative **5**; Figures S13–S16: NMR spectra of the AmB derivative **6**; Figures S17–S21: NMR spectra of the AmB derivative **7**; Figures S22–S25: NMR spectra of the AmB derivative **8**; Figures S26–S29: NMR spectra of the Nys derivative **9**; Figures S30–S33: NMR spectra of the Nys derivative **10**; Figures S34–S38: NMR spectra of the Nys derivative **11**; Figures S39–S42: NMR spectra of the Nys derivative **12**; Figures S43–S52: HPLC chromatograms of compounds **3**–**12**; Page S59: synthesis of 2-substituted (9H-fluoren-9-yl)methyl (3-oxopropyl)carbamates.
