# Supplementary material for: Mono-N-alkylation of Amphotericin B and Nystatin A1 and Its Amides: Effect on the In Vitro Activity, Cytotoxicity and Permeabilization of Model Membranes
Source: Antibiotics (Basel). 2024 Dec 4;13(12):1177. doi: 10.3390/antibiotics13121177 (PMC11672593; doi:10.3390/antibiotics13121177)
Supplement: Supplementary file 1 [file antibiotics-13-01177-s001.zip › antibiotics-3342705-supplementary.pdf]

SUPPORTING INFORMATION

Mono-*N*-alkylation of amphotericin B and nystatin A1 and its amides: effect on the *in vitro* activity, toxicity and permeabilization of model membranes

Olga Omelchuk [1,*], Elena Bychkova [1], Svetlana Efimova [2], Natalia Grammatikova [1], George Zatonsky [1], Lyubov Dezhenkova [1], Svetlana Solovieva [1], Olga Ostroumova [2], Anna Tevyashova [1,3] and Andrey Shchekotikhin [1]

[1] Gause Institute of New Antibiotics, 11 B. Pirogovskaya, Moscow, 119021, Russia;
[2] Institute of Cytology of the Russian Academy of Sciences, 4 Tikhoretsky ave., St. Petersburg, 194064, Russia
[3] School of Science, Constructor University, Campus Ring 1, 28759 Bremen, Germany;
* Correspondence: omelchuk.93@mail.ru

TABLE OF CONTENTS

Table S1. ^{1}H and ^{13}C spectra assignment for AmB derivatives **3**, **5-8** 2

Table S2. ^{1}H and ^{13}C spectra assignment for Nys derivatives **4**, **9-12** 7

Figures S1-S4. NMR spectra of the AmB derivative **3**. 12

Figure S5-S8. NMR spectra of the Nys derivative **4**. 16

Figure S9-S12. NMR spectra of the AmB derivative **5**. 20

Figure S13-S16. NMR spectra of the AmB derivative **6**. 24

Figure S17-S21. NMR spectra of the AmB derivative **7**. 28

Figure S22-S25. NMR spectra of the AmB derivative **8**. 33

Figure S26-S29. NMR spectra of the Nys derivative **9**. 37

Figure S30-S33. NMR spectra of the Nys derivative **10**. 41

Figure S34-S38. NMR spectra of the Nys derivative **11**. 45

Figure S39-S42. NMR spectra of the Nys derivative **12**. 50

Figure S43-S52. HPLC chromatograms of the derivatives **3-12**. 54

Synthesis of 2-substituted (9H-fluoren-9-yl)methyl (3-oxopropyl)carbamates .. 59

Table S1. ^{1}H and ^{13}C spectra assignment for amphotericin derivatives **3**, **5-8**

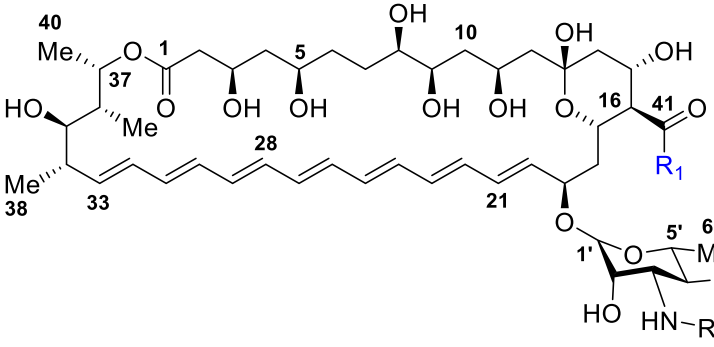

R_1=OH, NHC_2H_4OH
R_2=H, $C_2H_4NHC_2H_4OH$, $C_2H_4NHCH_2(o\text{-}FC_6H_4)$, $C_2H_4NHC_9H_{19}$

Compound. ^{13}C/^{1}*H* δ. ppm (*J*, Hz)					
**Atom**	**3**	**5**	**6**	**7**	**8**
**Aglycone**					
**1 O-C(O)**	170.5	170.5	170.5	170.5	170.4
	-	-	-	-	-
**2 CH₂**	42.0	42.0	42.0	42.0	41.9
	2.17	*2.17*	*2.16*	*2.17*	*2.31*
**3 CH**	66.2	66.2	66.2	66.1	66.4
	4.07	*4.06*	*4.05*	*4.06*	*4.07*
**4 CH₂**	44.7	44.7	44.7	44.7	44.5
	1.34; 1.40	*1.33; 1.40*	*1.33; 1.40*	*1.33; 1.40*	*1.42; 1.40*
**5 CH**	69.2	69.1	69.1	69.1	69.4
	3.54	*3.53*	*3.53*	*3.53*	*3.61*
**6 CH₂**	35.0	35.0	35.0	35.0	34.8
	1.40; 1.28	*1.40; 1.29*	*1.40; 1.28*	*1.40; 1.28*	*1.40; 1.38*
**7 CH₂**	29.0	29.0	29.0	29.0	29.0
	1.59; 1.26	*1.57; 1.27*	*1.58; 1.27*	*1.58; 1.27*	*1.55; 1.38*
**8 CH**	73.5	73.6	73.5	73.5	73.7

	3.11	*3.11*	*3.11*	*3.11*	*3.17*
**9 CH**	73.8<br>*3.47*	73.7<br>*3.47*	73.8<br>*3.47*	73.8<br>*3.47*	73.6<br>*3.51*
**10 CH_2**	39,5<br>*1.56; 1.33*	39.6<br>*1.55; 1.33*	39.6<br>*1.56; 1.34*	39.5<br>*1.56; 1.34*	39.9<br>*1.55; 1.39*
**11 CH**	67,7<br>*4.25*	67.7<br>*4.24*	67.7<br>*4.24*	67.7<br>*4.25*	67.5<br>*4.26*
**12 CH_2**	46,3<br>*1.53*	46.3<br>*1.54*	46.2<br>*1.53*	46.2<br>*1.54*	46.6<br>*1.57*
**13 C-O**	97,0<br>-	97.0<br>-	97.0<br>-	97.0<br>-	97.0<br>-
**14 CH_2**	44.3<br>*1.84;1.08*	44.6<br>*1.87; 1.08*	44.5<br>*1.87; 1.08*	44.5<br>*1.87; 1.08*	44.6<br>*1.91; 1.12*
**15 CH**	65.5<br>*4.01*	64.6<br>*4.04*	64.6<br>*4.04*	64.6<br>*4.04*	64.8<br>*4.06*
**16 CH**	58.1<br>*1.82*	57.0<br>*1.90*	57.0<br>*1.91*	57.0<br>*1.91*	57.2<br>*1.93*
**17 HC-O**	65.4<br>*4.23*	65.2<br>*4.21*	65.1<br>*4.23*	65.1<br>*4.24*	65.4<br>*4.27*
**18 CH_2**	38.0<br>*2.19; 1.51*	36.3<br>*1.98; 1.44*	36.3<br>*2.00; 1.42*	36.4<br>*2.01; 1.44*	36.8<br>*1.99; 1.50*
**19 CH-O**	76.4<br>*4.31*	74.3<br>*4.36*	74.6<br>*4.34*	74.6<br>*4.35*	74.9<br>*4.37*
**20 CH**	136.9<br>*5.98*	136.9<br>*5.95*	136.7<br>*5.96*	136.6<br>*5.96*	137.0<br>*5.99*
**21 CH**	128.4<br>*6.10*	128.5<br>*6.09*	128.5<br>*6.10*	128.5<br>*6.10*	128.5<br>*6.12*

**22 CH**	133.4<br>*6.39*	133.5<br>*6.39*	133.4<br>*6.39*	133.4<br>*6.39*	133.6<br>*6.39*
**23 CH**	131.7<br>*6.27*	131.7<br>*6.25*	131.8<br>*6.25*	131.8<br>*6.26*	131.5<br>*6.25*
**24 CH**	133.8<br>*6.46*	133.9<br>*6.45*	133.8<br>*6.45*	133.8<br>*6.45*	133.8<br>*6.45*
**25 CH**	132.4<br>*6.35*	132.3<br>*6.32*	132.4<br>*6.32*	132.4<br>*6.32*	132.1<br>*6.32*
**26 CH**	133.1<br>*6.32*	133.1<br>*6.32*	133.1<br>*6.31*	133.1<br>*6.31*	133.0<br>*6.31*
**27 CH**	132,4<br>*6.30*	132.1<br>*6.31*	132.1<br>*6.32*	132.2<br>*6.30*	131.8<br>*6.31*
**28 CH**	133,6<br>*6.35*	133.7<br>*6.33*	133.7<br>*6.34*	133.6<br>*6.34*	133.6<br>*6.34*
**29 CH**	132,4<br>*6.31*	132.4<br>*6.29*	132.4<br>*6.29*	132.5<br>*6.29*	132.2<br>*6.30*
**30 CH**	131,8<br>*6.17*	131.8<br>*6.17*	131.8<br>*6.17*	131.8<br>*6.17*	131.6<br>*6.18*
**31 CH**	132.1<br>*6.17*	132.1<br>*6.17*	132.2<br>*6.17*	132.1<br>*6.17*	132.1<br>*6.16*
**32 CH**	131.2<br>*6.09*	131.2<br>*6.09*	131.2<br>*6.09*	131.2<br>*6.08*	130.9<br>*6.10*
**33 CH**	136.7<br>*5.45*	136.7<br>*5.44*	136.7<br>*5.44*	136.7<br>*5.44*	136.8<br>*5.49*
**34 CH**	42.4<br>*2.29*	42.3<br>*2.29*	42.4<br>*2.28*	42.4<br>*2.29*	42.0<br>*2.31*

**35 CH**	77.1<br>*3.09*	77.0<br>*3.08*	77.2<br>*3.09*	77.1<br>*3.09*	77.1<br>*3.13*
**36 CH**	39.6<br>*1.73*	39.6<br>*1.72*	39.6<br>*1.72*	39.6<br>*1.73*	39.9<br>*1.79*
**37 CH**	68.8<br>*5.22*	68.9<br>*5.20*	68.8<br>*5.21*	68.8<br>*5.22*	69.3<br>*5.19*
**38 CH_3**	18.4<br>*1.04*	18.4<br>*1.04*	18.4<br>*1.04*	18.4<br>*1.04*	18.3<br>*1.06*
**39 CH_3**	12.0<br>*0.91*	12.0<br>*0.91*	12.0<br>*0.91*	12.0<br>*0.92*	11.9<br>*0.94*
**40 CH_3**	16.9<br>*1.11*	16.9<br>*1.11*	16.9<br>*1.11*	16.9<br>*1.11*	16.9<br>*1.14*
**41 C=O**	172.1<br>-	172.6<br>-	172.8<br>-	172.0<br>-	171.8<br>-
**Mycosamine**					
**1′ CH**	98.4<br>*4.39*	96.9<br>*4.30*	97.0<br>*4.27*	96.8<br>*4.29*	97.5<br>*4.34*
**2′ CH**	66.1<br>*3.77*	69.7<br>*3.59*	66.2<br>*3.74*	65.9<br>*3.79*	66.8<br>*3.76*
**3′ CH**	61.7<br>*2.43*	56.5<br>*2.38*	62.7<br>*2.27*	62.5<br>*2.37*	62.8<br>*2.30*
**4′ CH**	68.2<br>*3.54*	73.0<br>*2.90*	70.8<br>*2.94*	70.3<br>*3.00*	71.0<br>*2.99*
**5′ CH**	73.1<br>*3.17*	73.1<br>*3.03*	73.1<br>*3.09*	73.0<br>*3.11*	73.1<br>*3.12*
**6′ CH_3**	18.2<br>*1.15*	17.9<br>*1.14*	18.1<br>*1.14*	18.0<br>*1.14*	18.0<br>*1.17*

**8′ CH2**	47.1<br>*2.91; 2.85*	-<br>-	44.4<br>*2.83; 2.59*	44.8<br>*2.86; 2.65*	44.2<br>*2.86; 2.66*
**9′ CH2**	49.7<br>*2.86*	-<br>-	48.2<br>*2.73*	47.6<br>*2.68*	48.3<br>*2.77*
**10′ CH2**	42.7<br>*2.85*	-<br>-	50.6<br>*2.69*	45.4<br>*3.77*	48.2<br>*2.67*
**11′**	57.5<br><br>*3.60*	-<br><br>-	59.2<br><br>*3.51*	126.9<br>J_{C-F} = 15.0 Hz<br>-	29.0<br><br>*1.38*
**12′**	-<br><br>-	-<br><br>-	-<br><br>-	160.3<br>J_{C-F} = 244.0 Hz<br>-	26.4<br><br>*1.29*
**13′**	-<br><br>-	-<br><br>-	-<br><br>-	114.9<br>J_{C-F} = 21.6 Hz<br>*7.15*, t, J = 8.0 Hz	28.8<br><br>*1.28*
**14′**	-<br>-	-<br>-	-<br>-	128.5<br>*7.29*, q, J = 6.9 Hz	28.7<br>*1.29*
**15′**	-<br>-	-<br>-	-<br>-	124.2<br>*7.17*, t, J = 7.3 Hz	28.8<br>*1.27*
**16′**	-<br>-	-<br>-	-<br>-	130.4<br>*7.48*, t, J = 7.3 Hz	28.5<br>*1.27*
**17′**	-<br>-	-<br>-	-<br>-	^{19}F: -119.2 ppm<br>-	31.2<br>*1.27*
**18′**	-<br>-	-<br>-	-<br>-	-<br>-	21.9<br>*1.28*
**19′**	-<br>-	-<br>-	-<br>-	-<br>-	13.7<br>*0.87*
**Amide moiety**					
**NH**	-	*7.92*	*7.92*	*7.91*	*7.63*

**1′′**	-	41.4	41.4	41.4	41.6
	-	*3.22; 3.06*	*3.24; 3.06*	*3.25; 3.06*	*3.24; 3.12*
**2′′**	-	60.0	60.0	60.0	60.2
	-	*3.41*	*3.41*	*3.42*	*3.39*

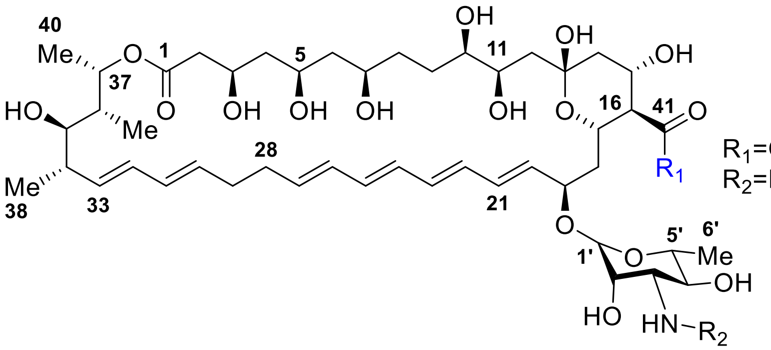

Table S2. ^{1}H and ^{13}C spectra assignment for nystatin derivatives **4***,* **9-12**

Compound. ^{13}C/^{1}*H* δ. ppm (*J*, Hz)					
**Atom**	**4**	**9**	**10**	**11**	**12**
**Aglycone**					
**1 O-C(O)**	170.4	170.3	170.4	170.4	170.4
	-	-	-	-	-
**2 CH_2**	42.5	42.6	42.6	42.6	42.6
	2.34; 2.28	*2.35; 2.29*	*2.34; 2.30*	*2.33; 2.30*	*2.34; 2.29*
**3 CH**	65.7	65.8	65.8	65.9	65.7
	4.02	*4.02*	*4.02*	*4.01*	*4.01*
**4 CH_2**	44.3	44.2	44.2	44.3	44.3
	1.48	*1.49*	*1.49*	*1.51*	*1.49*
**5 CH**	68.0	67.9	68.0	68.2	67.9
	3.80	*3.80*	*3.81*	*3.84*	*3.80*

**6 CH2**	34.4<br>*1.45; 1.38*	34.4<br>*1.47; 1.37*	34.4<br>*1.45; 1.37*	34.2<br>*1.48; 1.40*	34.4<br>*1.45; 1.36*
**7 CH**	69.4<br>*3.60*	69.4<br>*3.60*	69.4<br>*3.60*	69.5<br>*3.62*	69.4<br>*3.59*
**8 CH2**	42.3<br>*1.68; 1.53*	42.1<br>*1.68; 1.56*	42.1<br>*1.66; 1.56*	42.5<br>*1.68; 1.58*	42.1<br>*1.67; 1.55*
**9 CH2**	28.5<br>*1.43*	28.4<br>*1.44*	28.4<br>*1.44*	28.6<br>*1.46*	28.4<br>*1.44*
**10 CH**	73.4<br>*3.22*	73.3<br>*3.25*	73.3<br>*3.24*	73.4<br>*3.26*	73.3<br>*3.24*
**11 CH**	70.2<br>*3.89*	70.0<br>*3.91*	7.1<br>*3.91*	70.1<br>*3.90*	70.1<br>*3.92*
**12 CH2**	44.4<br>*1.45; 1.40*	44.4<br>*1.48; 1.40*	44.4<br>*1.47; 1.39*	44.4<br>*1.48; 1.44*	44.3<br>*1.46; 1.39*
**13 C-O**	97.1<br>-	97.0<br>-	97.0<br>-	97.0<br>-	97.0<br>*1.88; 1.13*
**14 CH2**	44.3<br>*1.88; 1.15*	44.5<br>*1.91; 1.15*	44.6<br>*1.91; 1.14*	44.6<br>*1.91; 1.17*	44.7<br>*4.01*
**15 CH**	65.6<br>*3.97*	64.6<br>*4.01*	64.6<br>*4.01*	64.7<br>*4.03*	64.6<br>*1.91*
**16 CH**	58.0<br>*1.87*	57.2<br>*1.93*	57.3<br>*1.92*	57.3<br>*1.92*	57.3<br>-
**17 HC-O**	66.0<br>*4.01*	65.8<br>*3.93*	65.8<br>*3.95*	65.9<br>*3.99*	65.6<br>*3.97*
**18 CH2**	38.1<br>*1.86; 1.70*	37.3<br>*1.67; 1.61*	37.4<br>*1.64*	37.5<br>*1.66*	37.3<br>*1.64; 1.61*
**19 CH-O**	76.0<br>*4.30*	75.6<br>*4.29*	75.7<br>*4.30*	75.8<br>*4.32*	76.0<br>*4.29*
**20 CH**	134.8<br>*5.77*	134.1<br>*5.66*	134.1<br>*5.67*	134.1<br>*5.67*	134.1<br>*5.65*

**21 CH**	129.9<br>*6.17*	130.8<br>*6.20*	130.6<br>*6.17*	130.5<br>*6.18*	130.7<br>*6.18*
**22 CH**	132.2<br>*6.24*	132.8<br>*6.20*	132.9<br>*6.20*	132.3<br>*6.22*	132.9<br>*6.20*
**23 CH**	131.1<br>*6.20*	131.9<br>*6.24*	132.0<br>*6.23*	132.0<br>*6.22*	132.0<br>*6.24*
**24 CH**	132.2<br>*6.22*	130.6<br>*6.21*	130.6<br>*6.22*	130.5<br>*6.19*	132.5<br>*6.24*
**25 CH**	132.7<br>*6.20*	132.9<br>*6.21*	132.9<br>*6.20*	132.7<br>*6.20*	132.0<br>*6.24*
**26 CH**	131.0<br>*6.12*	131.0<br>*6.12*	131.0<br>*6.12*	131.0<br>*6.10*	130.9<br>*6.12*
**27 CH**	134.1<br>*5.68*	134.2<br>*5.68*	134.2<br>*5.68*	134.4<br>*5.69*	134.2<br>*5.66*
**28 CH_2**	31.6<br>*2.17; 2.10*	31.6<br>*2.17*	31.6<br>*2.18*	31.6<br>*2.19*	31.7<br>*2.15*
**29 CH_2**	31.8<br>*2.17*	31.6<br>*2.17*	31.6<br>*2.18*	31.6<br>*2.19*	31.7<br>*2.15*
**30 CH**	131.2<br>*5.51*	131.1<br>*5.51*	131.1<br>*5.51*	131.1<br>*5.51*	131.2<br>*5.50*
**31 CH**	131.0<br>*5.96*	131.2<br>*5.96*	131.1<br>*5.96*	131.0<br>*5.94*	131.2<br>*5.96*
**32 CH**	129.5<br>*5.96*	129.3<br>*5.97*	129.3<br>*5.96*	129.3<br>*5.96*	129.3<br>*5.98*
**33 CH**	135.3<br>*5.49*	135.5<br>*5.53*	135.4<br>*5.52*	135.7<br>*5.50*	135.6<br>*5.52*
**34 CH**	40.5<br>*2.24*	40.3<br>*2.25*	40.3<br>*2.25*	40.3<br>*2.26*	40.3<br>*2.25*
**35 CH_3**	17.0<br>*0.97*	16.6<br>*0.96*	16.6<br>*0.96*	16.6<br>*0.96*	16.6<br>*0.96*

**36 CH**	76.0<br>*3.15*	75.9<br>*3.14*	75.9<br>*3.14*	76.0<br>*3.17*	75.9<br>*3.14*
**37 CH**	40.0<br>*1.80*	39.9<br>*1.81*	39.9<br>*1.80*	40.0<br>*1.82*	39.9<br>*1.80*
**38 CH_3**	12.0<br>*0.86*	12.0<br>*0.86*	12.0<br>*0.86*	11.9<br>*0.87*	12.0<br>*0.86*
**39 CH**	70.3<br>*5.07*	70.4<br>*5.08*	70.4<br>*5.08*	70.5<br>*5.08*	70.5<br>*5.08*
**40 CH_3**	16.6<br>*1.10*	16.4<br>*1.10*	16.5<br>*1.09*	16.5<br>*1.10*	16.4<br>*1.09*
**41 C=O**	175.9<br>-	171.8<br>-	171.8<br>-	171.8<br>-	171.8<br>-
**Mycosamine**					
**1′ CH**	98.8<br>*4.41*	98.5<br>*4.35*	98.5<br>*4.33*	98.7<br>*4.34*	98.5<br>*4.32*
**2′ CH**	69.4<br>*3.60*	69.9<br>*3.58*	66.6<br>*3.69*	69.5<br>*3.62*	66.5<br>*3.68*
**3′ CH**	62.1<br>*2.39*	56.5<br>*2.40*	62.3<br>*2.29*	62.8<br>*2.28*	62.7<br>*2.26*
**4′ CH**	70.9<br>*2.99*	72.9<br>*2.90*	70.8<br>*2.93*	70.9<br>*2.94*	70.8<br>*2.91*
**5′ CH**	73.0<br>*3.13*	73.0<br>*3.03*	73.0<br>*3.08*	73.0<br>*3.09*	73.1<br>*3.07*
**6′ CH_3**	18.2<br>*1.14*	17.9<br>*1.12*	18.1<br>*1.12*	18.1<br>*1.12*	18.1<br>*1.11*
**7′ CH_2**	43.1<br>*2.84; 2.75*	-<br>-	44.8<br>*2.77; 2.56*	45.5<br>*2.77; 2.56*	45.0<br>*2.75; 2.52*
**8′ CH_2**	47.4<br>*2.83*	-<br>-	48.6<br>*2.66*	45.7<br>*3.75*	48.9<br>*2.61*

**9′ CH₂**	50.0<br>*2.81*	-<br>-	50.9<br>*2.64*	48.7<br>*2.59*	48.8<br>*2.52*
**10′**	58.0<br><br>*3.57*	-<br><br>-	59.5<br><br>*3.48*	127.5<br>J_{C-F} = 15.0 Hz<br>-	28.9<br><br>*1.40*
**11′**	-<br><br>-	-<br><br>-	-<br><br>-	160.3<br>J_{C-F} = 243.2 Hz<br>-	26.7<br><br>*1.25*
**12′**	-<br><br>-	-<br><br>-	-<br><br>-	114.8<br>J_{C-F} = 21.2 Hz<br>*7.11* t, *J* = 9.4 Hz	29.0<br><br>*1.24*
**13′**	-<br>-	-<br>-	-<br>-	128.3<br>*7.26* q, *J* = 7.0 Hz	28.9<br>*1.24*
**14′**	-<br>-	-<br>-	-<br>-	124.1<br>*7.15* t, *J* = 7.0 Hz	28.9<br>*1.24*
**15′**	-<br>-	-<br>-	-<br>-	130.2<br>*7.47* t, *J* = 7.0 Hz	28.7<br>*1.24*
**16′**	-<br>-	-<br>-	-<br>-	^{19}F: -119.5 ppm<br>-	31.2<br>*1.24*
**17′**	-<br>-	-<br>-	-<br>-	-<br>-	22.0<br>*1.24*
**18′**	-<br>-	-<br>-	-<br>-	-<br>-	14.0<br>*0.86*
**Amide moiety**					
**NH**	-	*8.81*	*7.88*	*7.86*	*7.88*
**1′′**	-<br>-	41.4<br>*3.19; 3.08*	41.4<br>*3.19; 3.07*	41.4<br>*3.19; 3.11*	41.4<br>*3.18; 3.07*
**2′′**	-<br>-	59.9<br>*3.39*	60.0<br>*3.40*	60.0<br>*3.40*	60.0<br>*3.39*

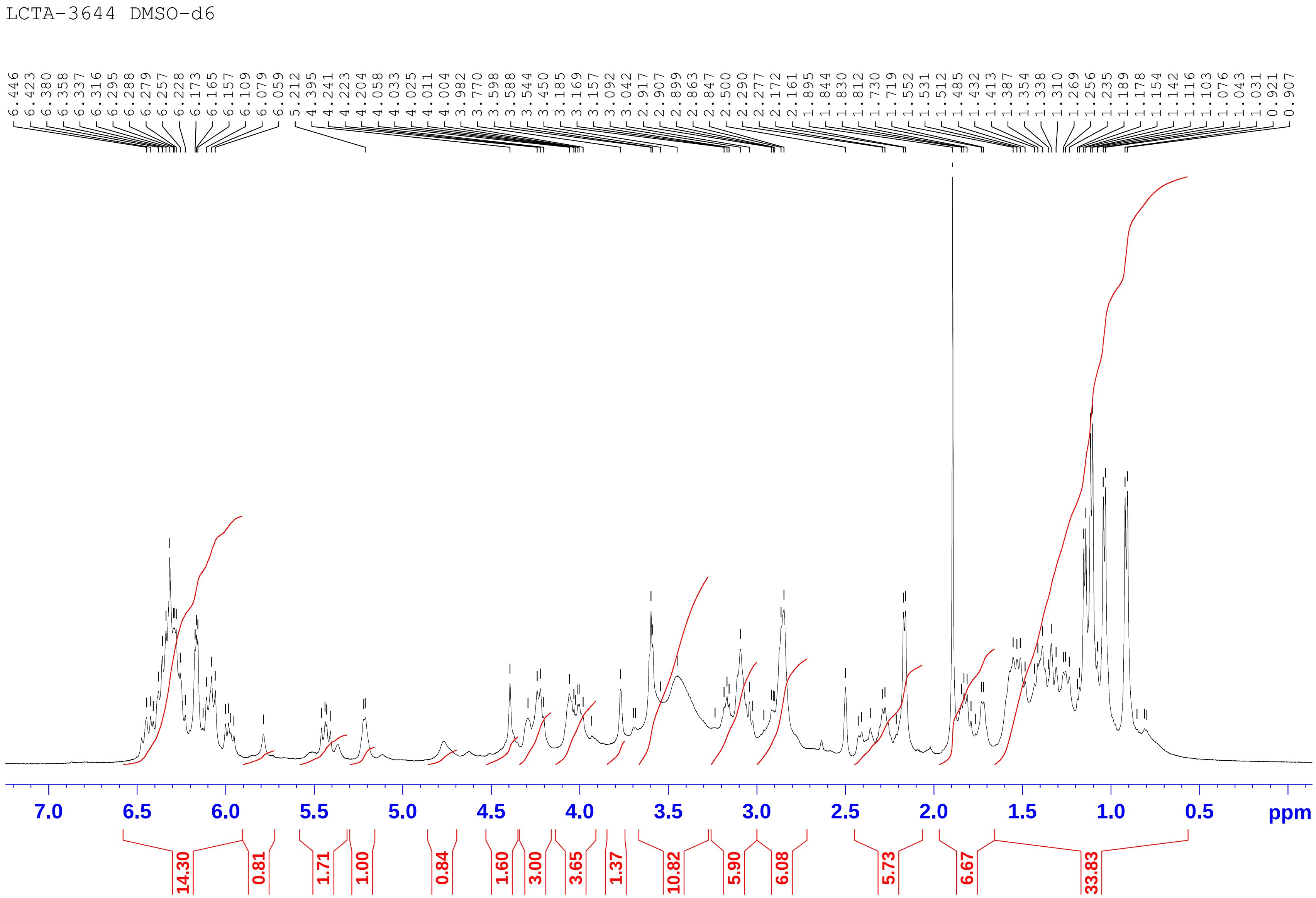

Figure S1. ^{1}H NMR spectra of the AmB derivative **3.**

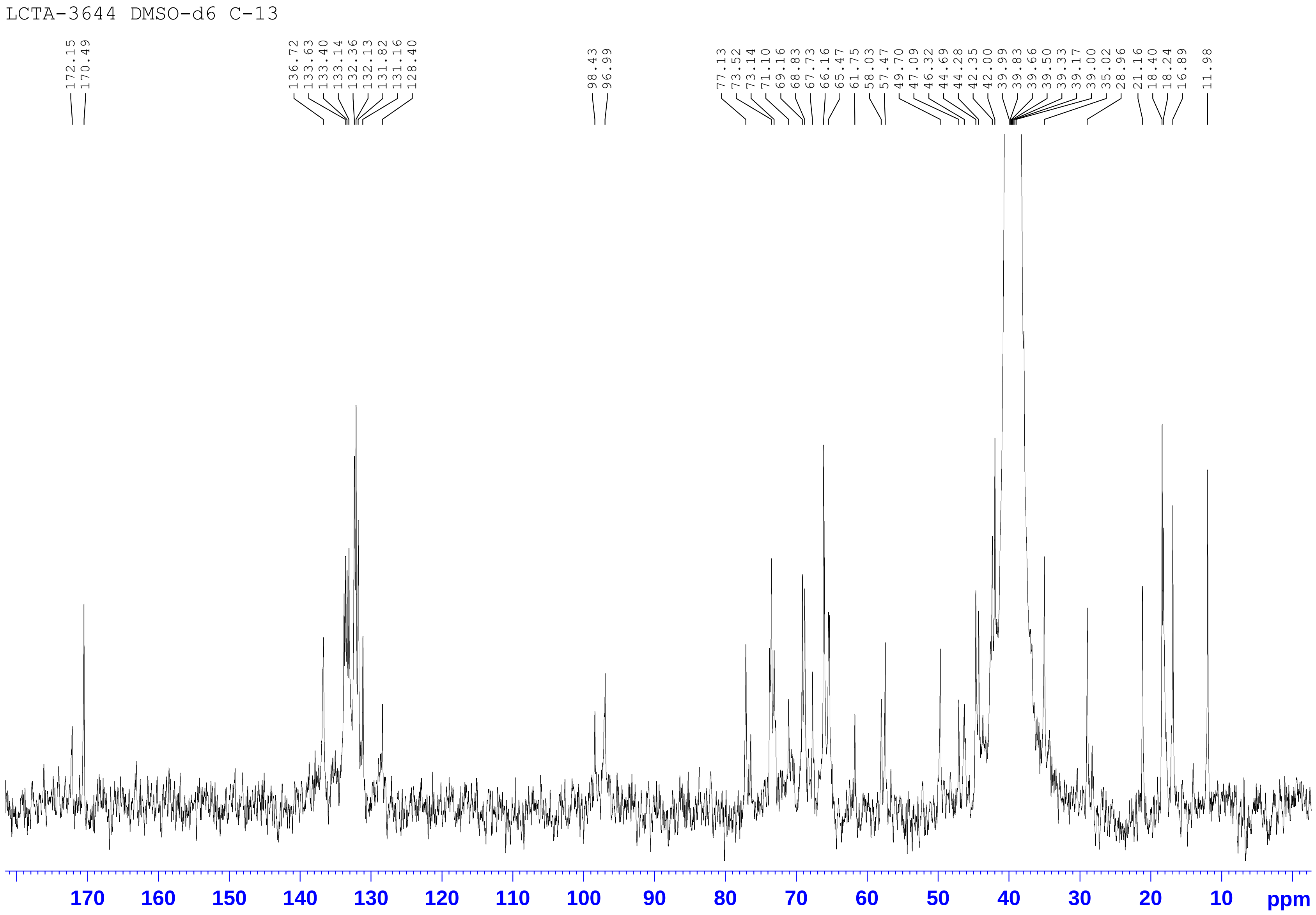

Figure S2. ^{13}C NMR spectra of the AmB derivative **3.**

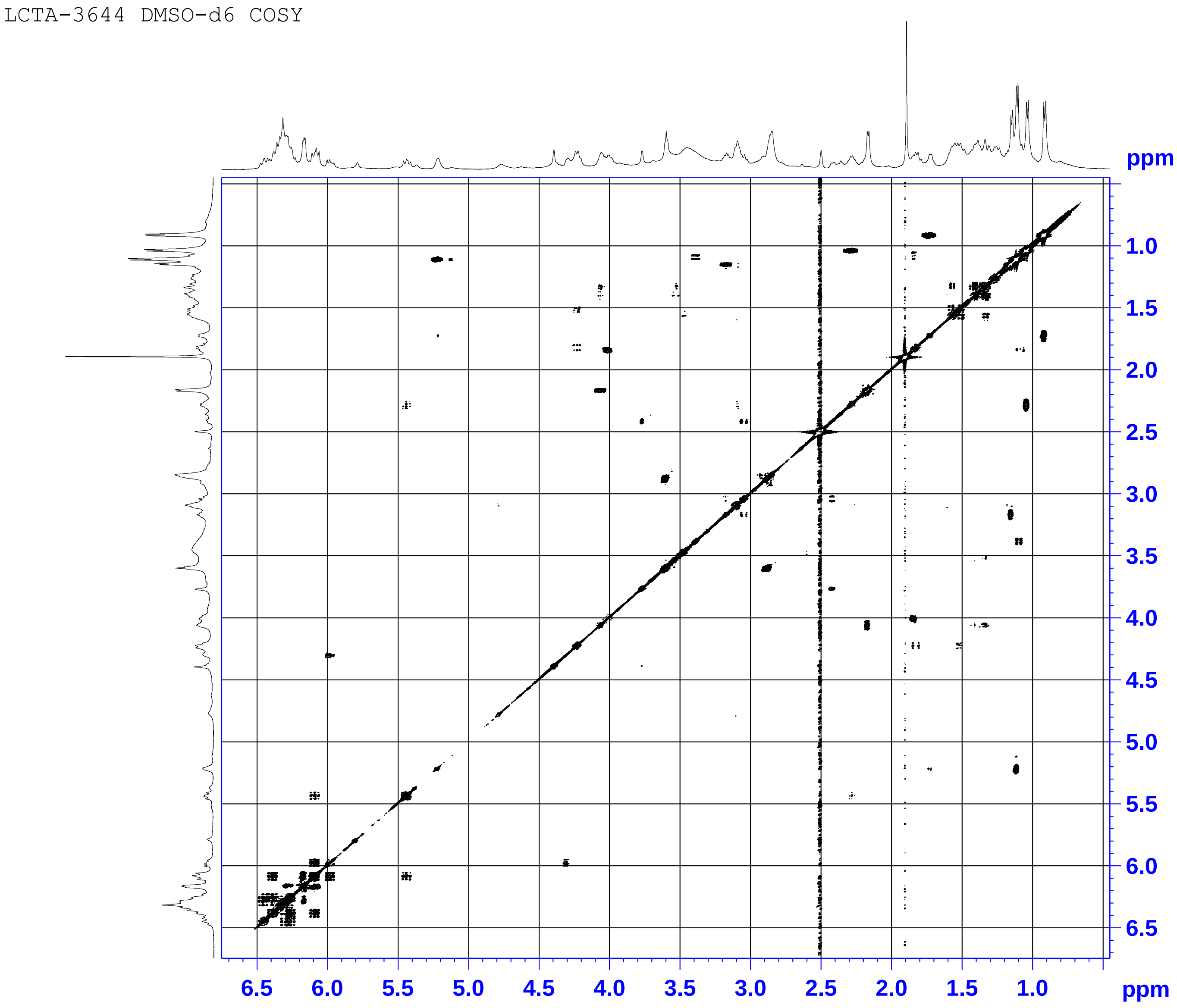

Figure S3. ^{1}H-^{1}H COSY spectra of the AmB derivative **3**.

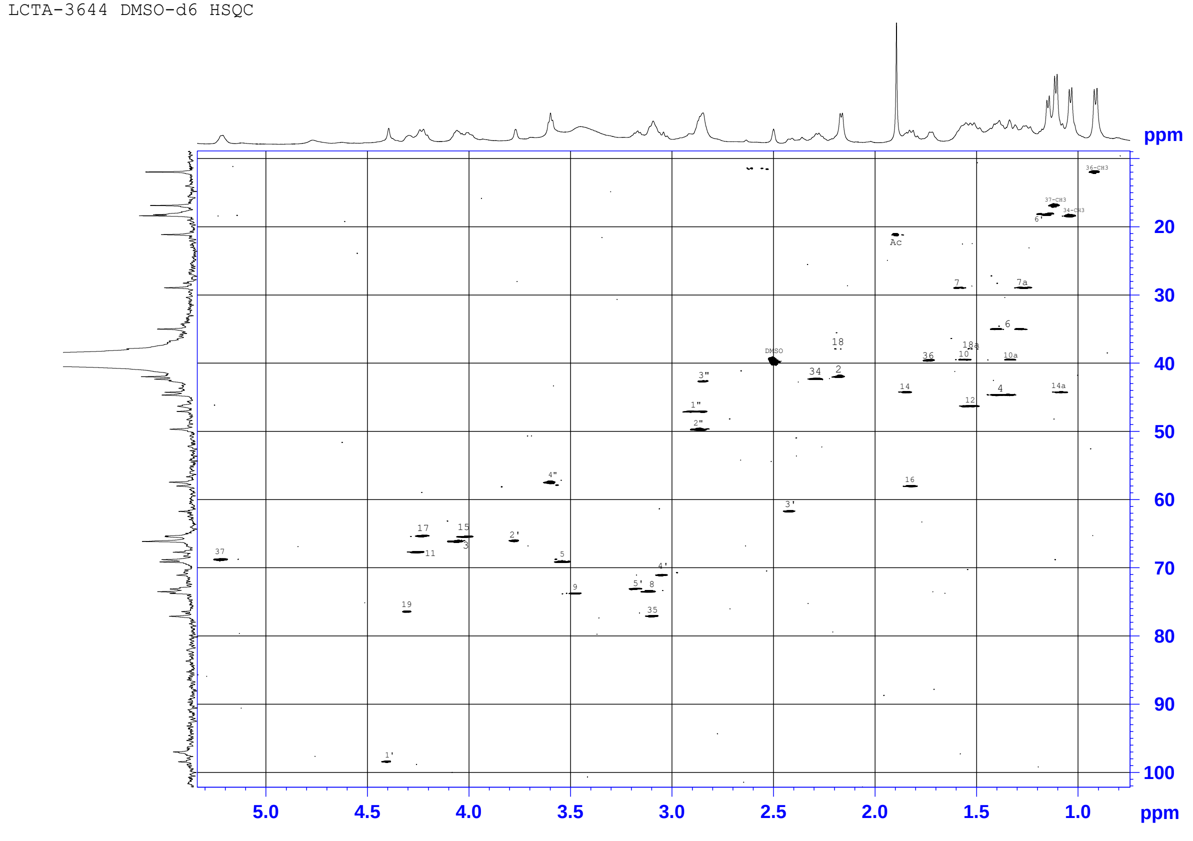

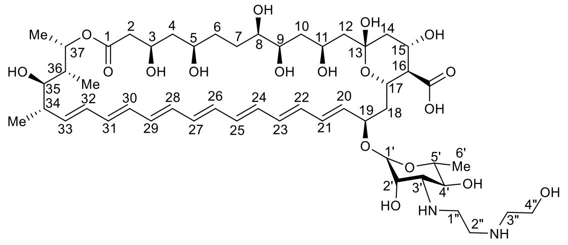

Structure and atom numbering of derivative **3**

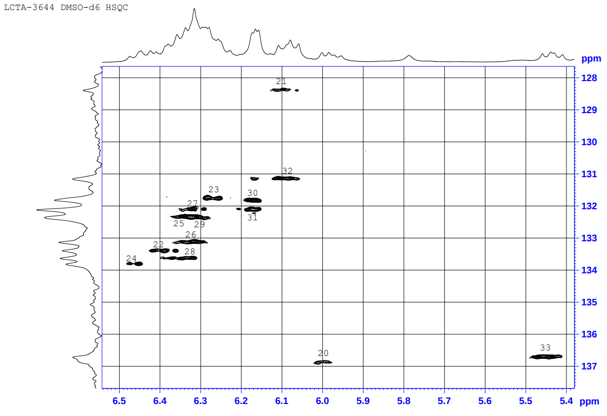

Figure S4. ^{1}H-^{13}C HSQC NMR spectra of the AmB derivative **3**.

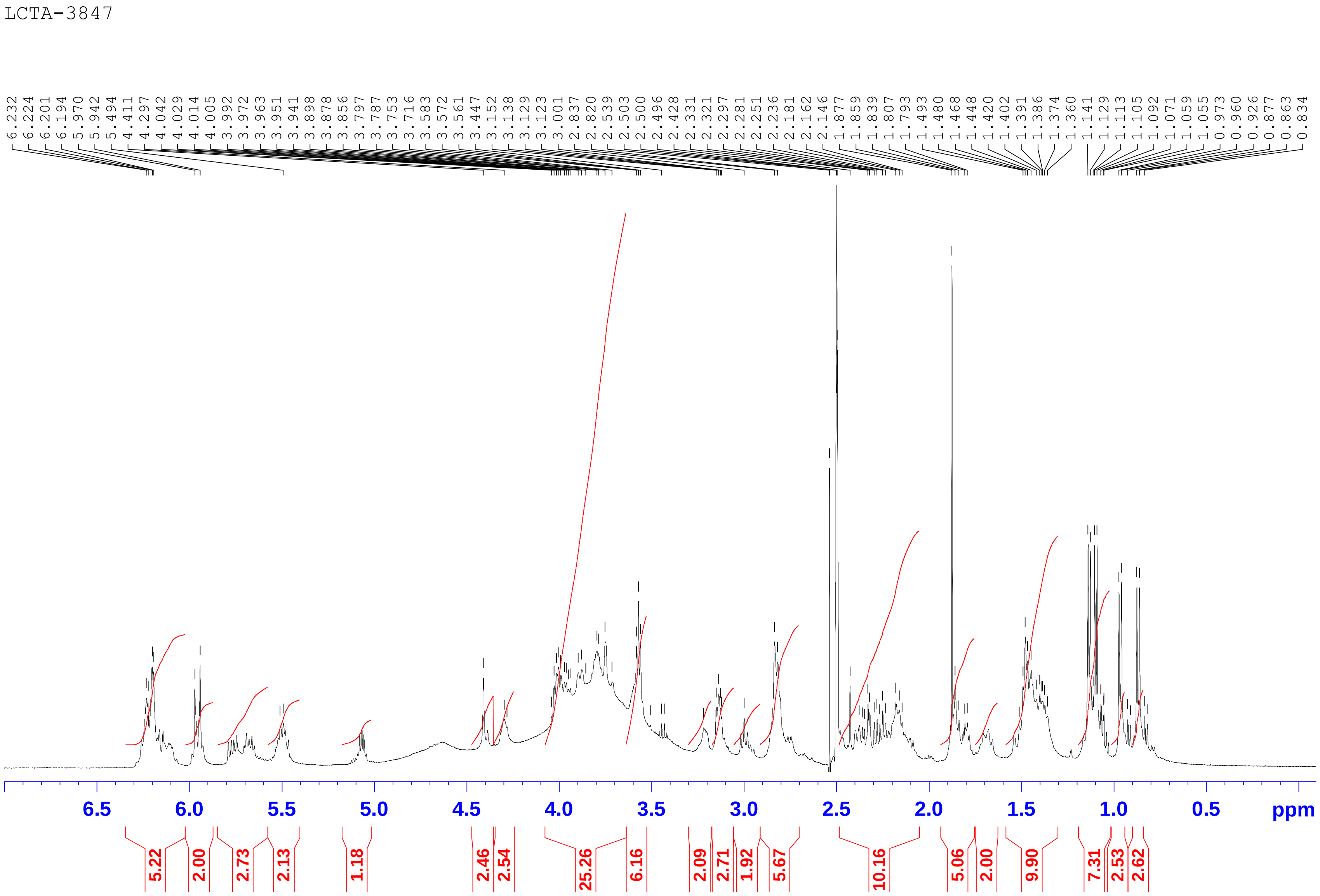

Figure S5. ^{1}H NMR spectra of the Nys derivative **4**.

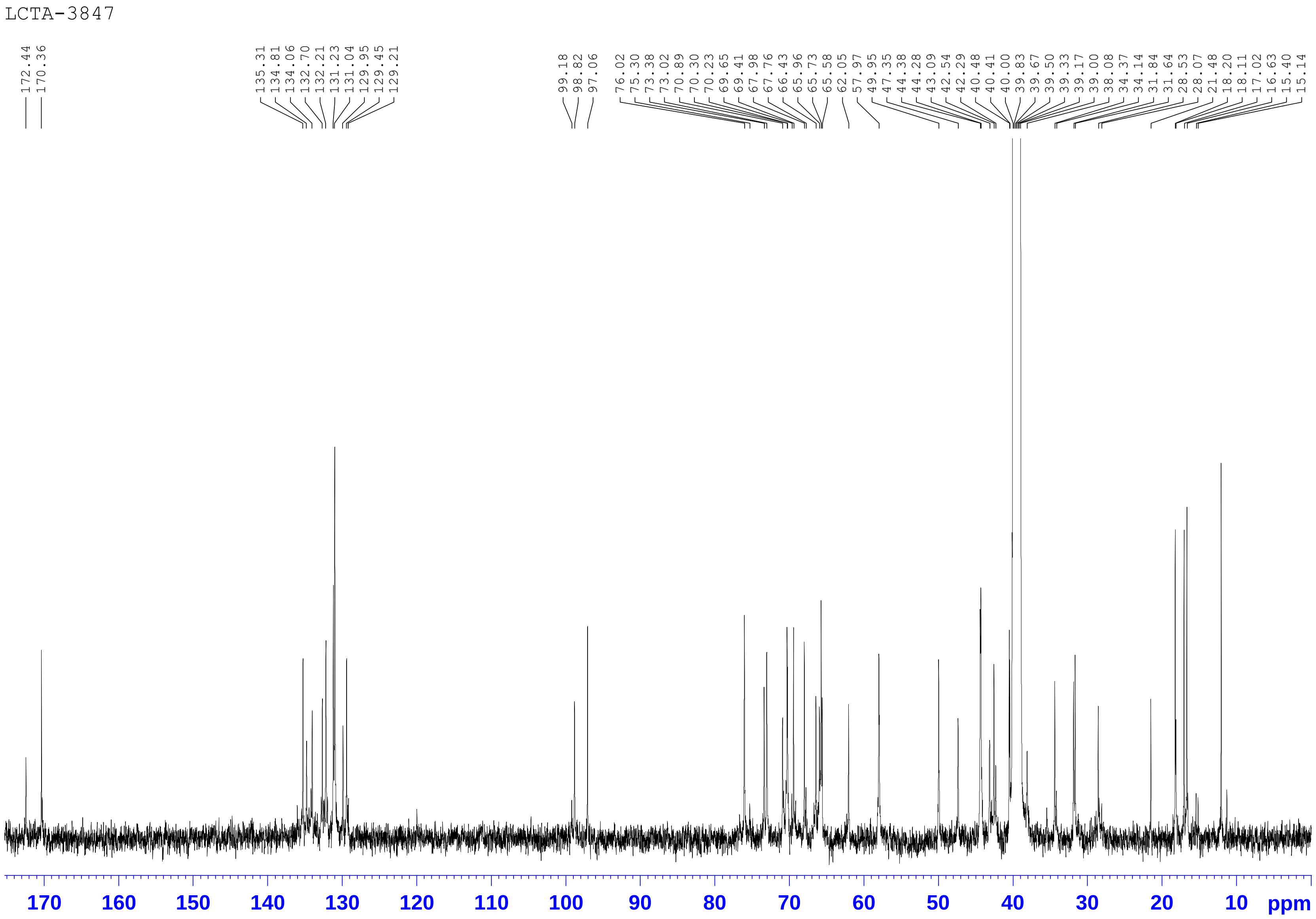

Figure S6. ^{13}C NMR spectra of the Nys derivative **4**.

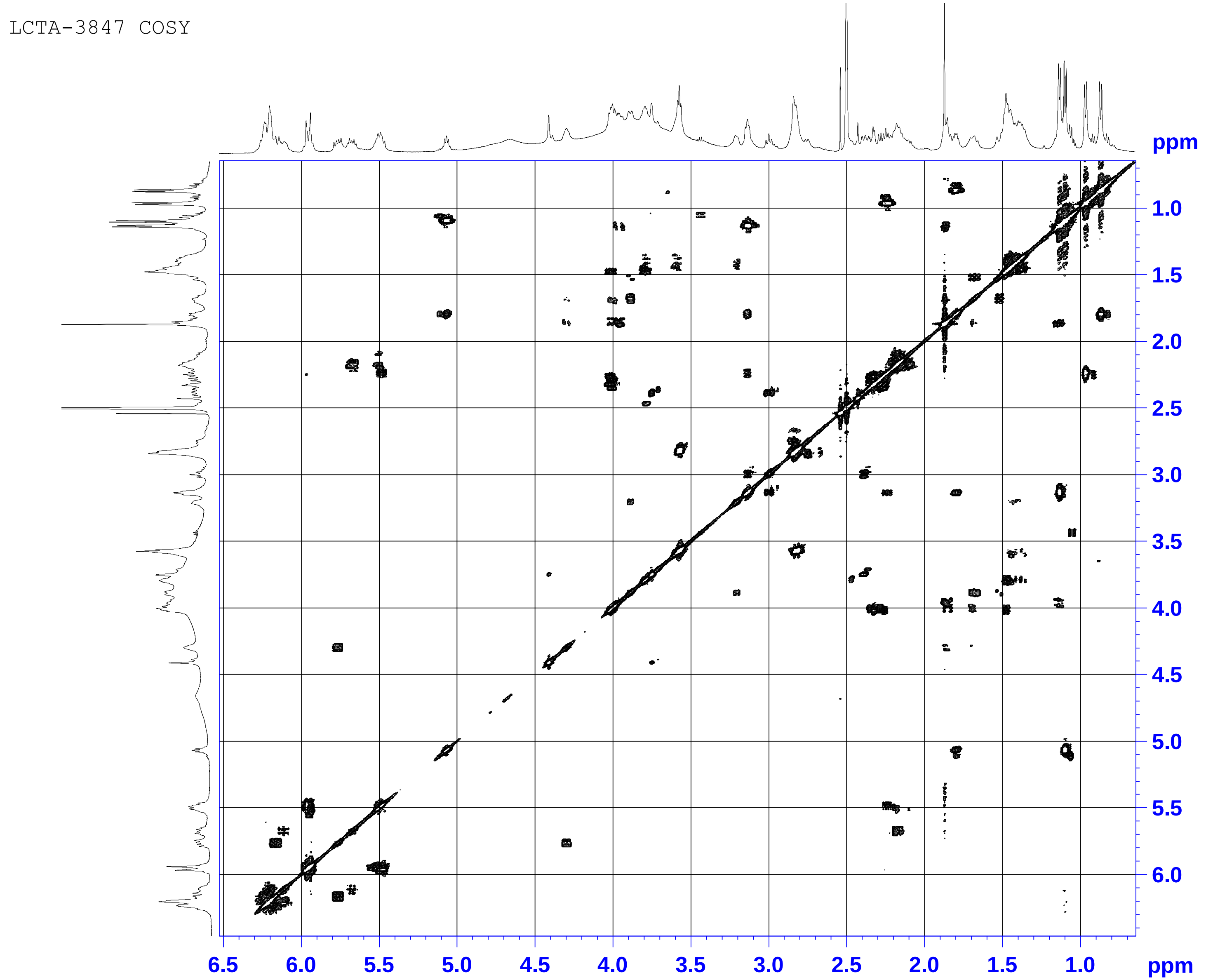

Figure S7. ^{1}H-^{1}H COSY spectra of the Nys derivative **4**.

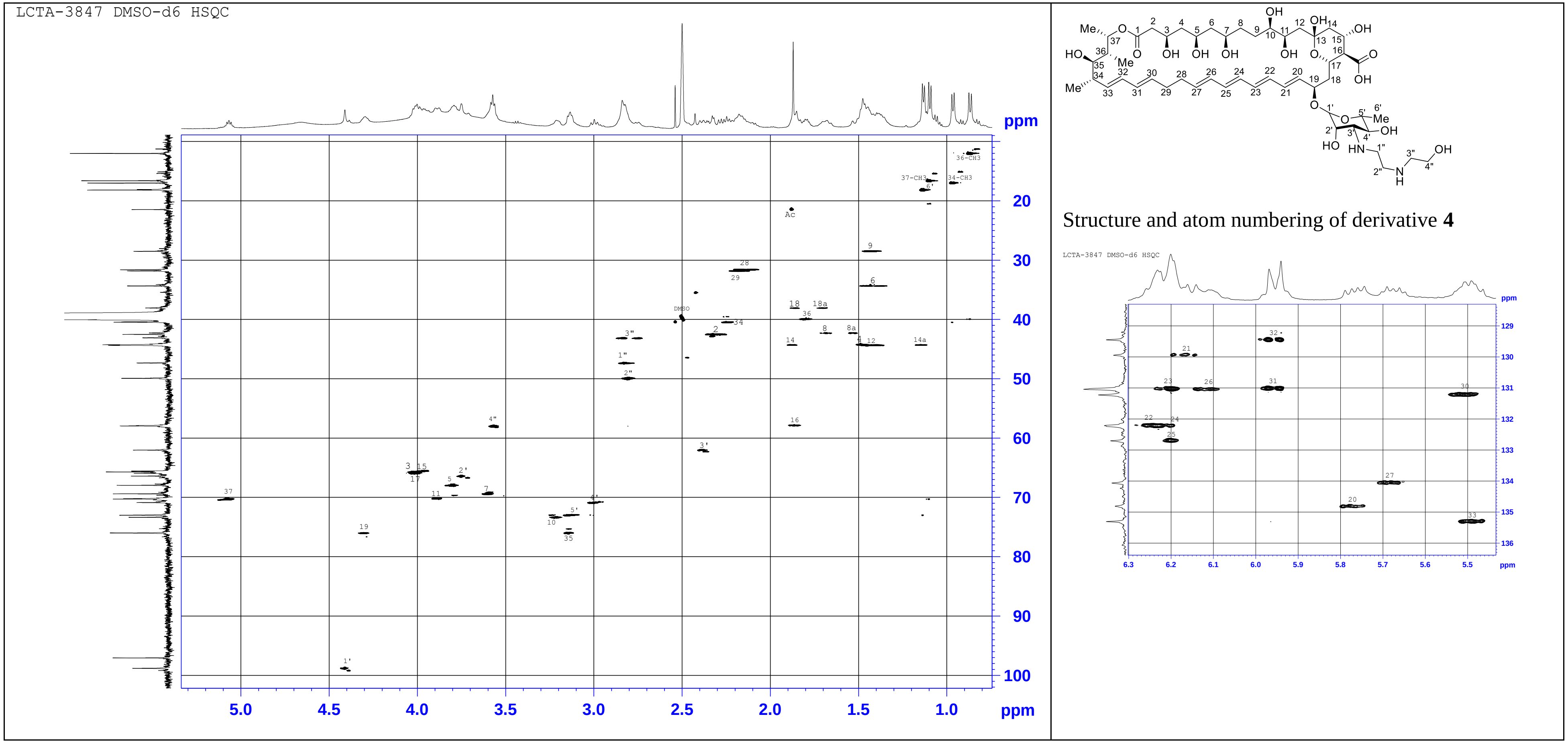

Figure S8. ^{1}H-^{13}C HSQC NMR spectra of the Nys derivative **4**.

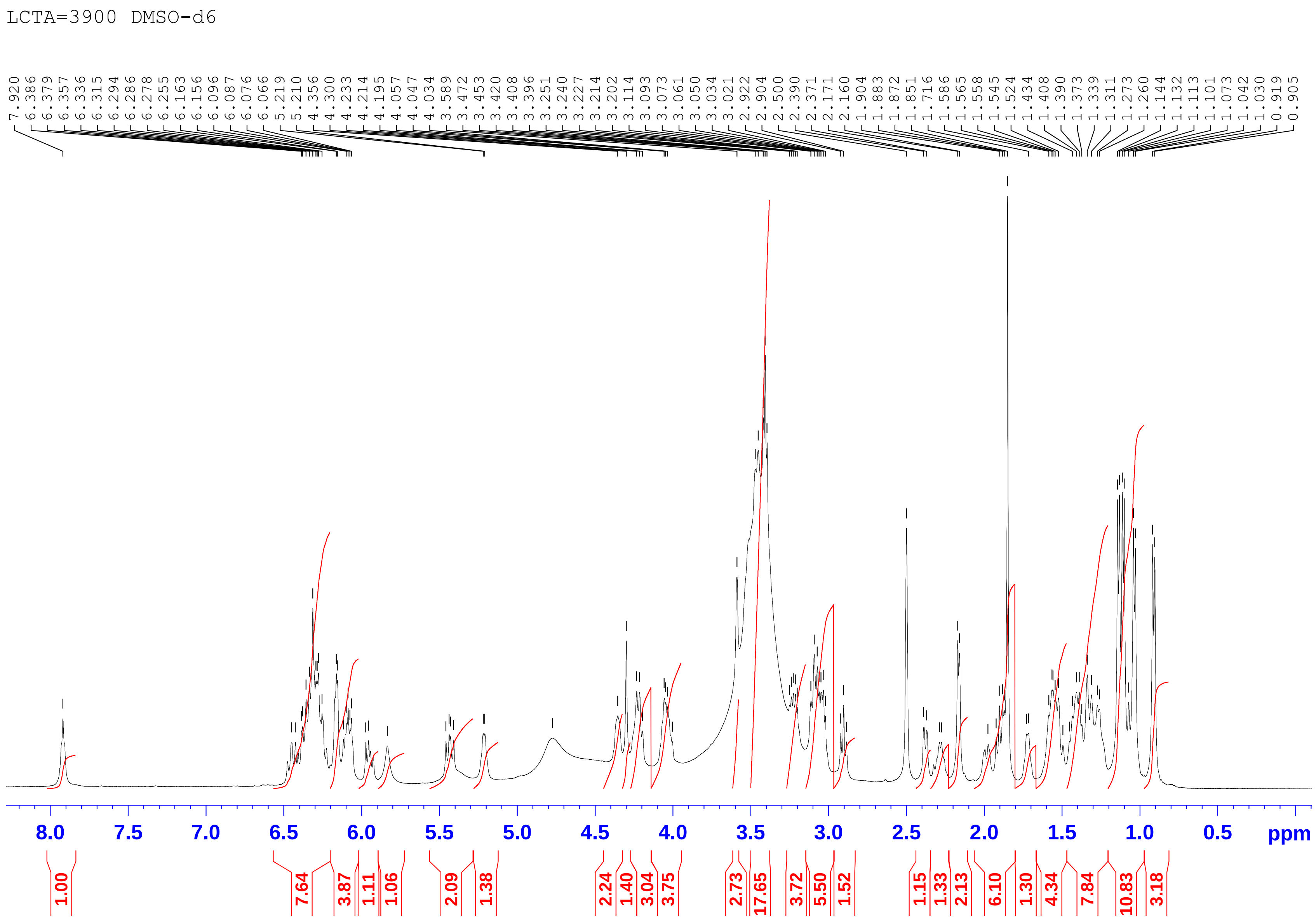

Figure S9. ^{1}H NMR spectra of the AmB derivative **5**.

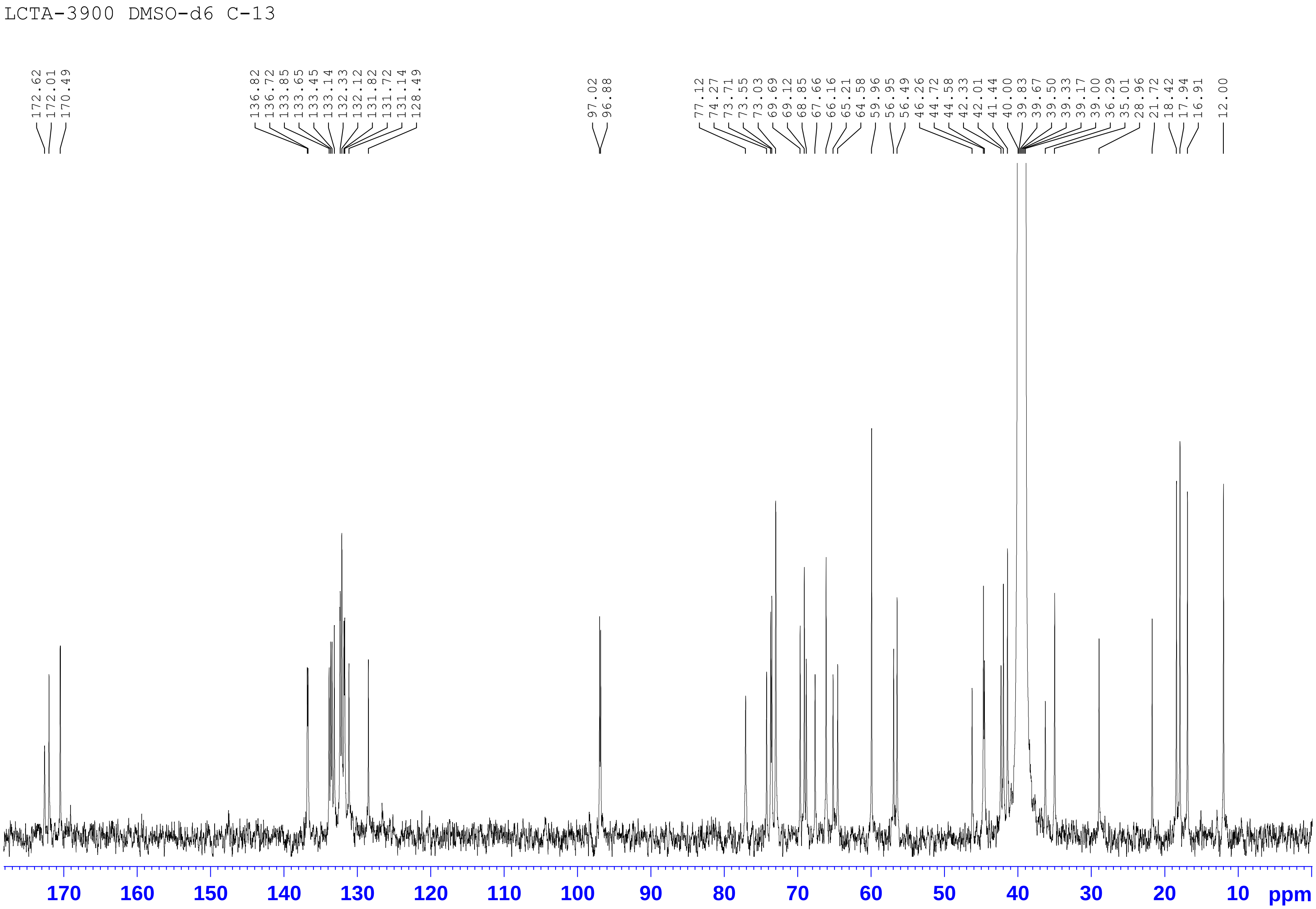

Figure S10. ^{13}C NMR spectra of the AmB derivative **5**.

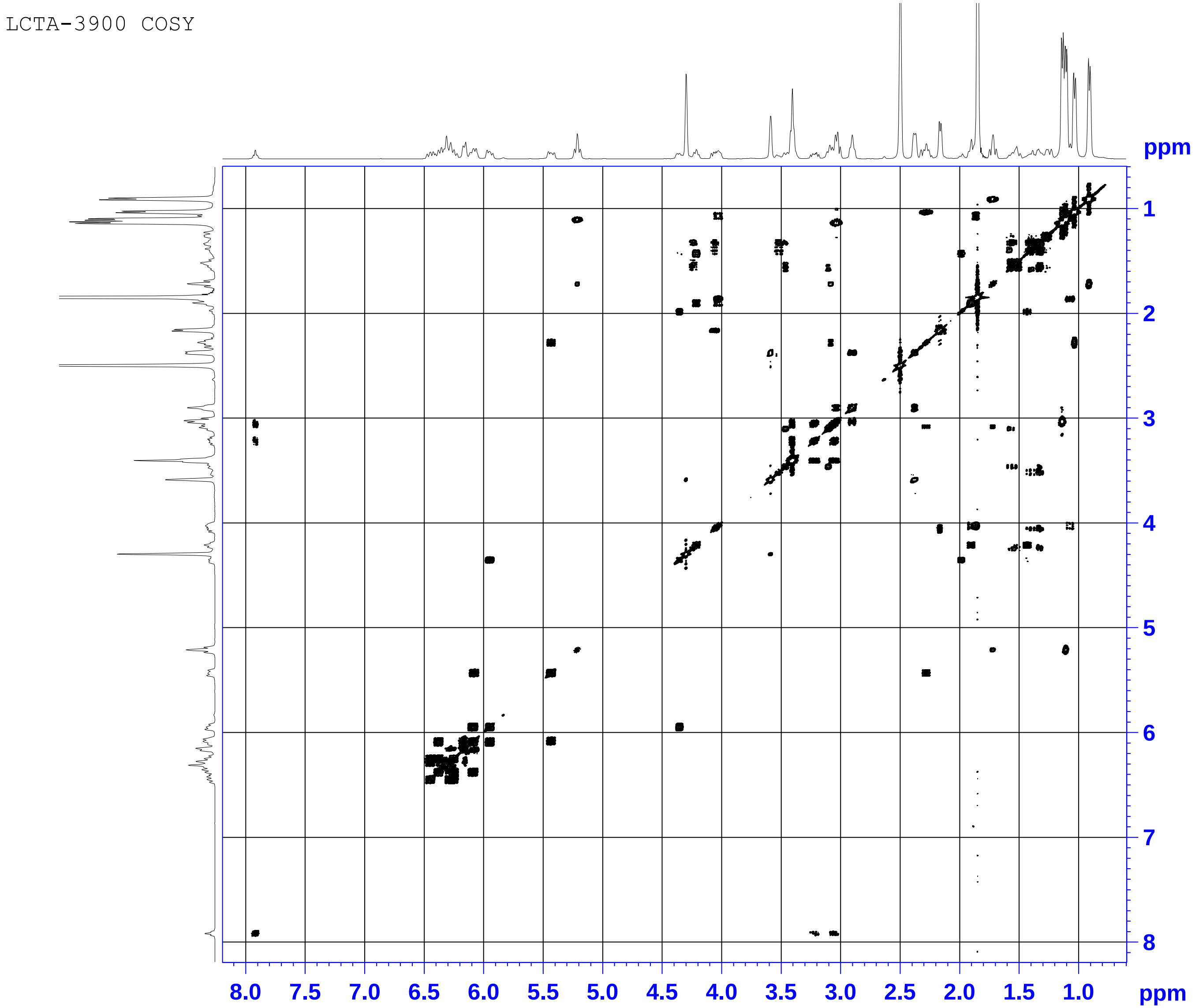

Figure S11. ^{1}H-^{1}H COSY spectra of the AmB derivative **5**.

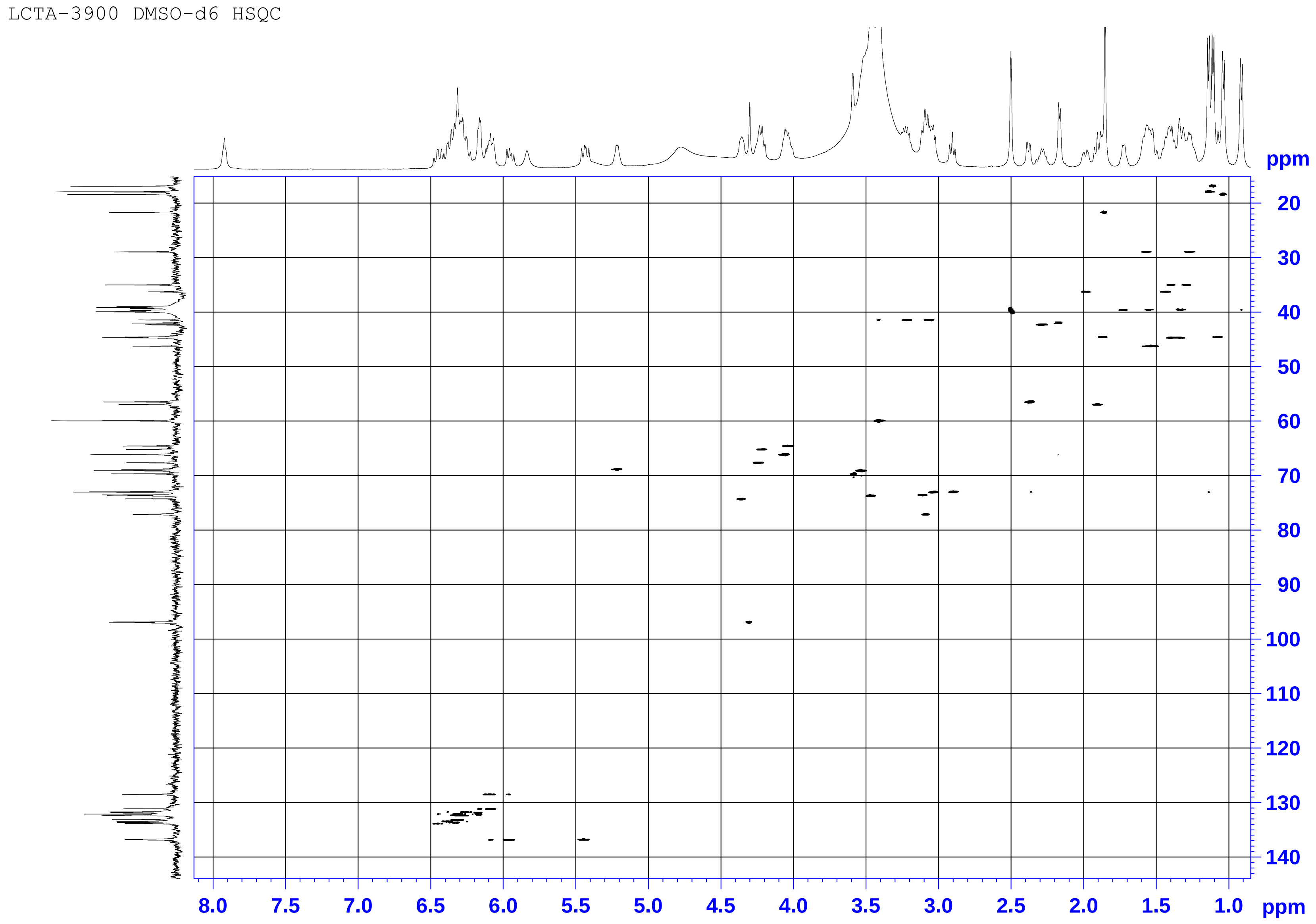

Figure S12. ^{1}H-^{13}C HSQC NMR spectra of the AmB derivative **5**.

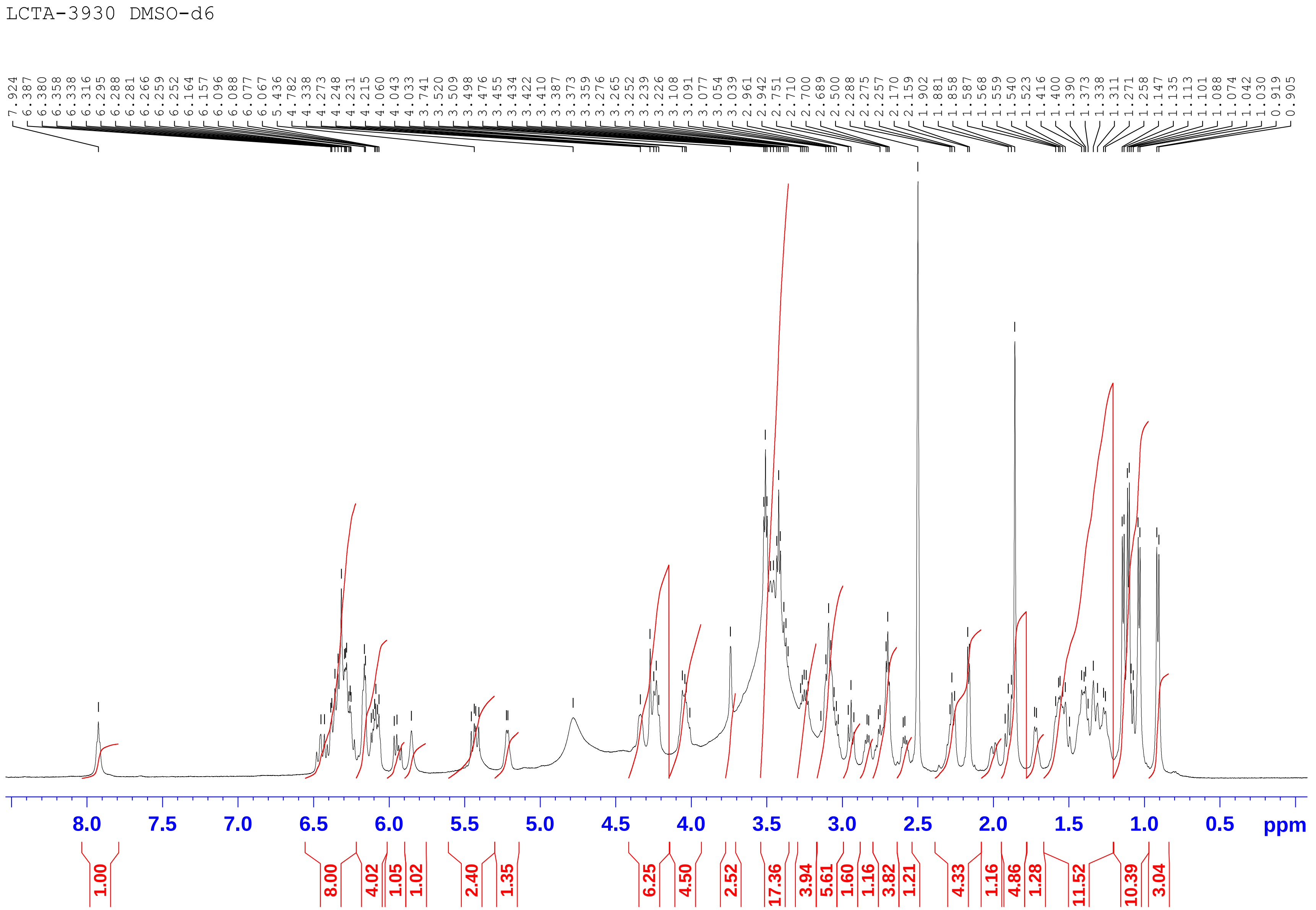

Figure S13. ^{1}H NMR spectra of the AmB derivative **6**.

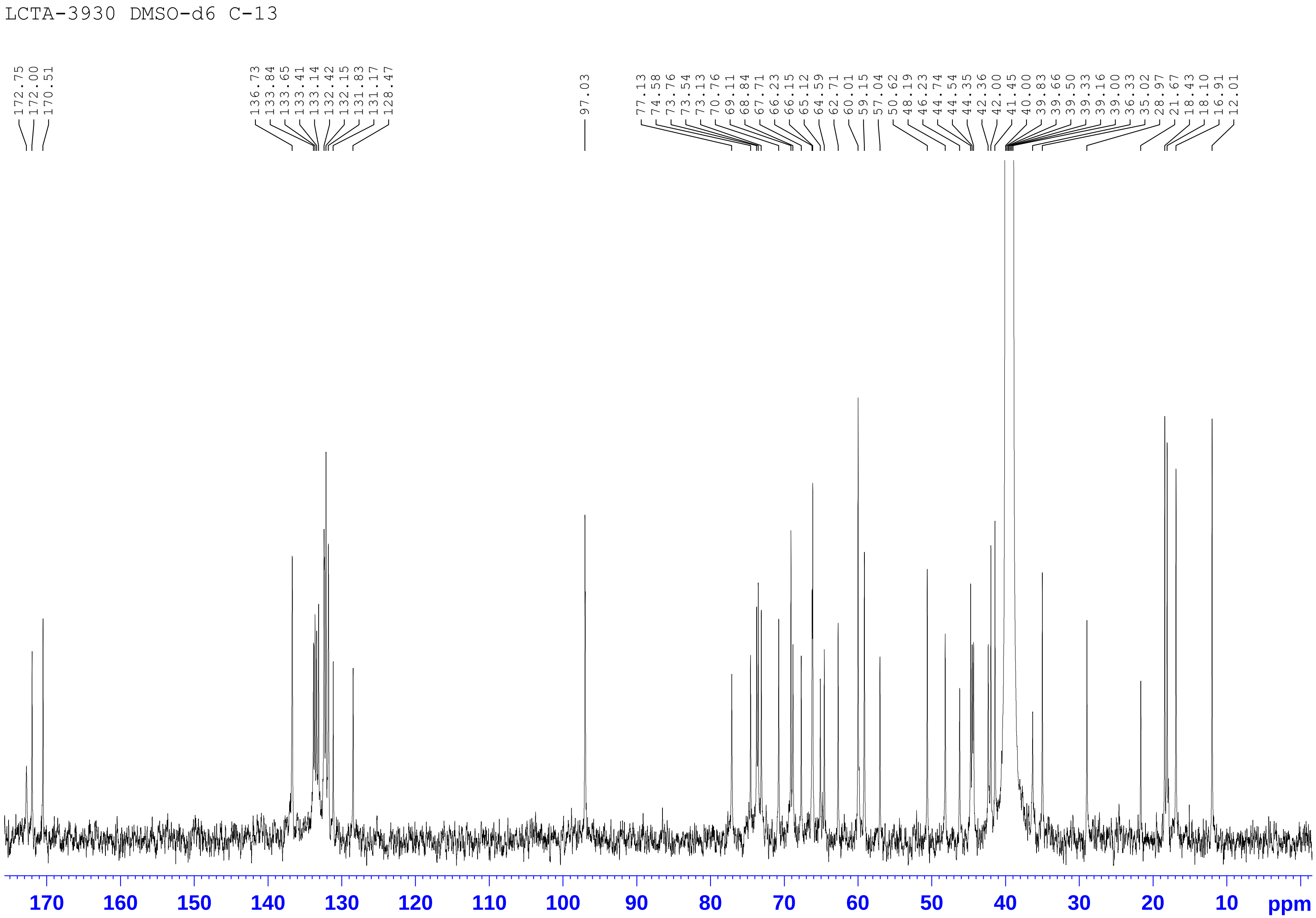

Figure S14. ^{13}C NMR spectra of the AmB derivative **6**.

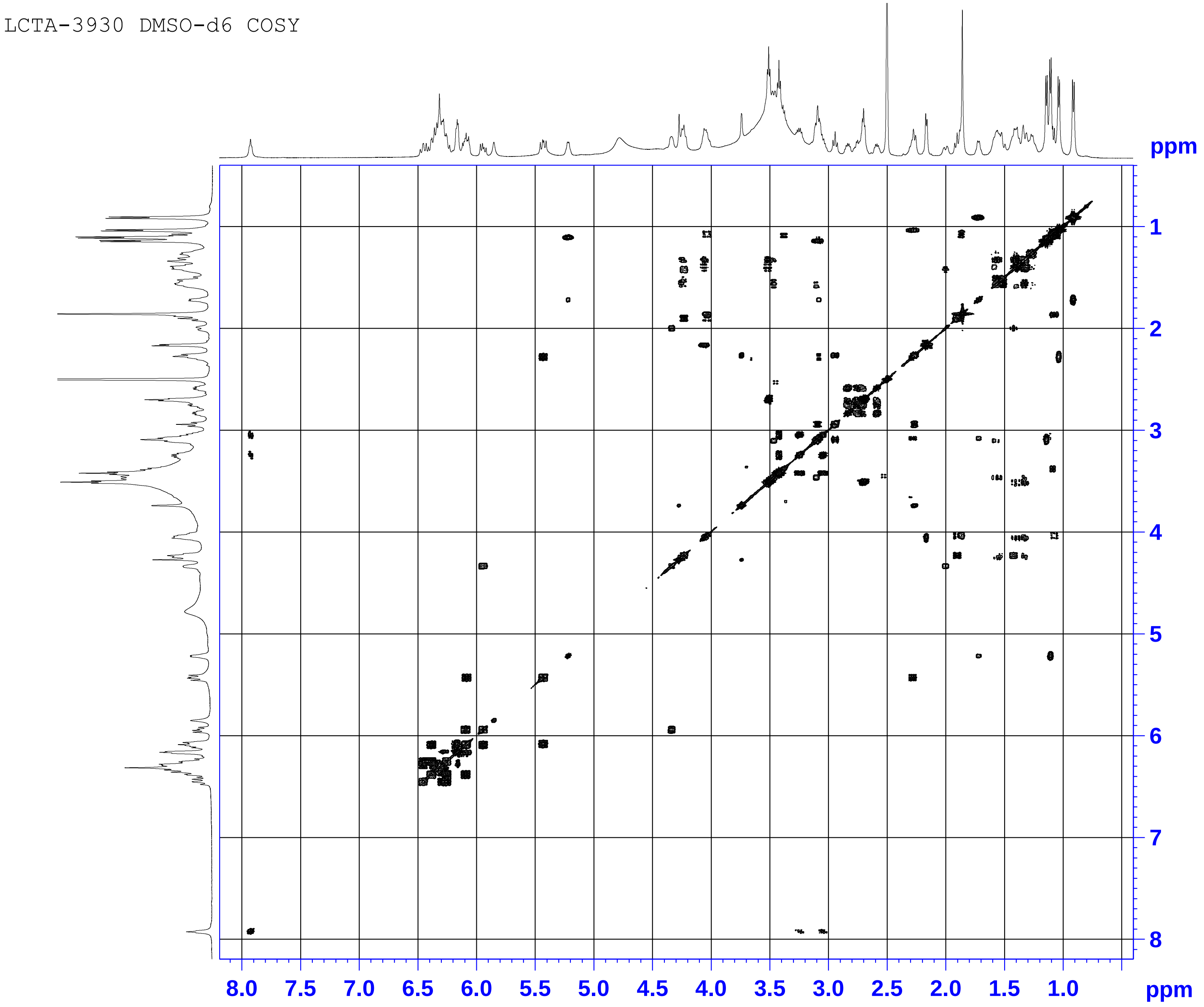

Figure S15. ^{1}H-^{1}H COSY spectra of the AmB derivative **6**.

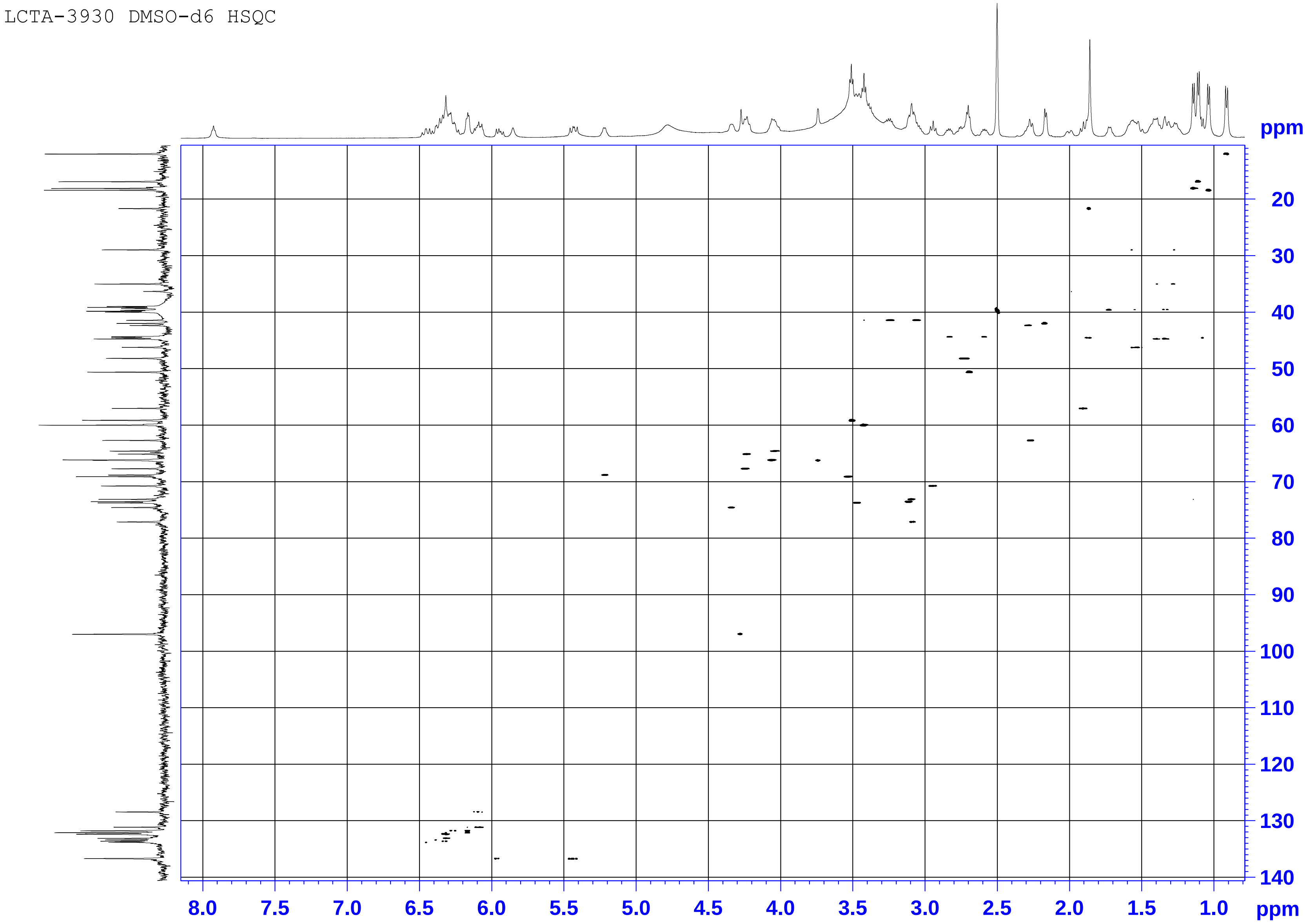

Figure S16. ^{1}H-^{13}C HSQC NMR spectra of the AmB derivative **6**.

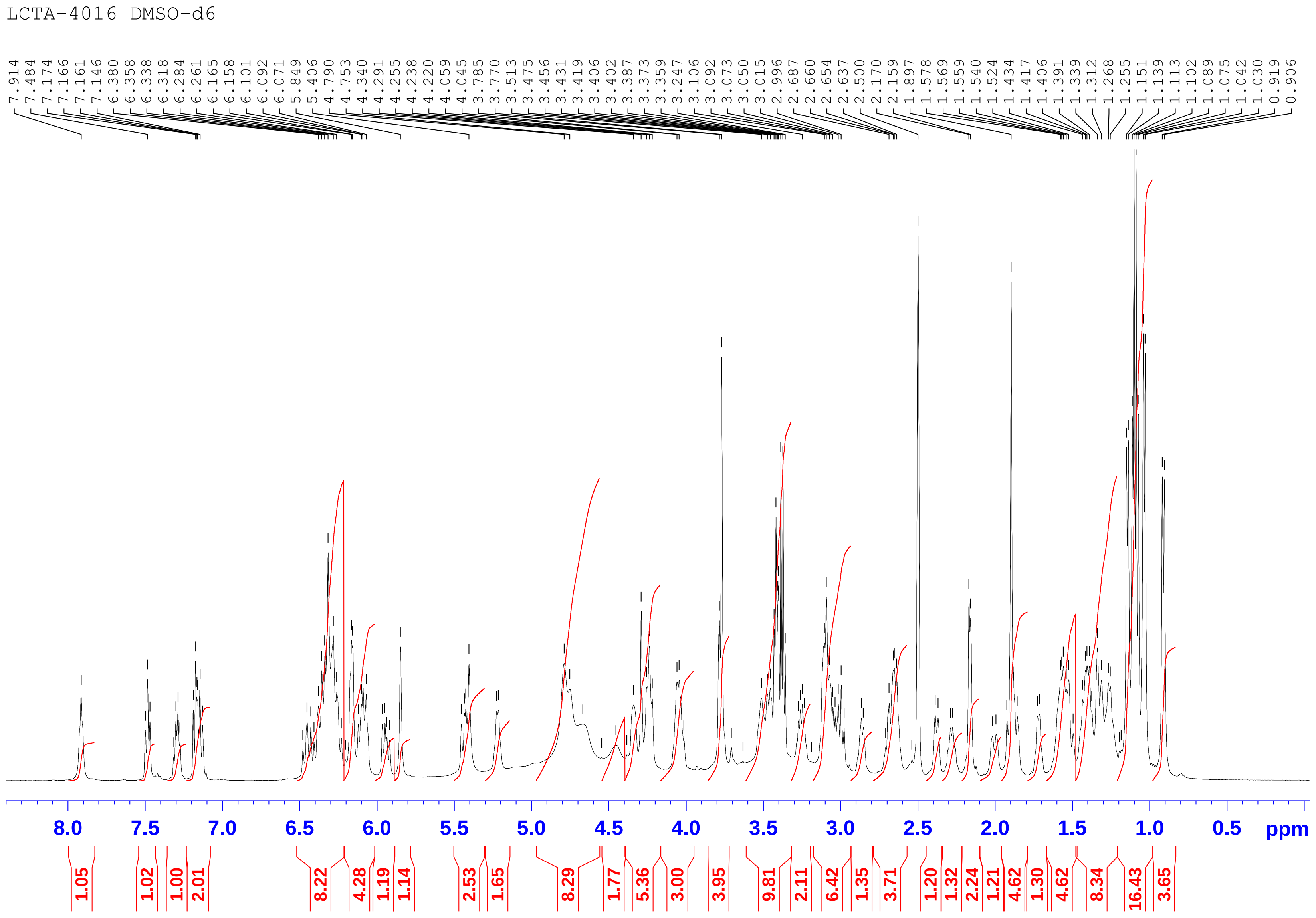

Figure S17. ^{1}H NMR spectra of the AmB derivative **7**.

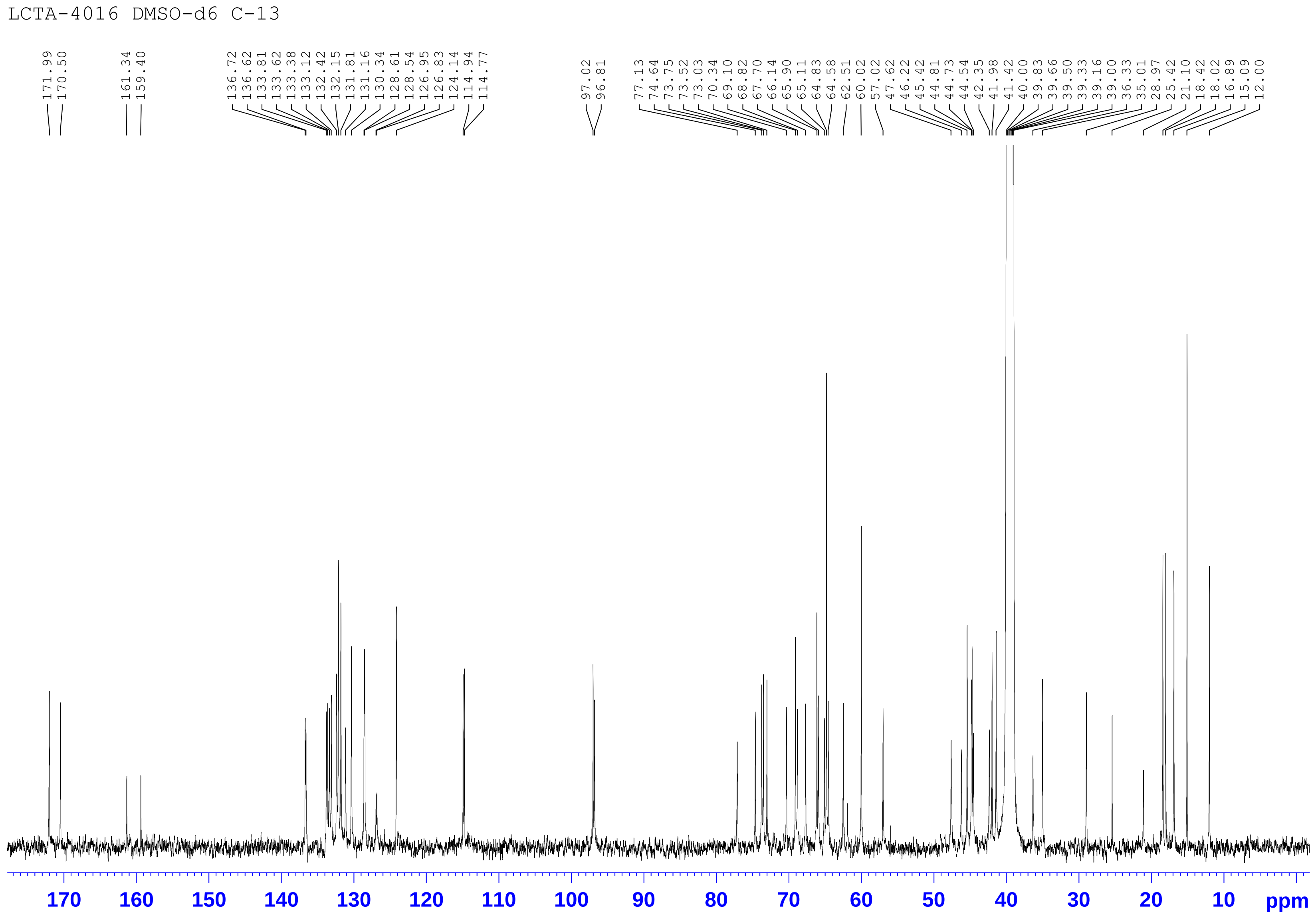

Figure S18. ^{13}C NMR spectra of the AmB derivative **7**.

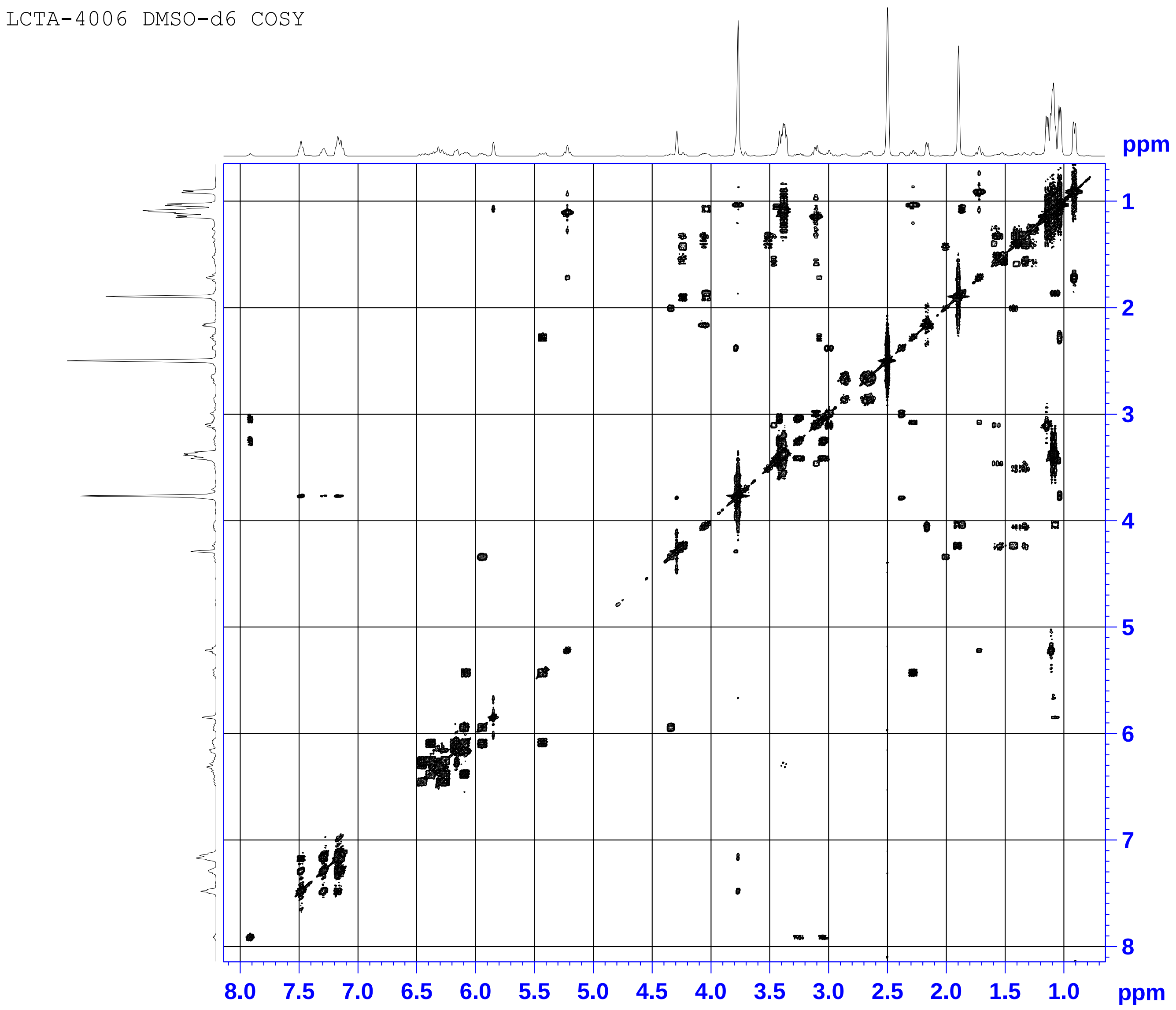

Figure S19. ^{1}H-^{1}H COSY spectra of the AmB derivative **7**.

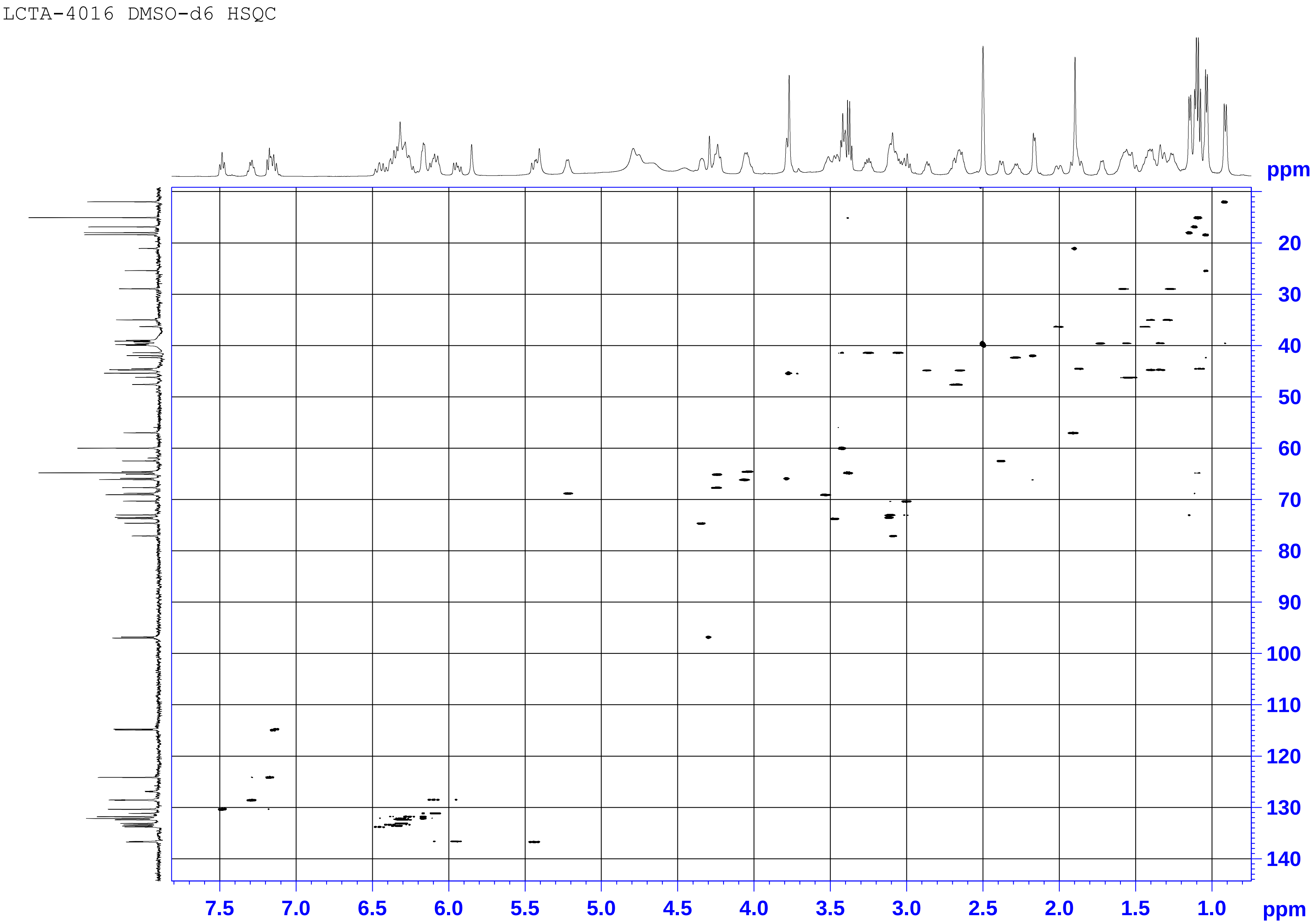

Figure S20. ^{1}H-^{13}C HSQC NMR spectra of the AmB derivative **7**.

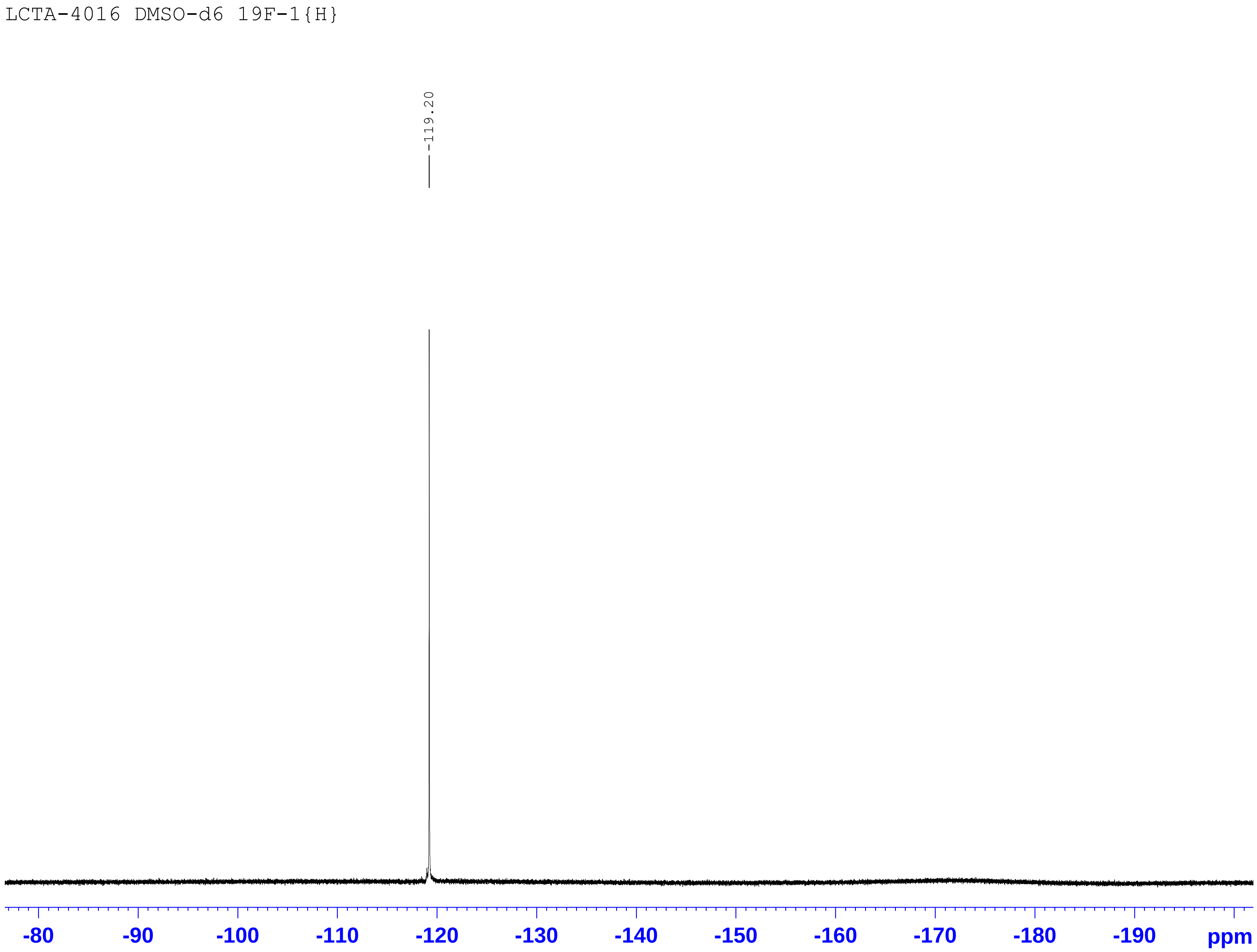

Figure S 21. ^{19}F-{1H} NMR spectra of the AmB derivative **7**.

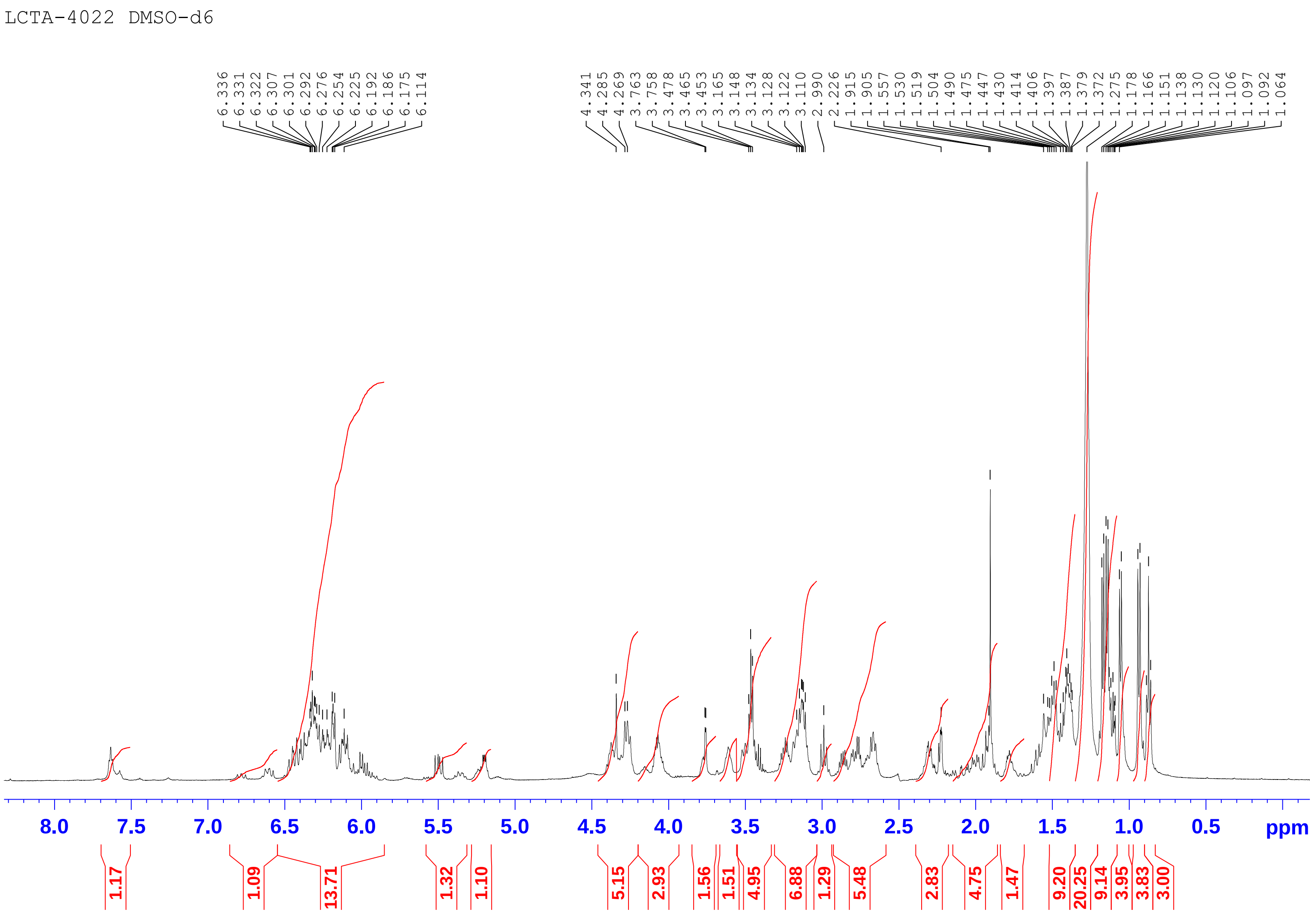

Figure S22. ^{1}H NMR spectra of the AmB derivative **8**.

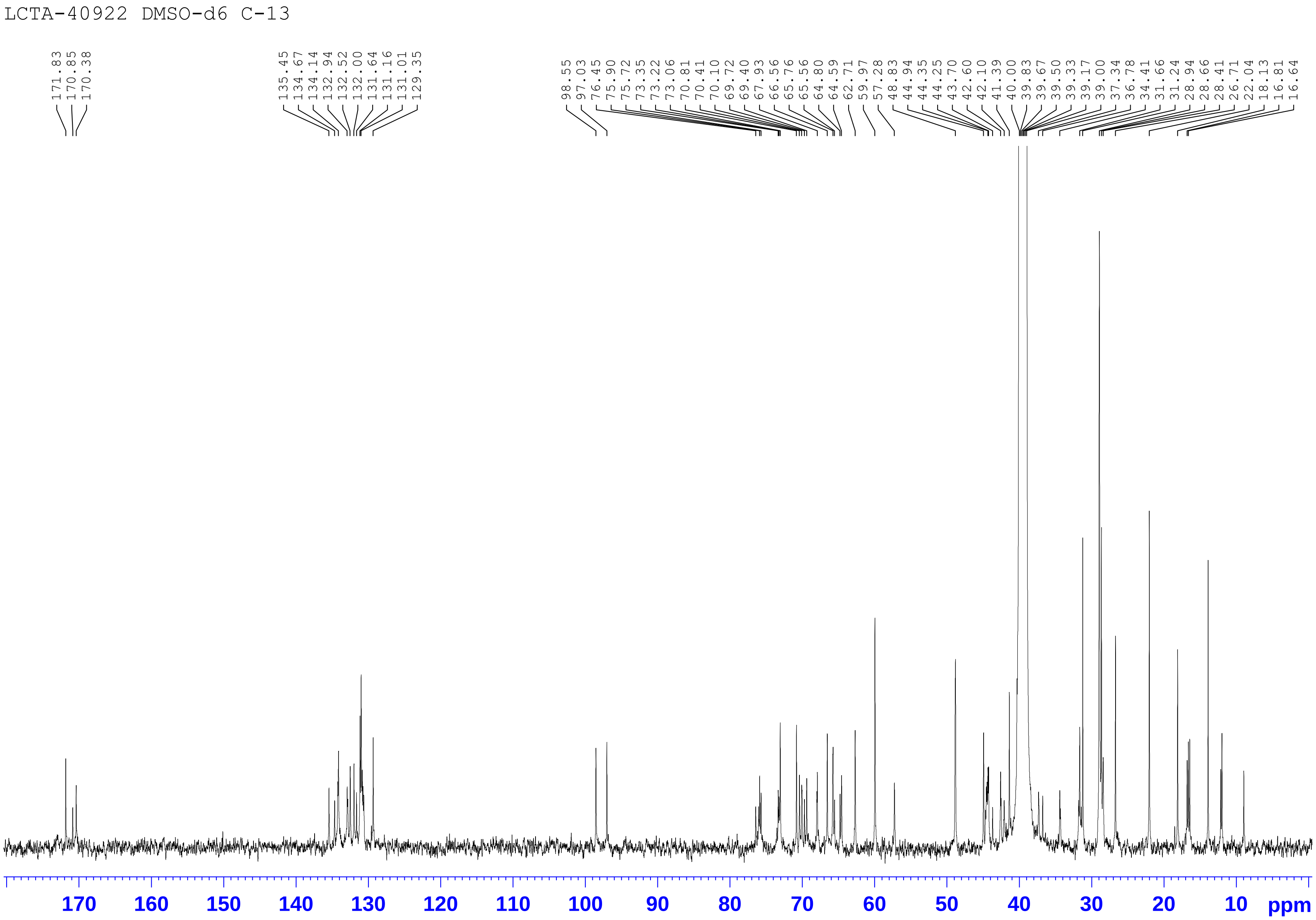

Figure S23. ^{13}C NMR spectra of the AmB derivative **8**.

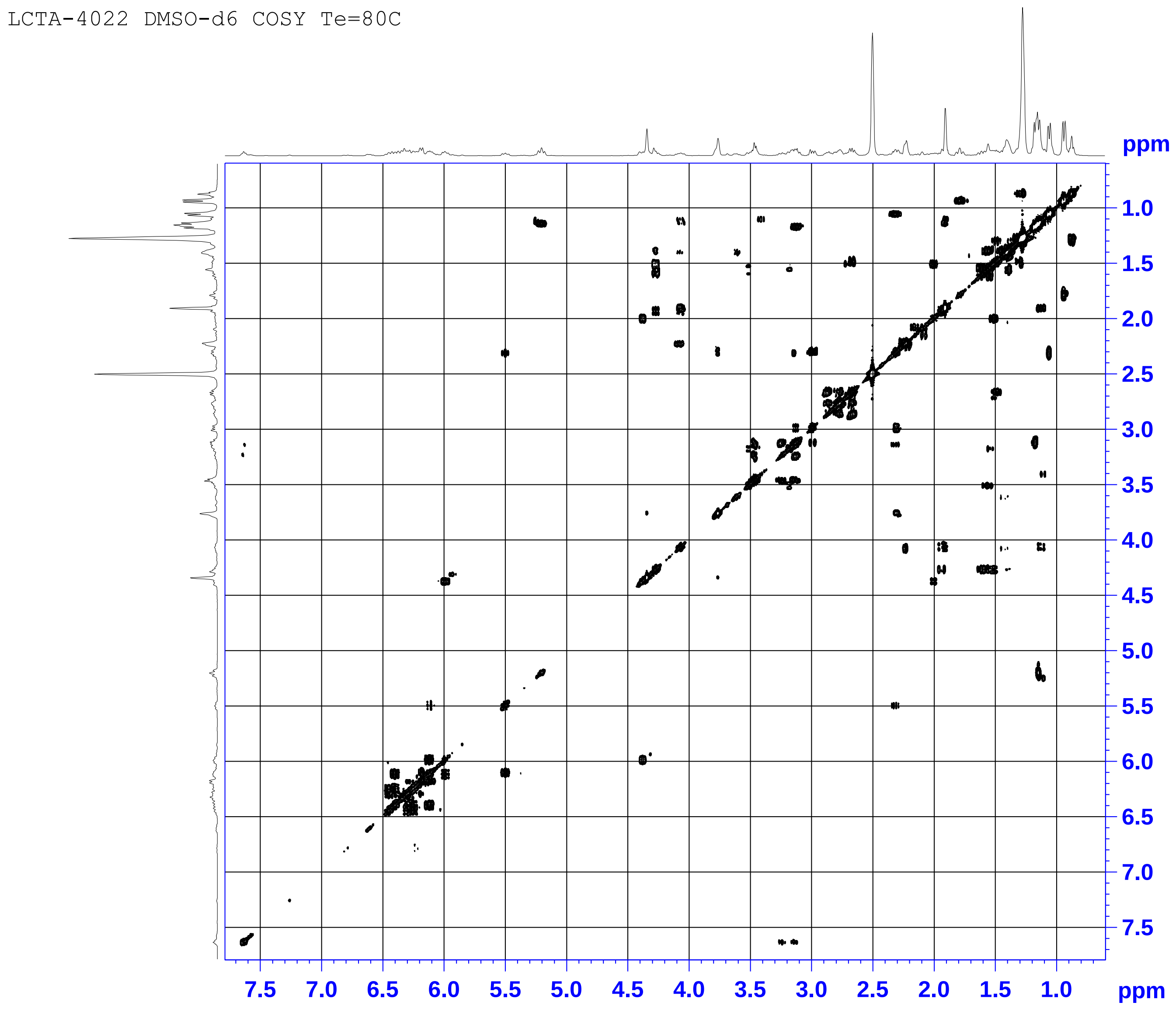

Figure S24. ^{1}H-^{1}H COSY spectra of the AmB derivative **8**.

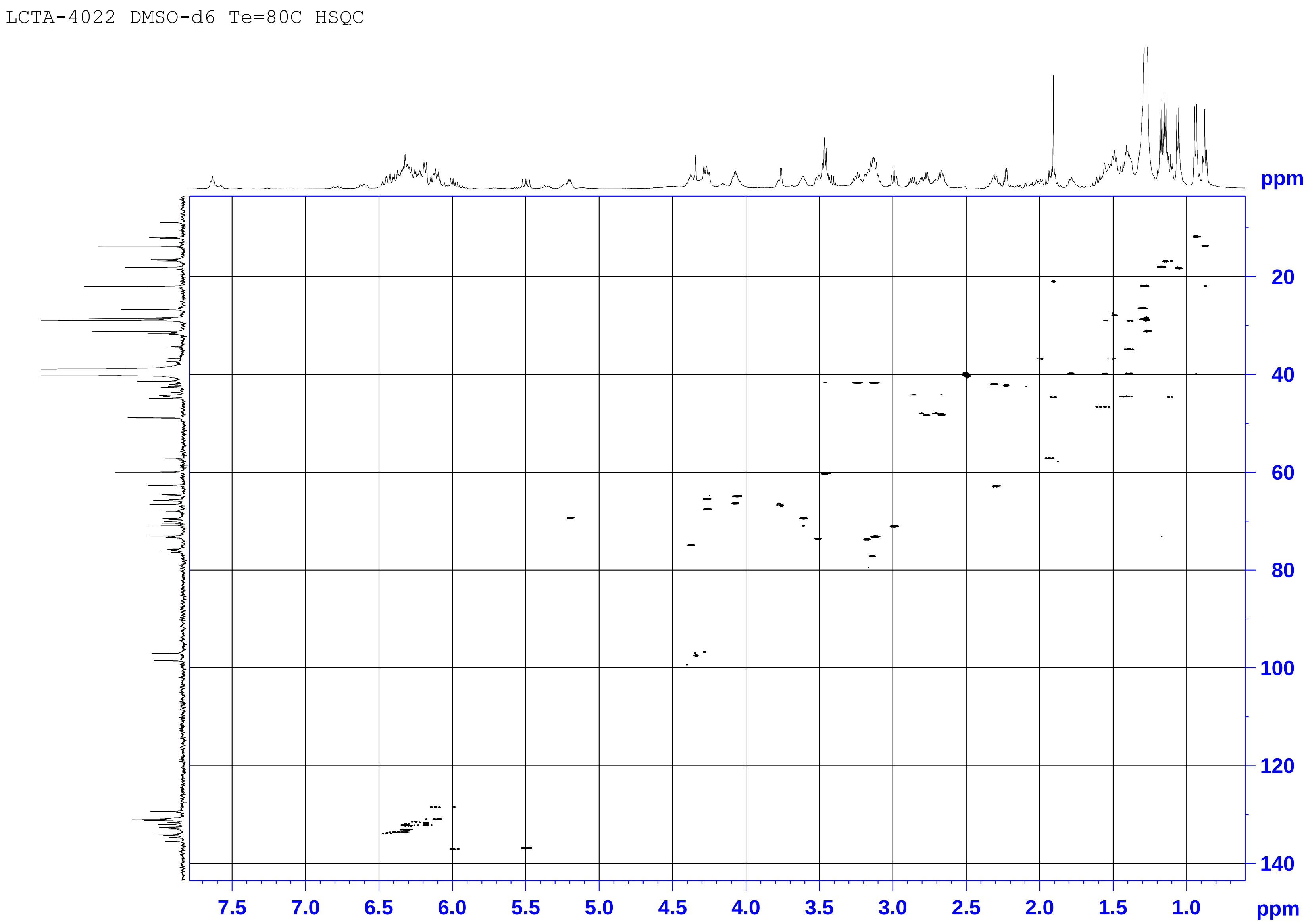

Figure S25. ^{1}H-^{13}C HSQC NMR spectra of the AmB derivative **8**.

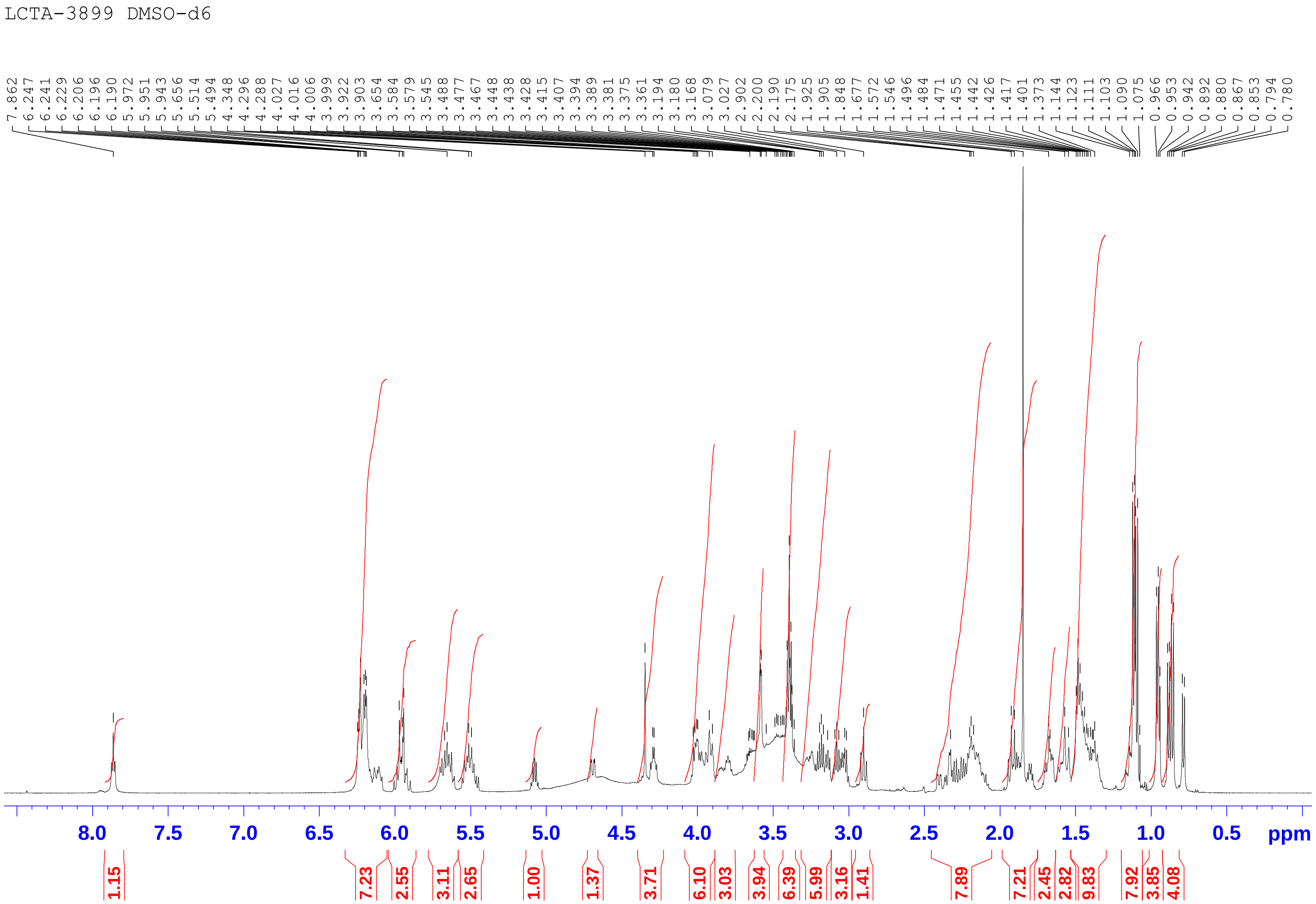

Figure S26. ^{1}H NMR spectra of the Nys derivative **9**.

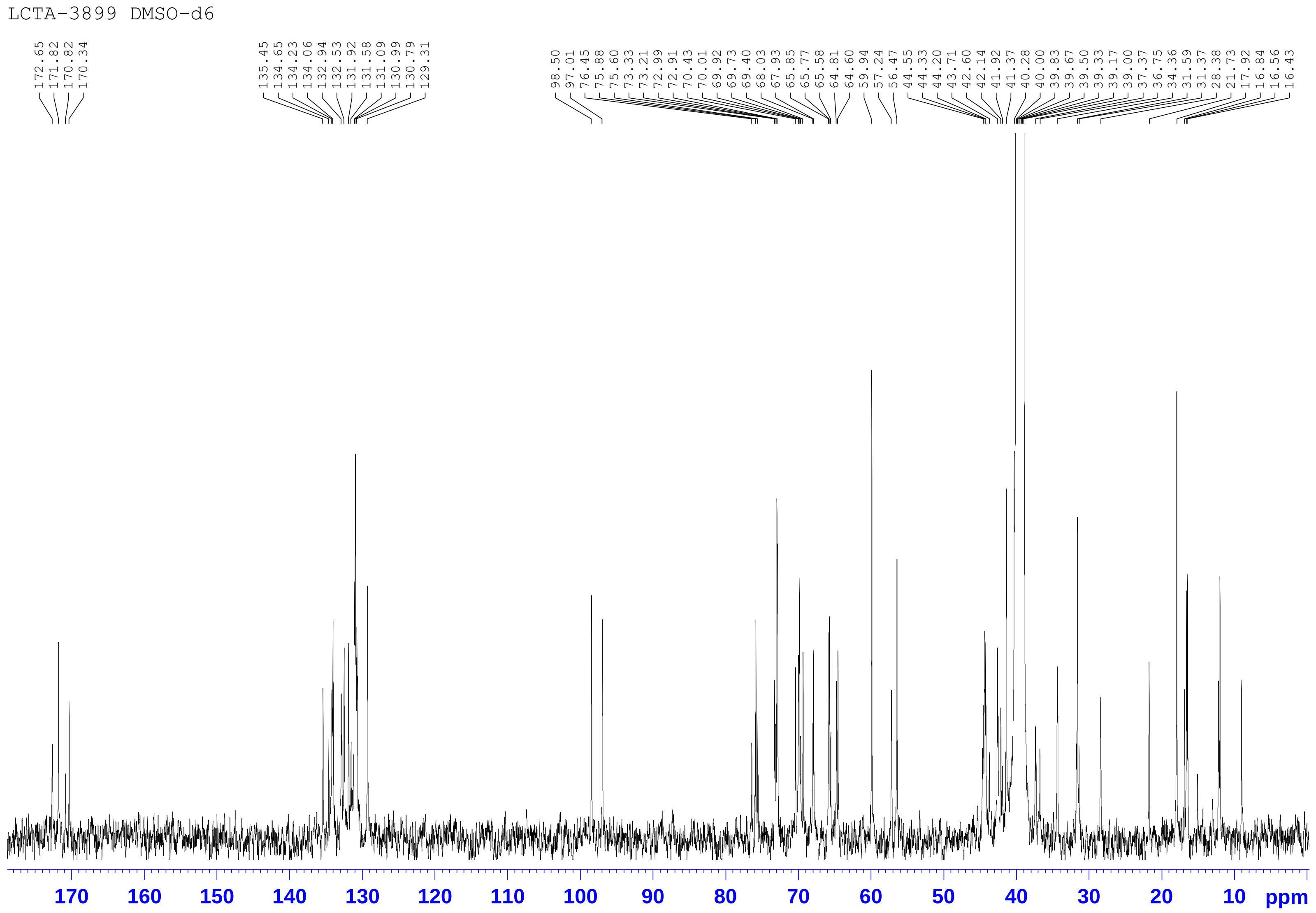

Figure S27. ^{13}C NMR spectra of the Nys derivative **9**.

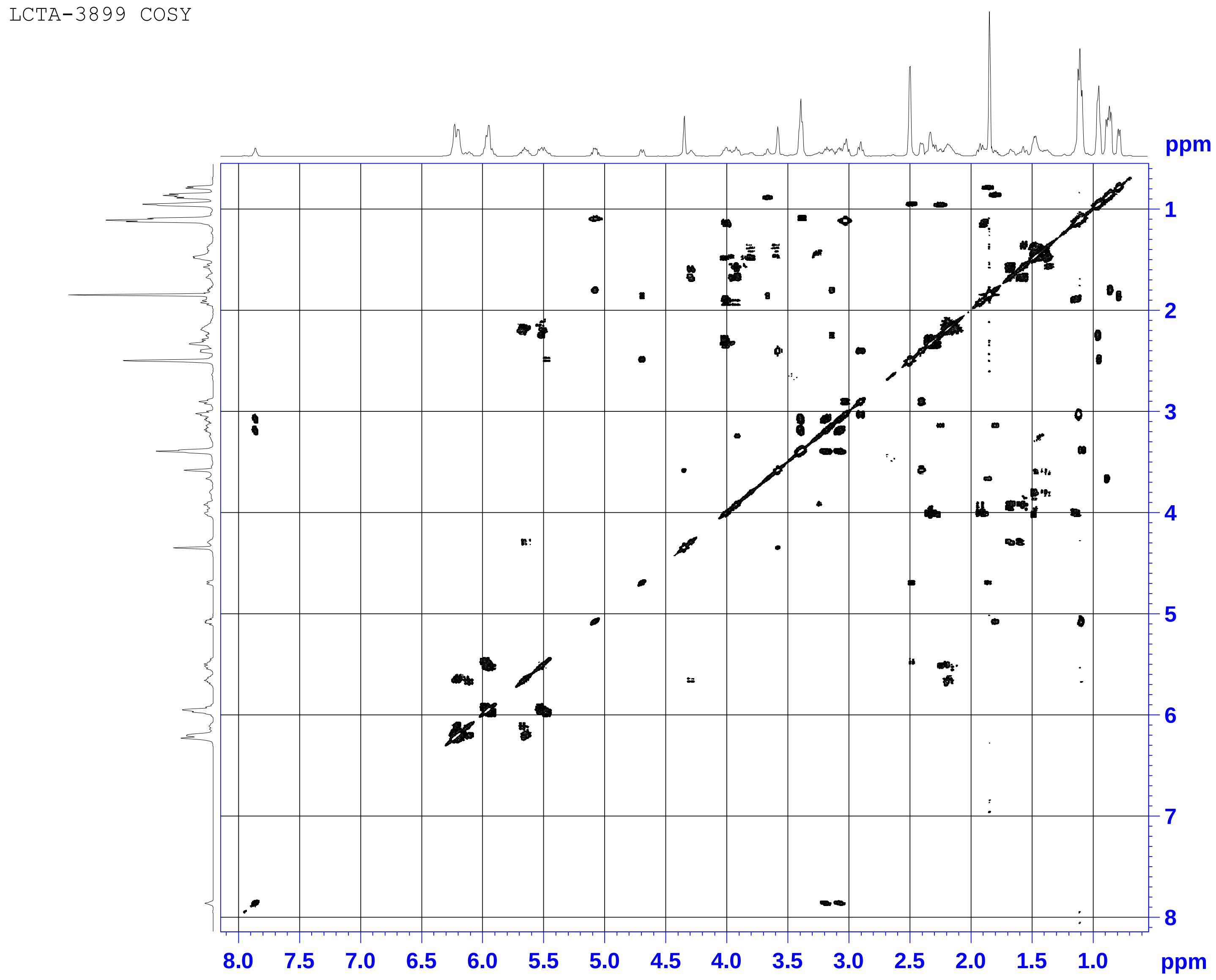

Figure S28. ^{1}H-^{1}H COSY spectra of the Nys derivative **9**.

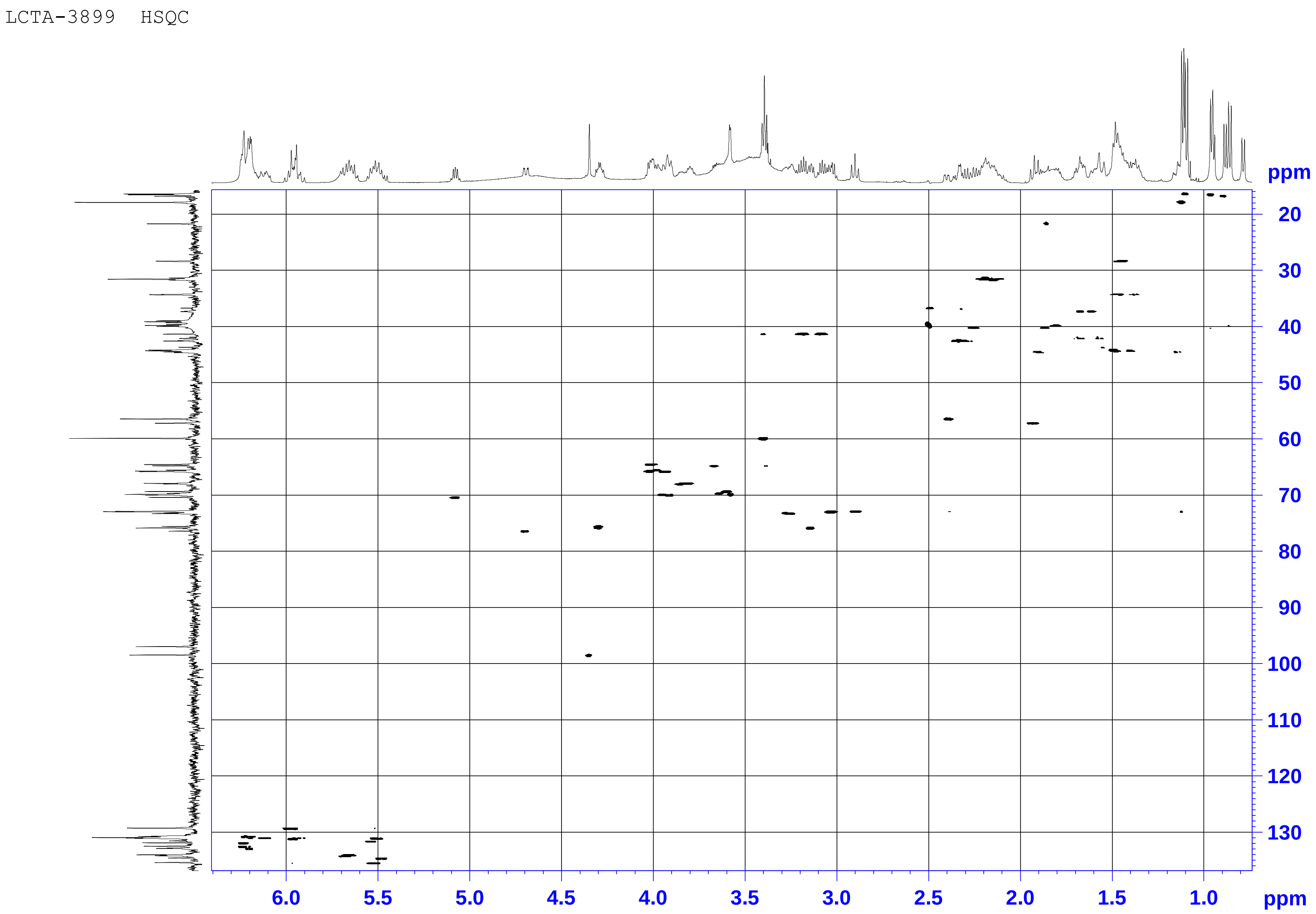

Figure S29. ^{1}H -^{13}C HSQC NMR spectra of the Nys derivative **9**.

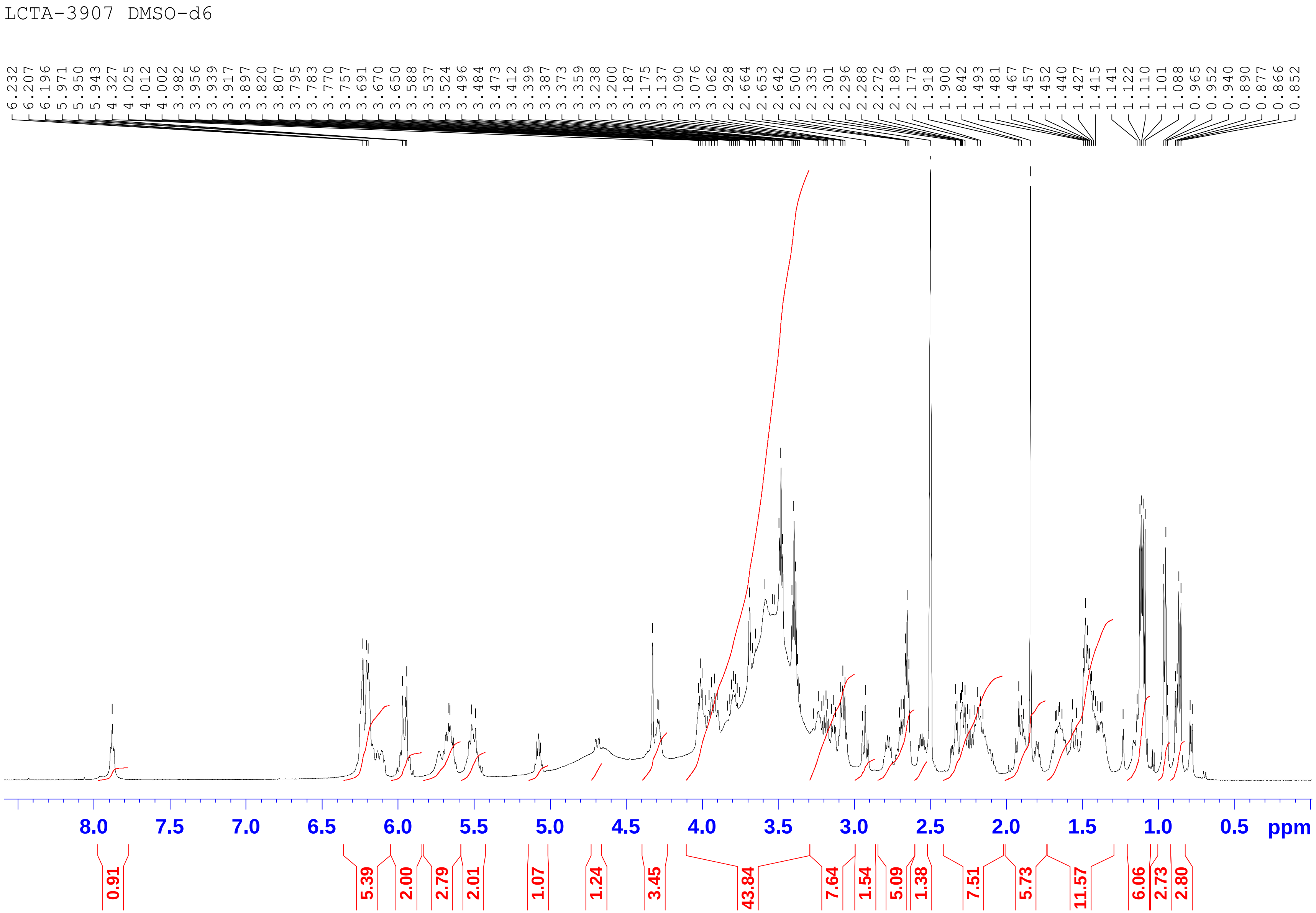

Figure S30. ^{1}H NMR spectra of the Nys derivative **10**.

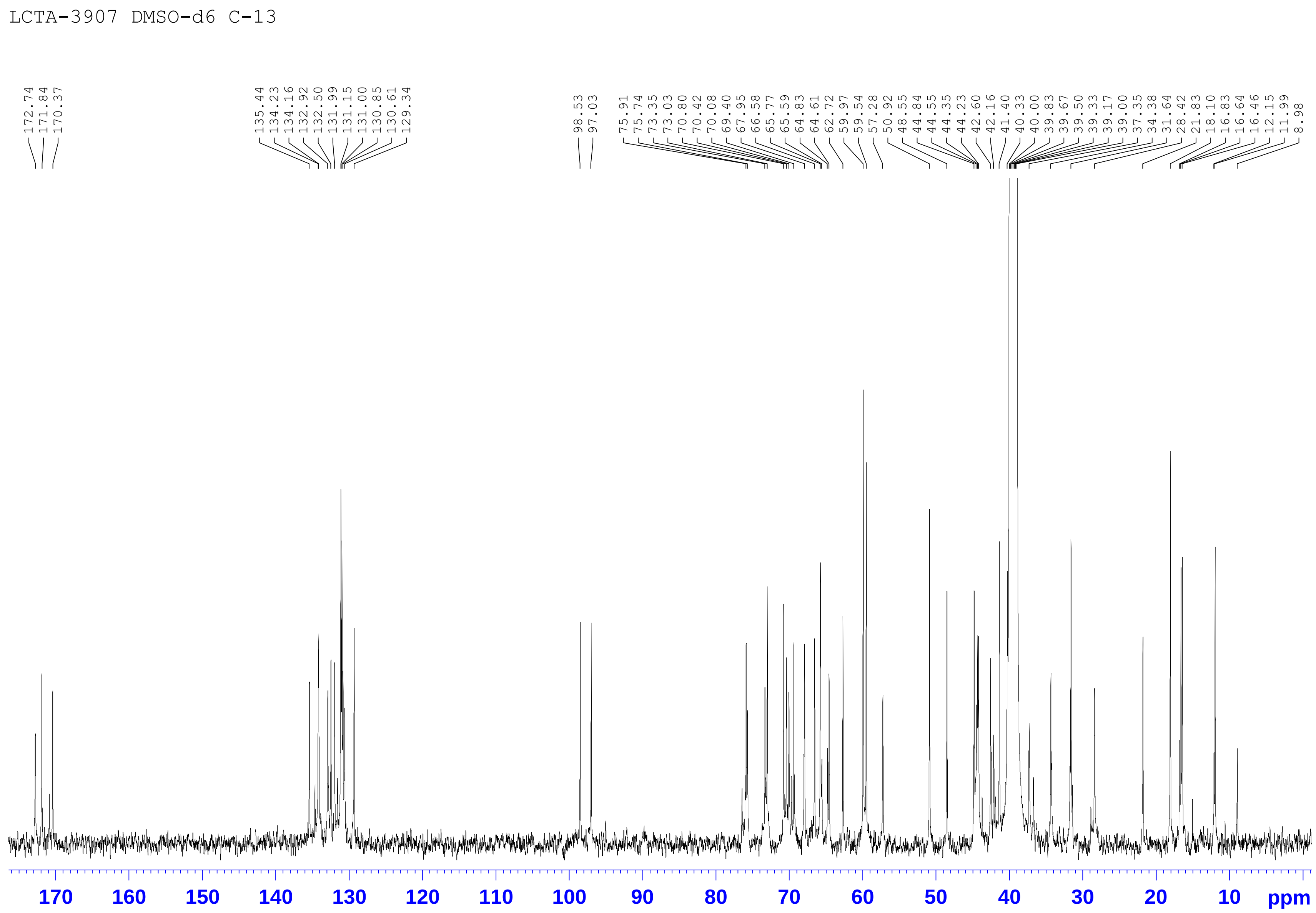

Figure S31. ^{13}C NMR spectra of the Nys derivative **10**.

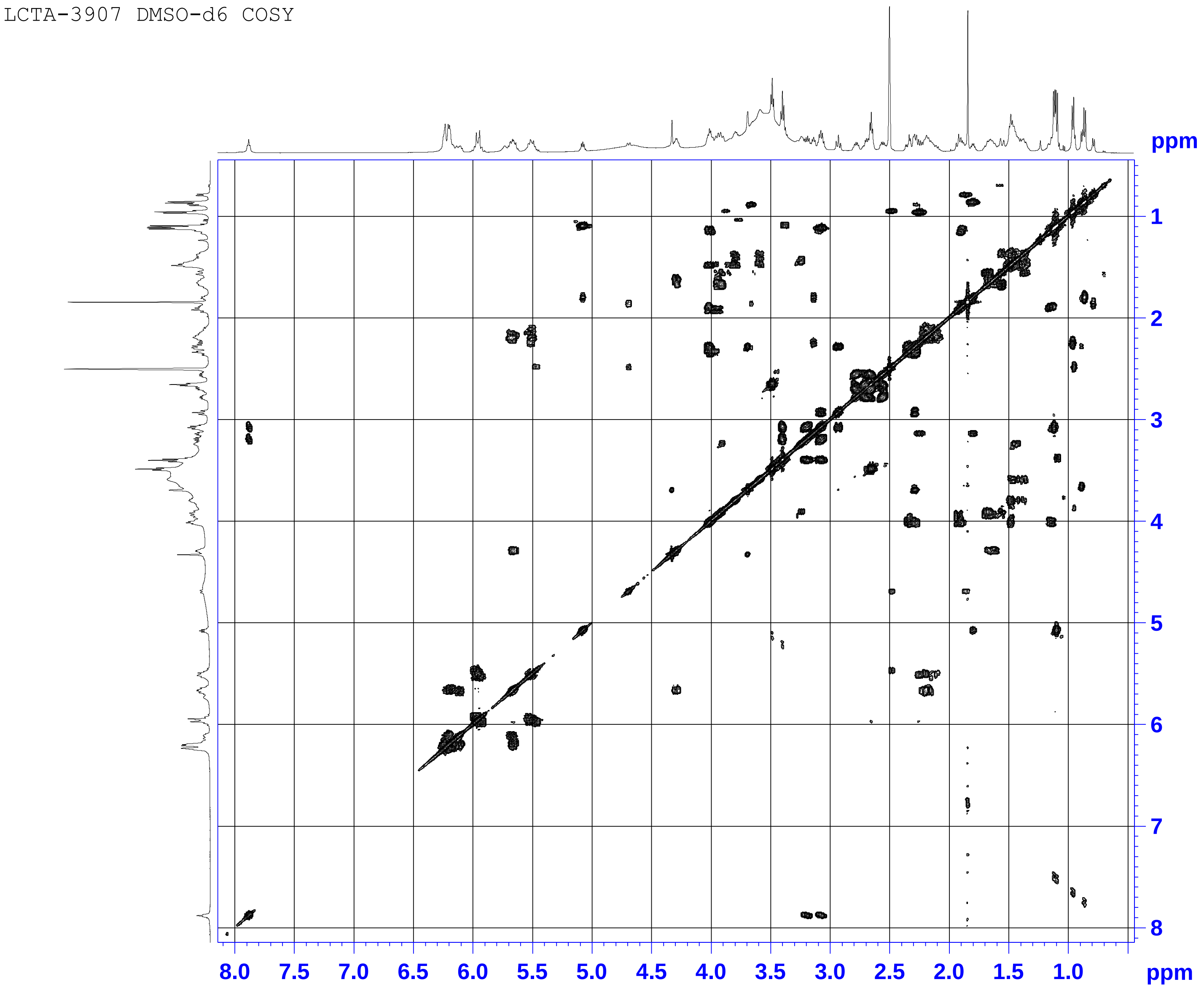

Figure S32. ^{1}H-^{1}H COSY spectra of the Nys derivative **10**.

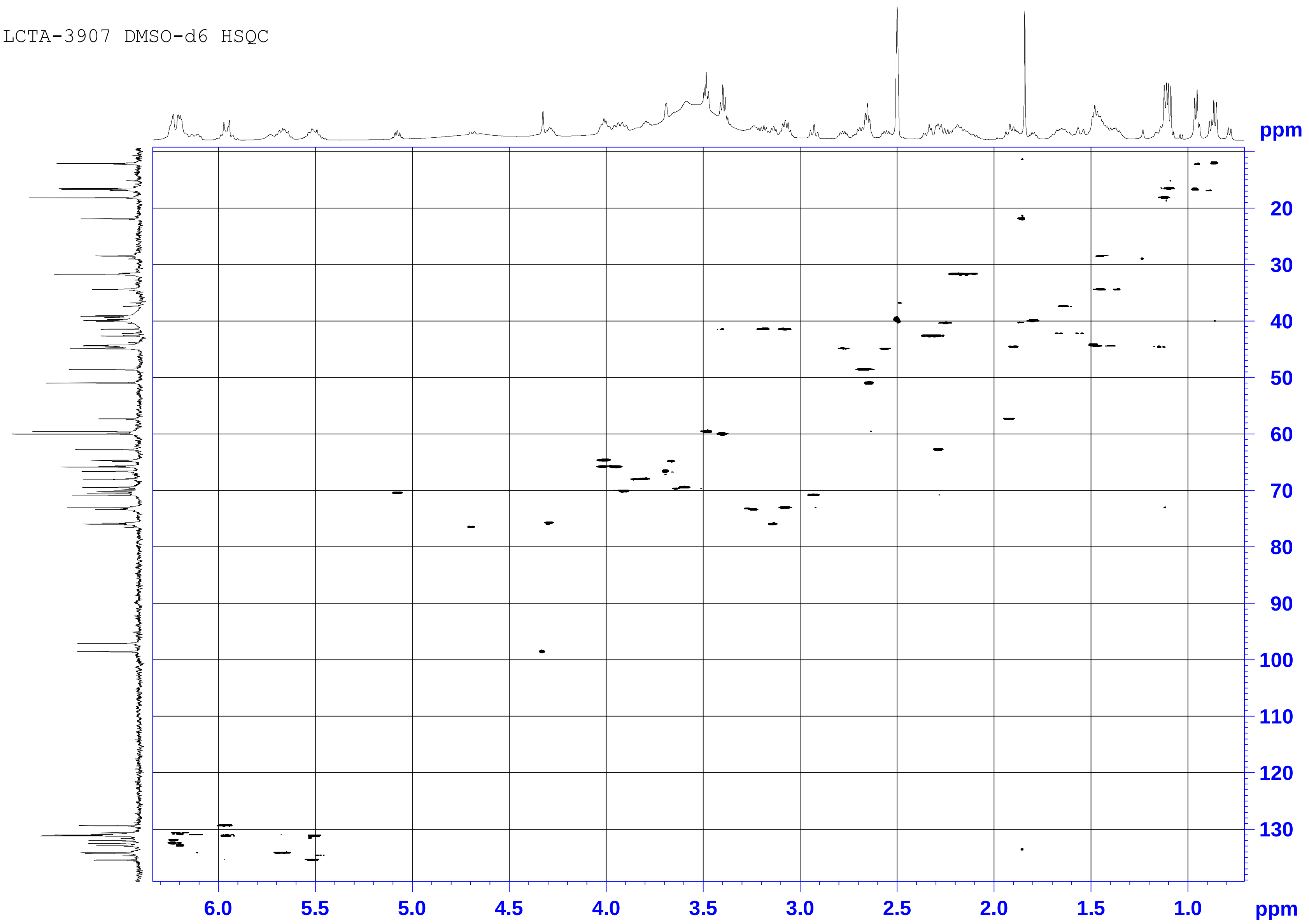

Figure S33. ^{1}H-^{13}C HSQC NMR spectra of the Nys derivative **10**.

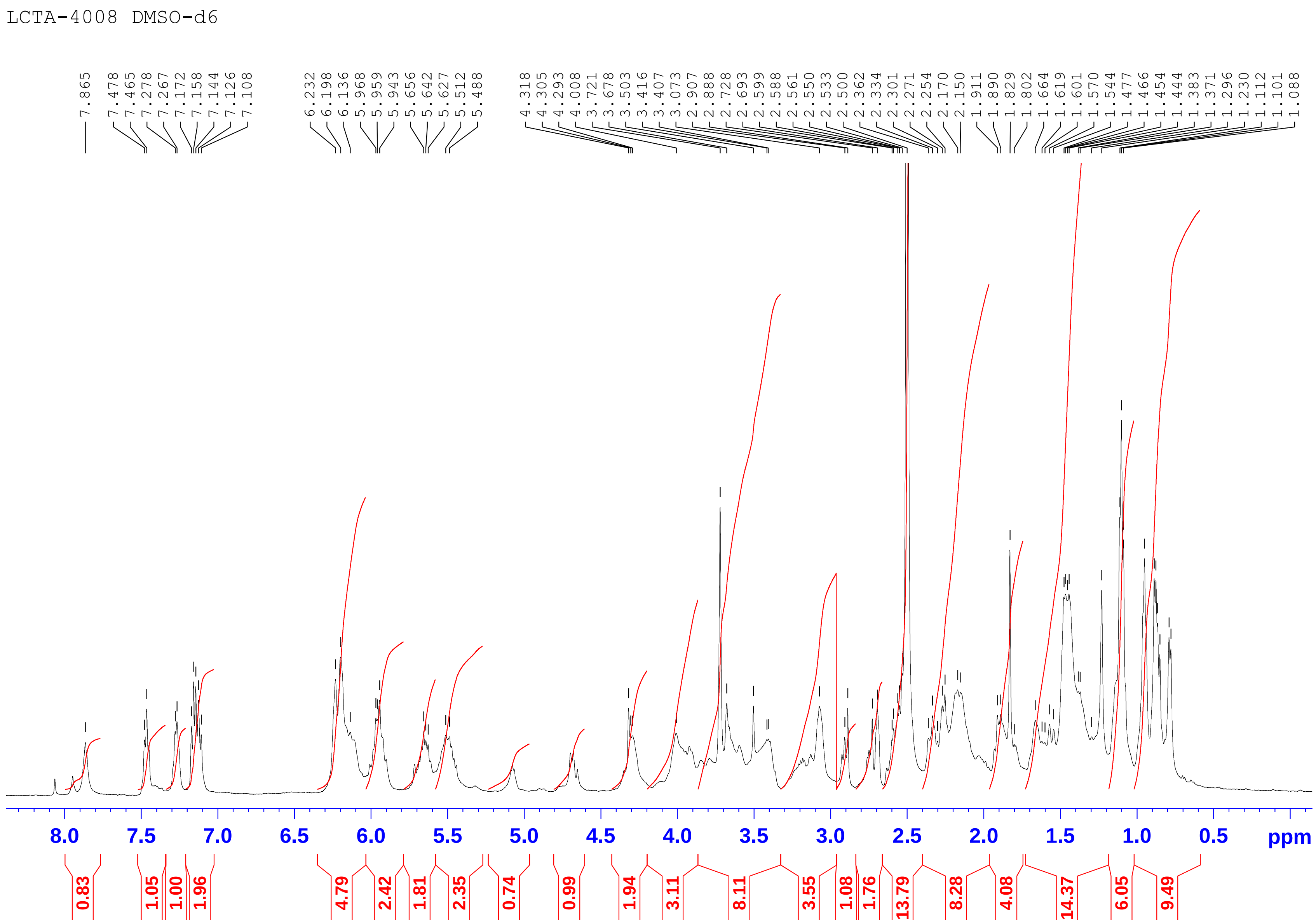

Figure S34. ^{1}H NMR spectra of the Nys derivative **11**.

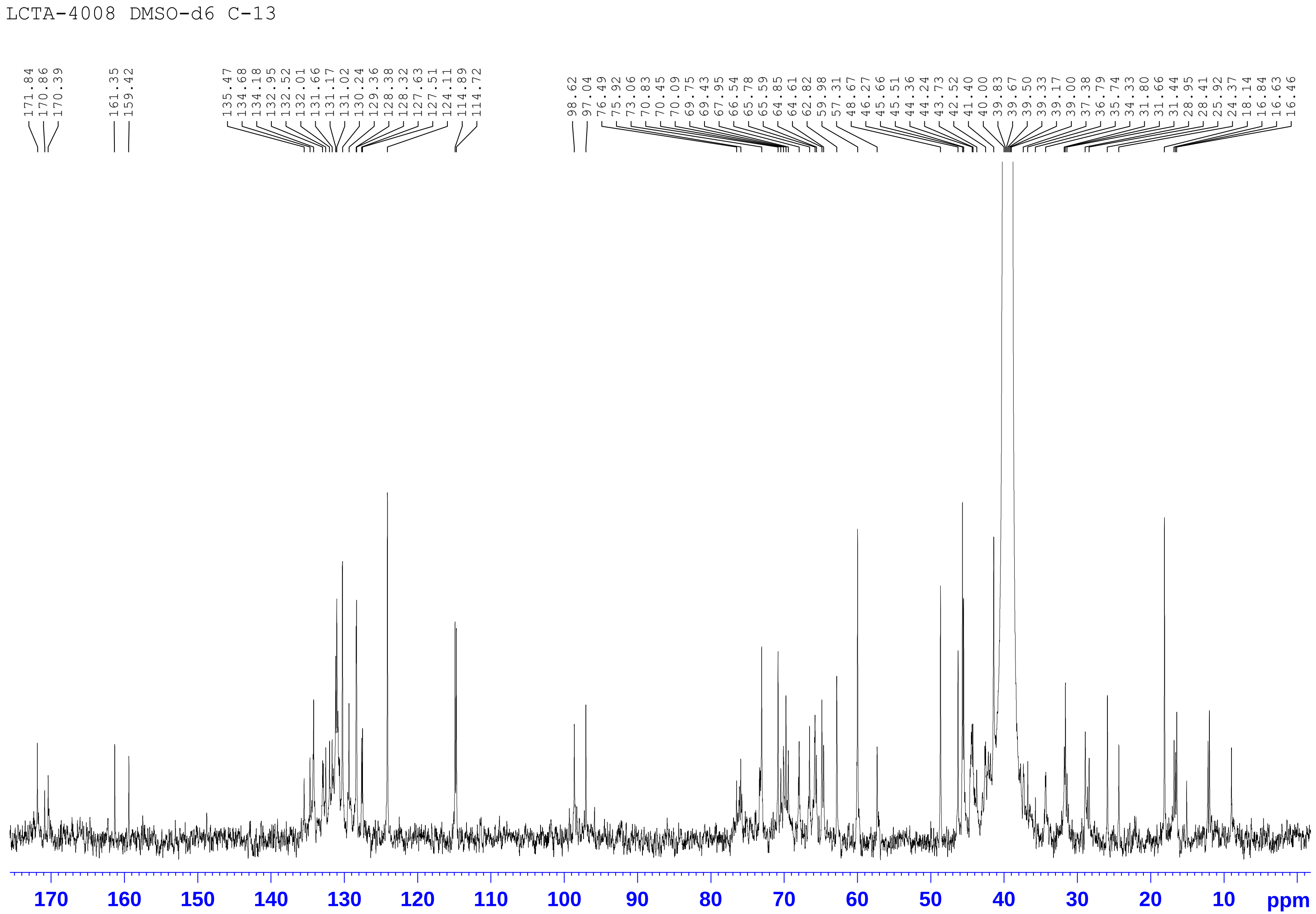

Figure S35. ^{13}C NMR spectra of the Nys derivative **11**.

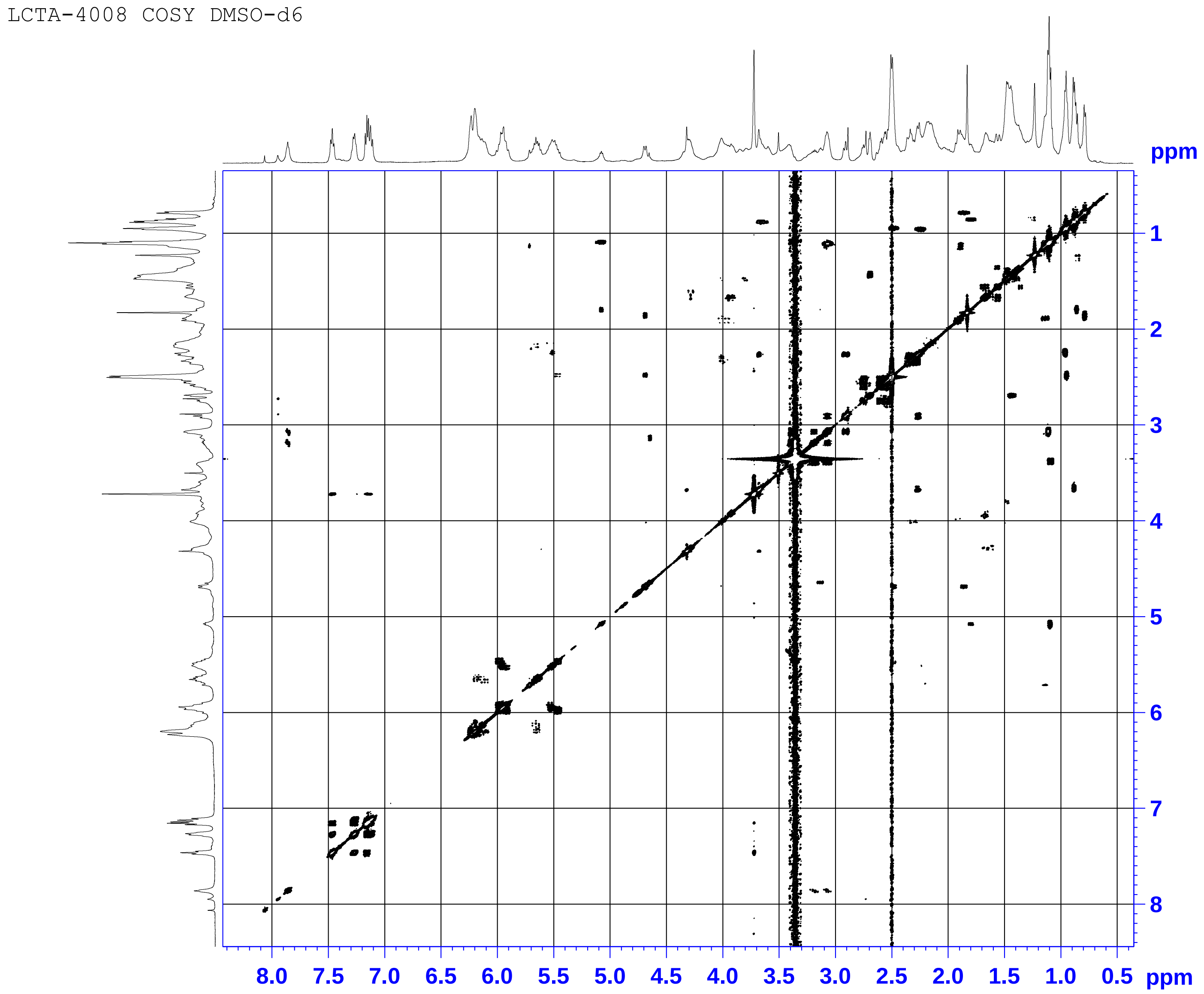

Figure S36. ^{1}H-^{1}H COSY spectra of the Nys derivative **11**.

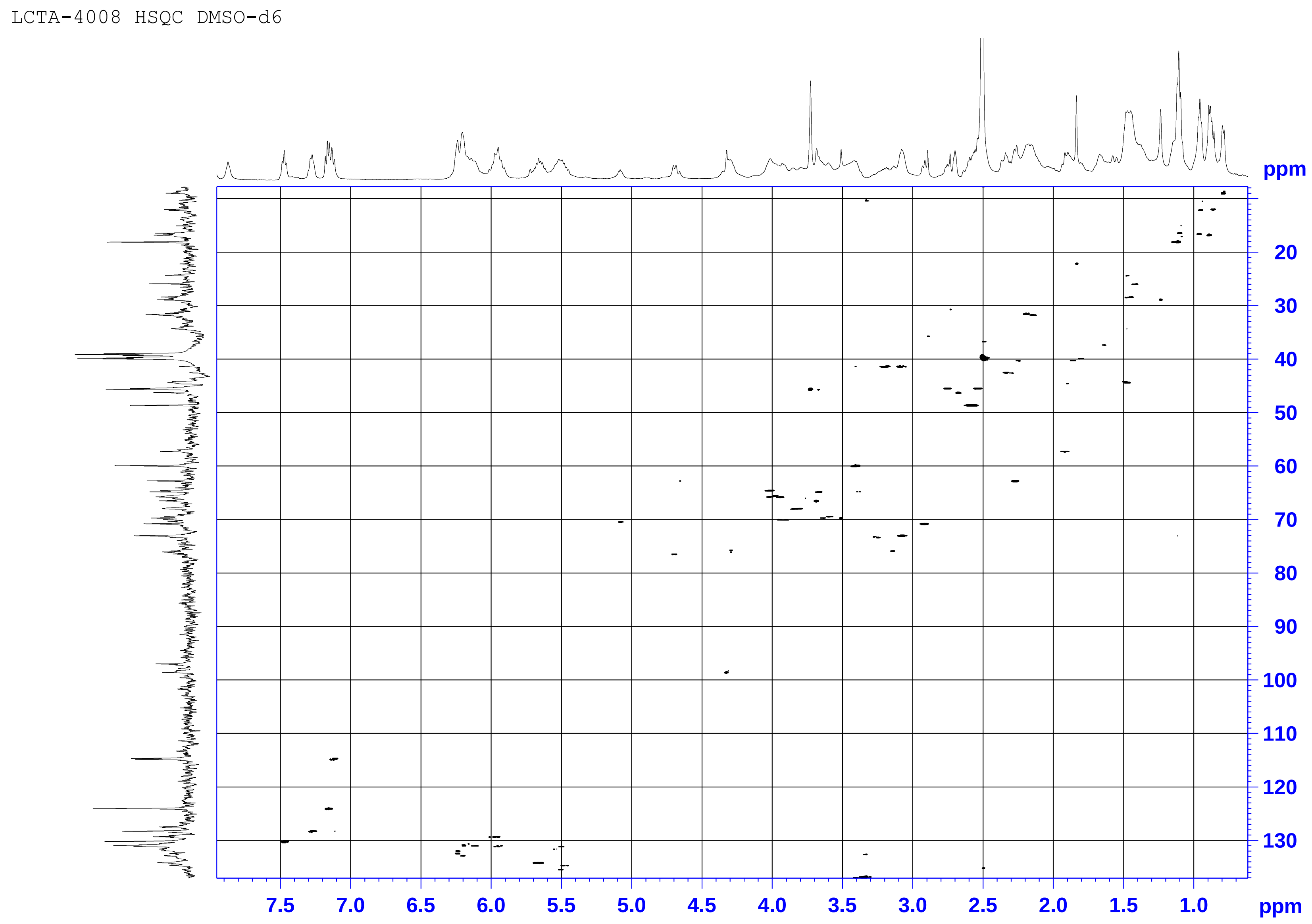

Figure S37. ^{1}H-^{13}C HSQC NMR spectra of the Nys derivative **11**.

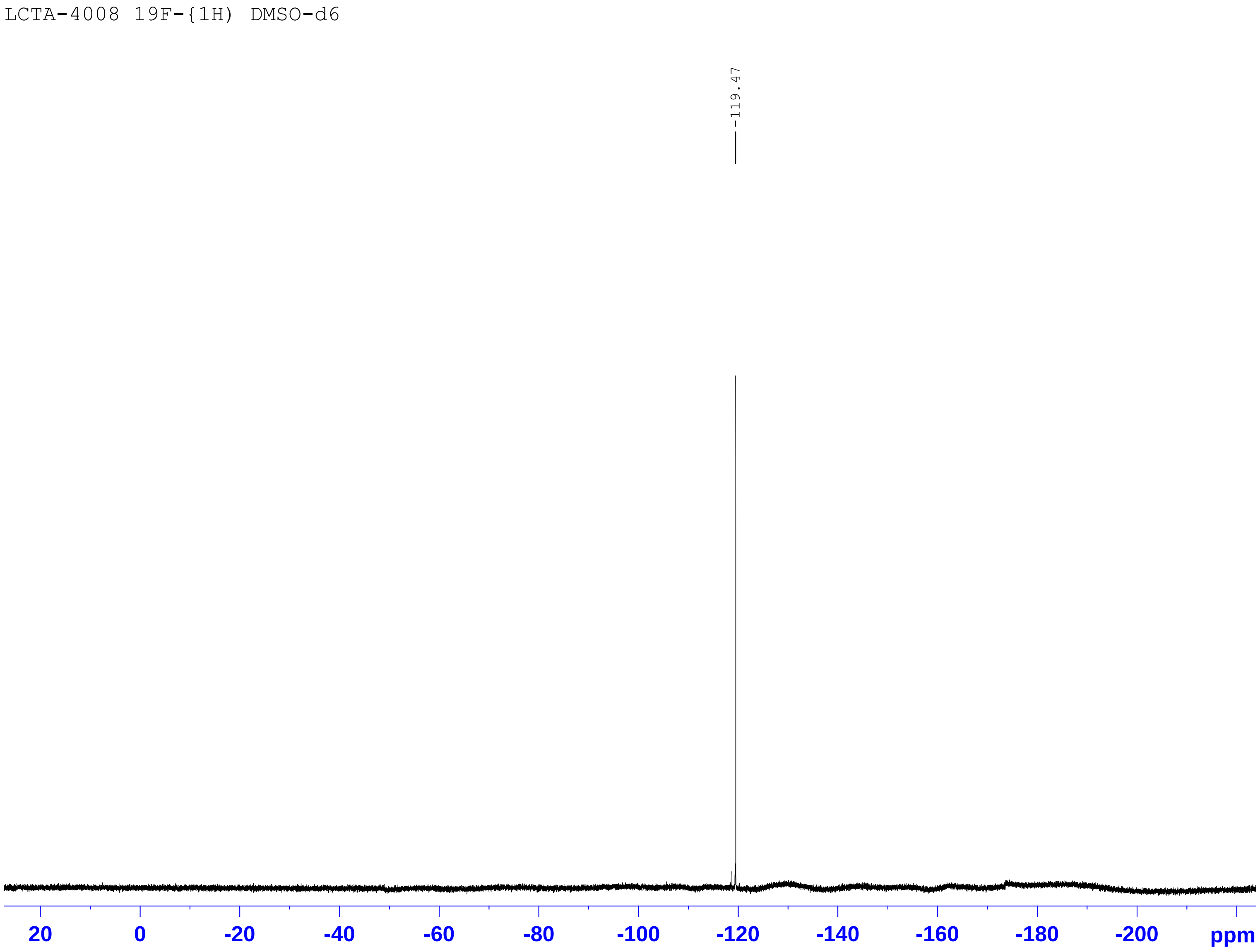

Figure S38. ^{19}F-{1H} NMR spectra of the Nys derivative **11**.

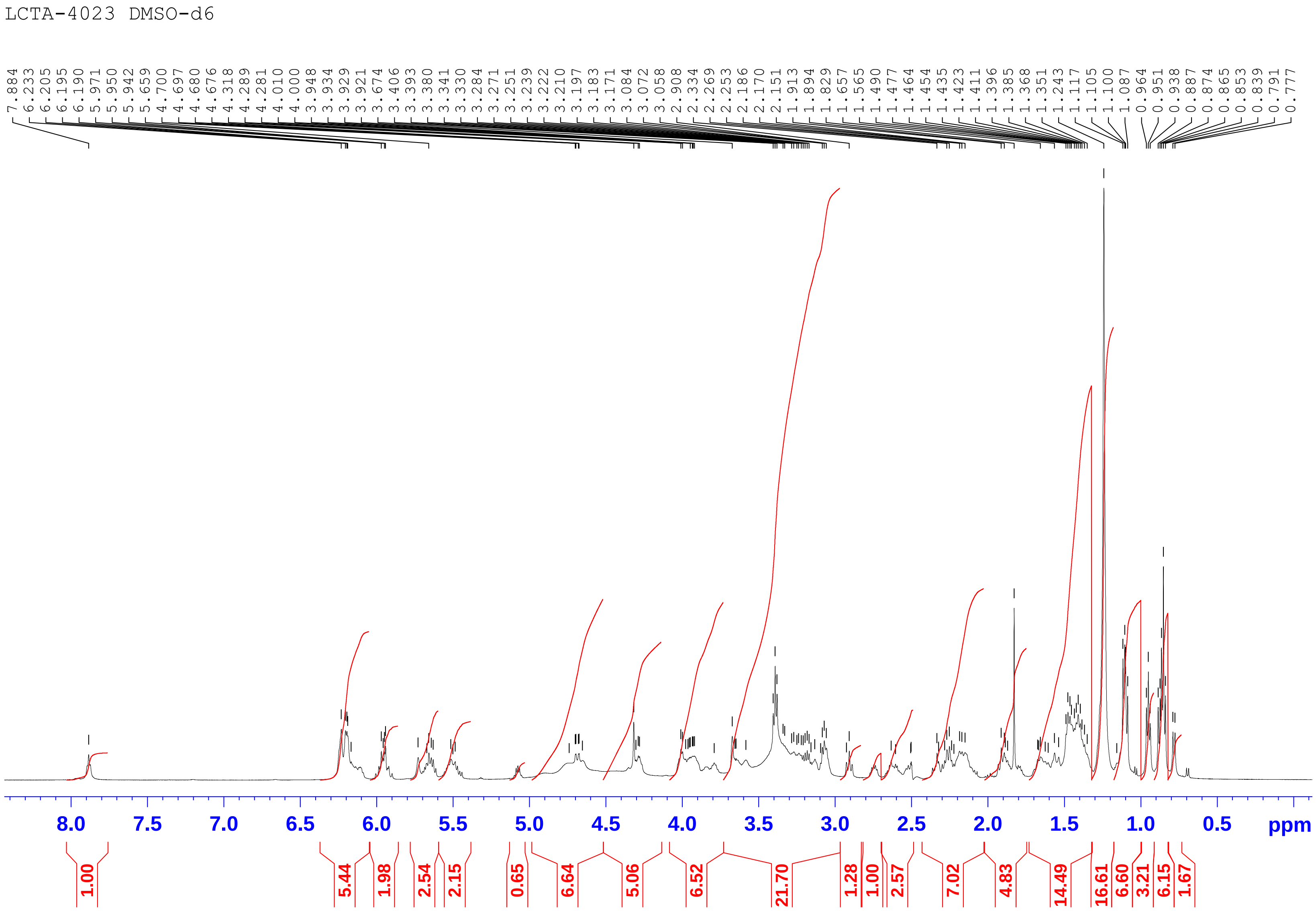

Figure S39. ^{1}H NMR spectra of the Nys derivative **12**.

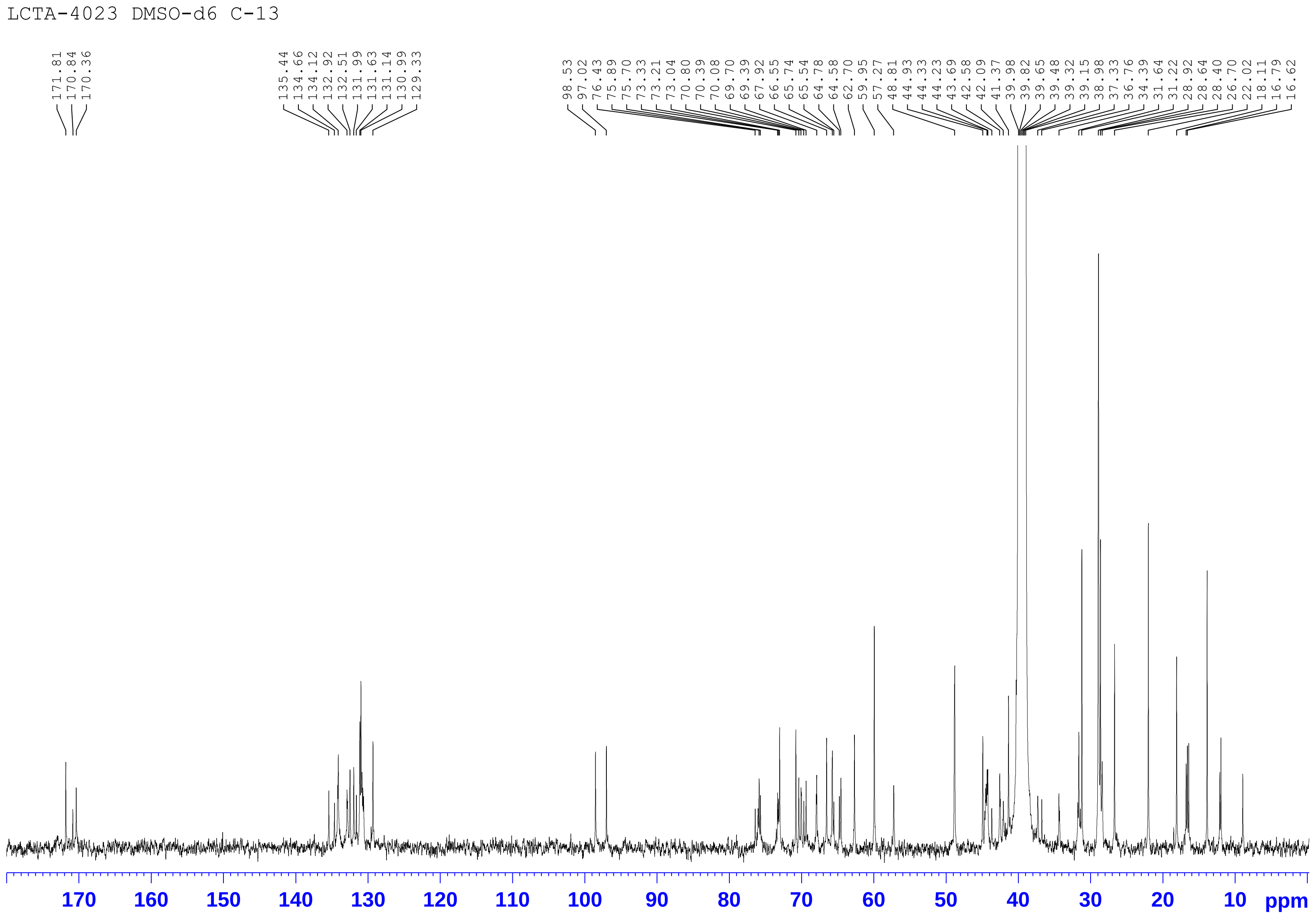

Figure S40. ^{13}C NMR spectra of the Nys derivative **12**.

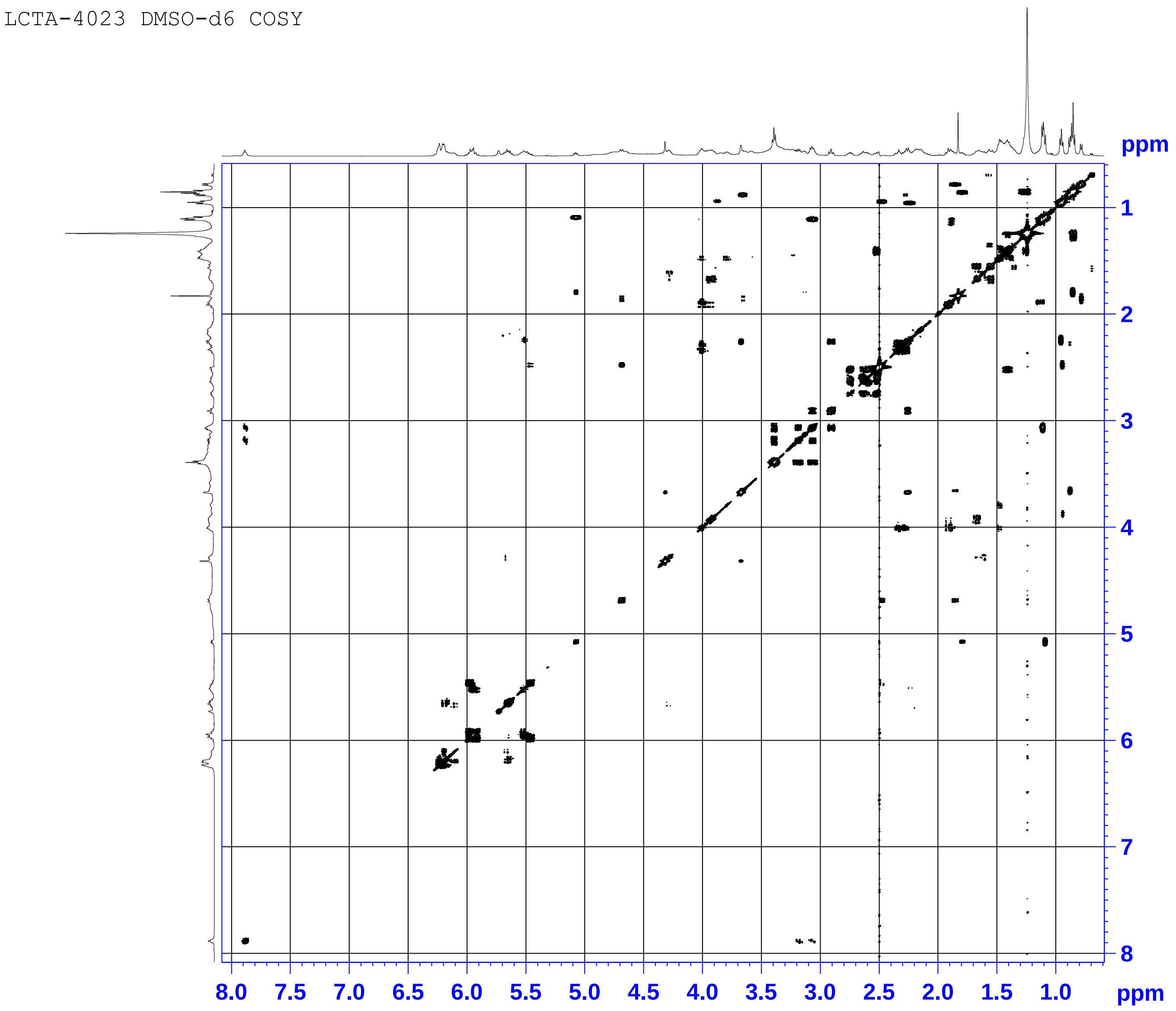

Figure S41. ^{1}H-^{1}H COSY spectra of the Nys derivative **12**.

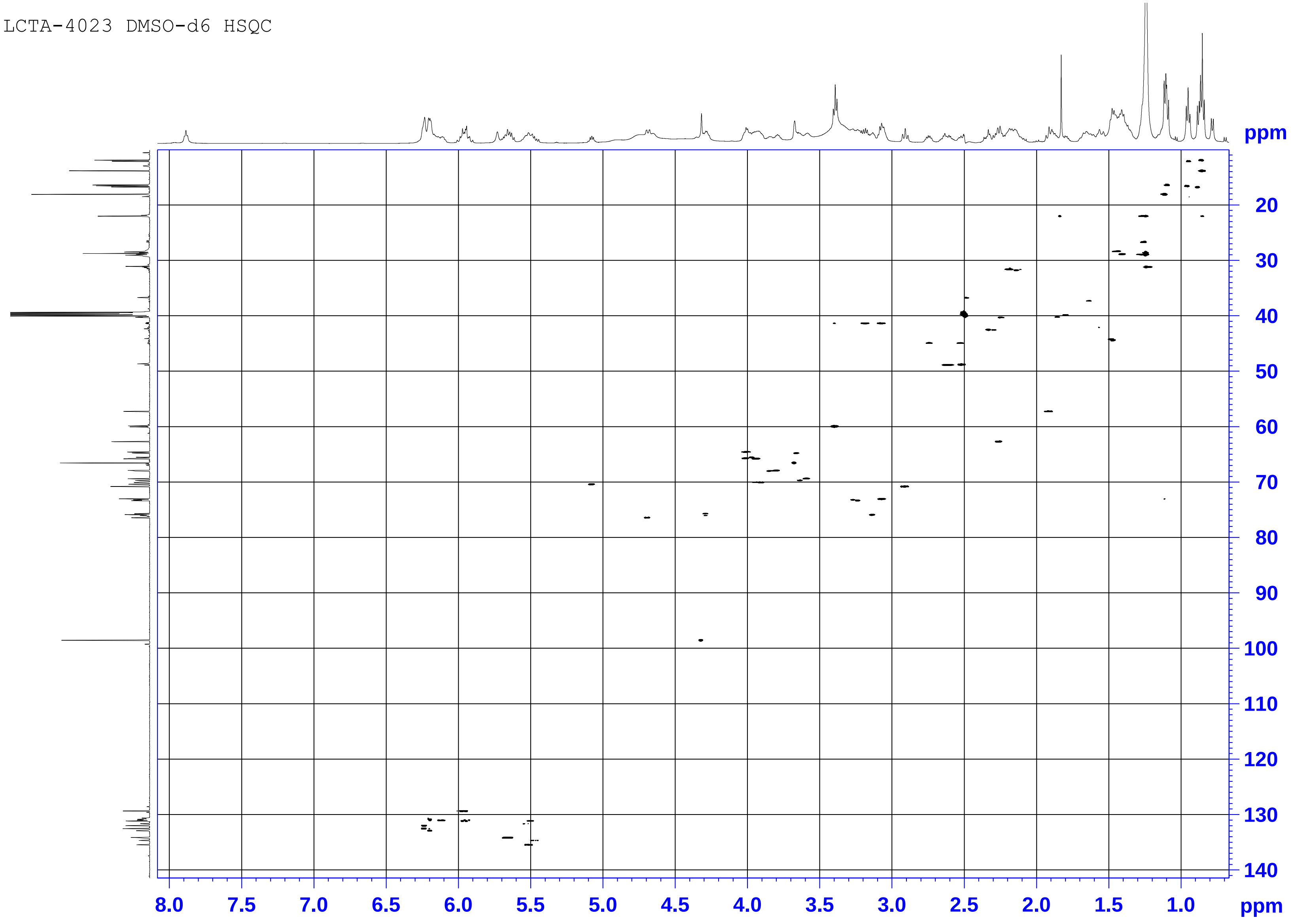

Figure S42. ^{1}H-^{13}C HSQC NMR spectra of the Nys derivative **12**.

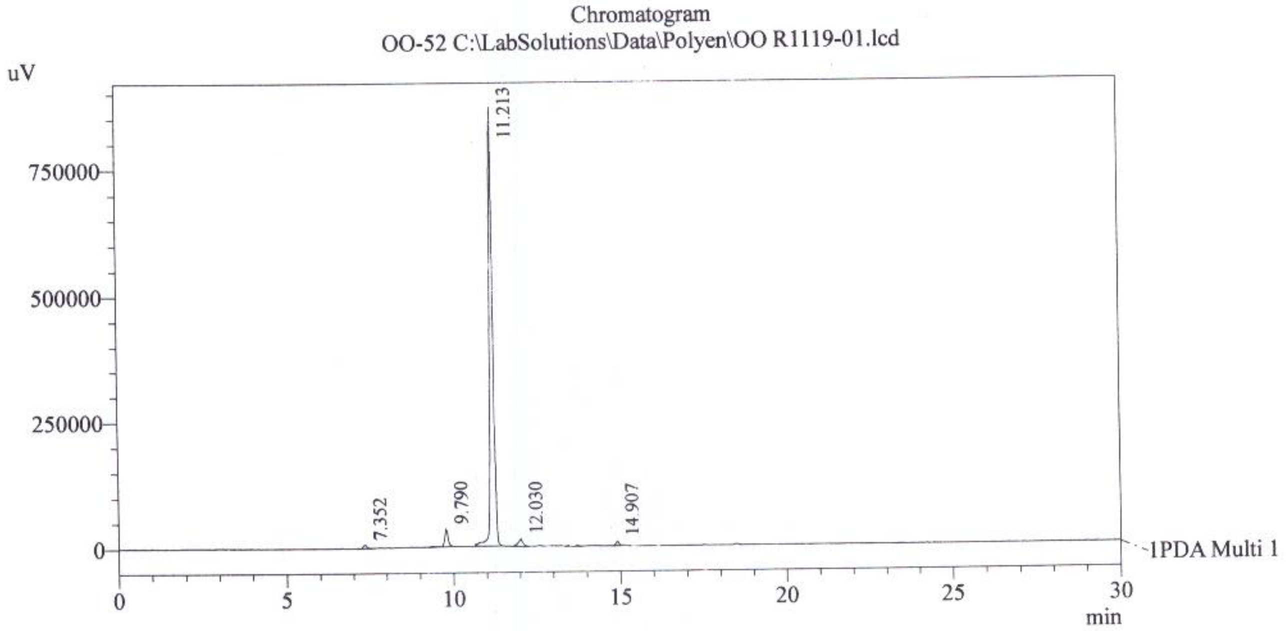

1 PDA Multi 1 / 408nm 4nm

PDA Ch1 408nm 4nm

Peak#	Ret. Time	Area	Height	Area %
1	7.352	51475	6712	0.661
2	9.790	260496	34809	3.344
3	11.213	7275489	907024	93.398
4	12.030	144594	14745	1.856
5	14.907	57750	8895	0.741
Total		7789804	972185	100.000

Figure S43. HPLC chromatogram of the AmB derivative **3**.

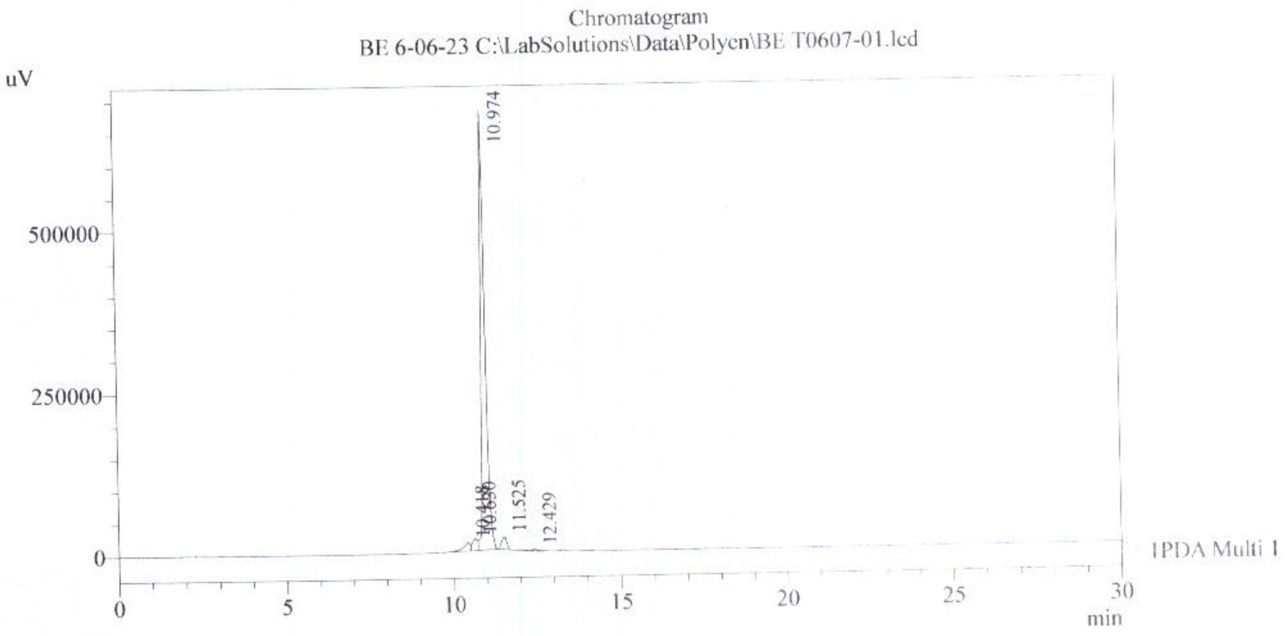

1 PDA Multi 1 / 320nm 4nm

PDA Ch1 320nm 4nm

Peak#	Ret. Time	Area	Height	Area %
1	10.418	164969	12305	2.392
2	10.630	189627	17966	2.749
3	10.974	6306947	684627	91.436
4	11.525	217508	19490	3.153
5	12.429	18604	2103	0.270
Total		6897655	736491	100.000

Figure S44. HPLC chromatogram of the Nys derivative **4**.

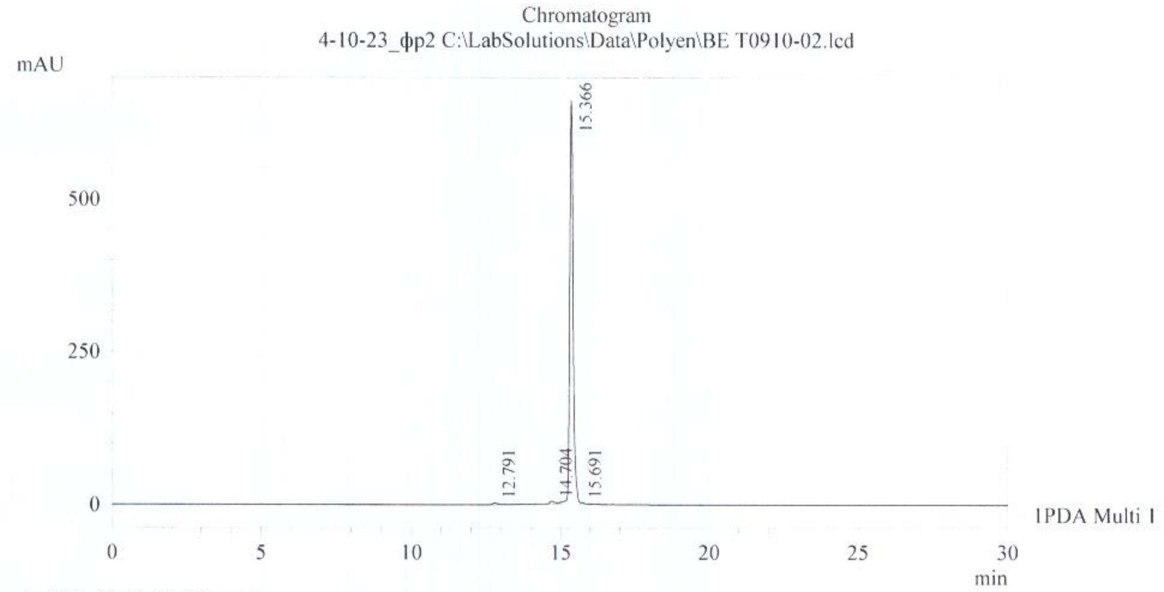

1 PDA Multi 1 / 408nm 4nm

PeakTable

PDA Ch1 408nm 4nm

Peak#	Ret. Time	Area	Height	Area %
1	12.791	30236	2915	0.559
2	14.704	92012	5248	1.701
3	15.366	5238506	660660	96.857
4	15.691	47721	3073	0.882
Total		5408475	671896	100.000

Figure S45. HPLC chromatogram of the AmB derivative **5**.

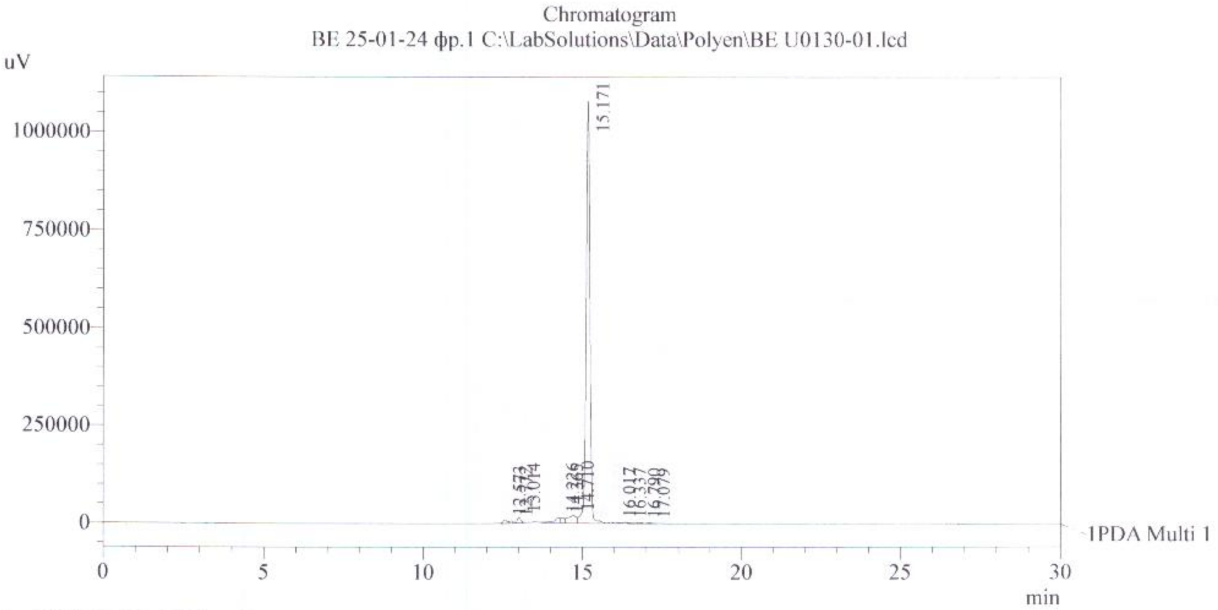

1 PDA Multi 1 / 408nm 4nm

PDA Ch1 408nm 4nm

Peak#	Ret. Time	Area	Height	Area %
1	12.573	73991	8398	0.681
2	12.772	39517	5125	0.364
3	13.014	104822	13586	0.964
4	14.226	170311	13455	1.567
5	14.365	104512	12332	0.962
6	14.710	346712	18464	3.190
7	15.171	9936191	1127622	91.417
8	16.017	28657	2941	0.264
9	16.337	31910	2466	0.294
10	16.790	19940	2080	0.183
11	17.079	12576	1478	0.116
Total		10869140	1207948	100.000

Figure S46. HPLC chromatogram of the AmB derivative **6**.

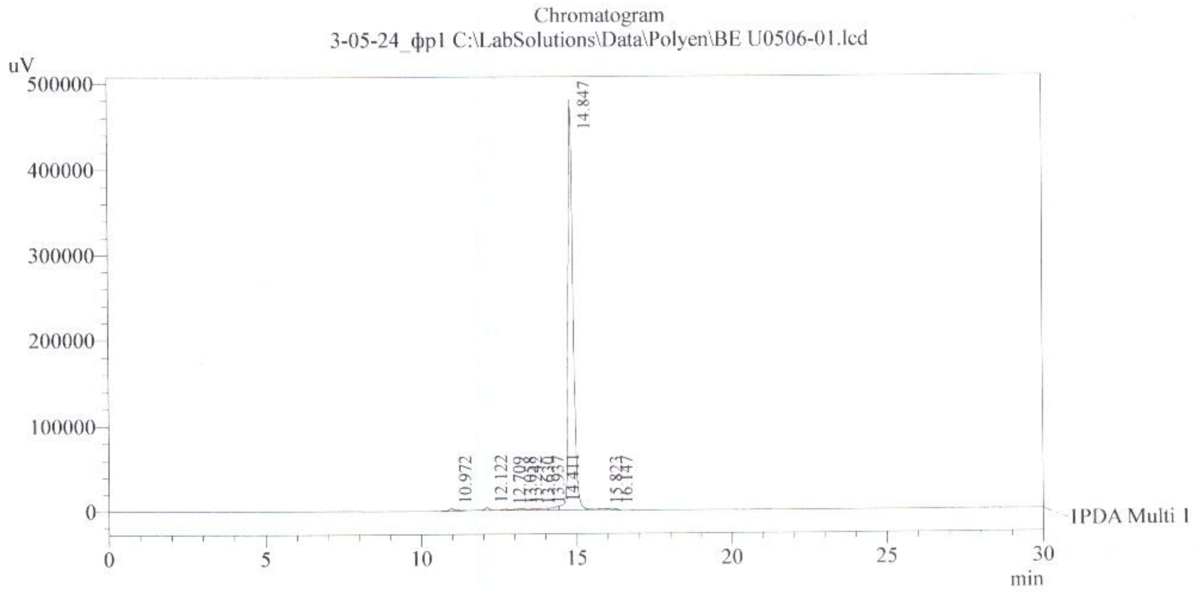

1 PDA Multi 1 / 408nm 4nm

PDA Ch1 408nm 4nm

Peak#	Ret. Time	Area	Height	Area %
1	10.972	43055	3029	0.752
2	12.122	29931	3169	0.523
3	12.709	8467	908	0.148
4	13.058	10126	963	0.177
5	13.242	17589	1469	0.307
6	13.630	13795	977	0.241
7	13.937	28019	1715	0.489
8	14.411	52922	3977	0.924
9	14.847	5481346	479859	95.727
10	15.823	16552	1255	0.289
11	16.147	24190	1418	0.422
Total		5725993	498741	100.000

Figure S47. HPLC chromatogram of the AmB derivative **7**.

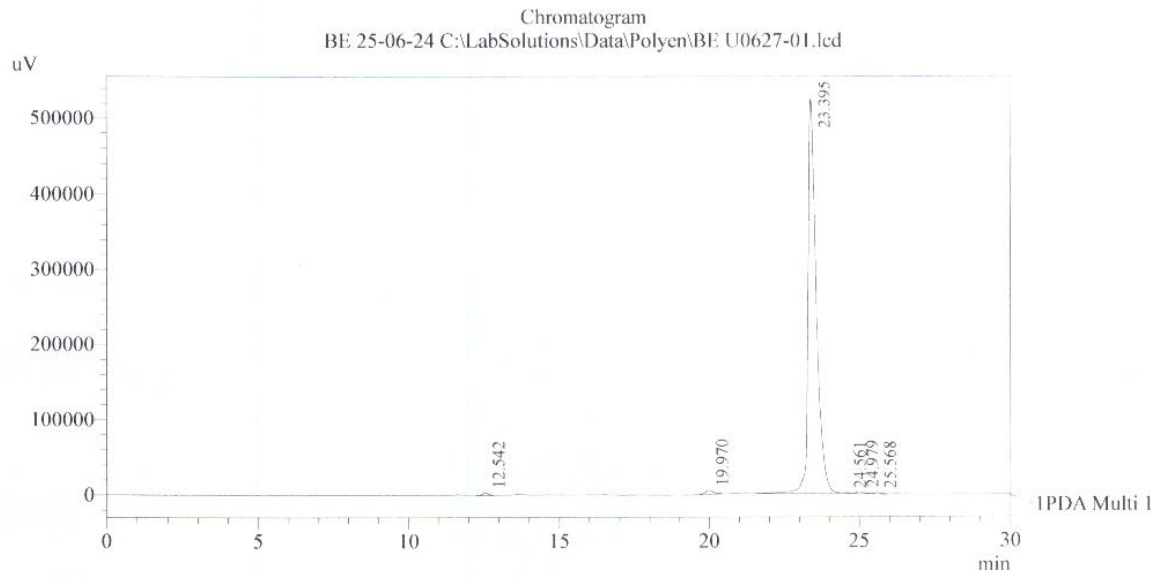

1 PDA Multi 1 / 408nm 4nm

PDA Ch1 408nm 4nm

Peak#	Ret. Time	Area	Height	Area %
1	12.542	38436	3267	0.362
2	19.970	100646	5171	0.948
3	23.395	10421909	524378	98.136
4	24.561	4743	507	0.045
5	24.979	33571	2266	0.316
6	25.568	20605	1387	0.194
Total		10619910	536975	100.000

Figure S48. HPLC chromatogram of the AmB derivative **8**.

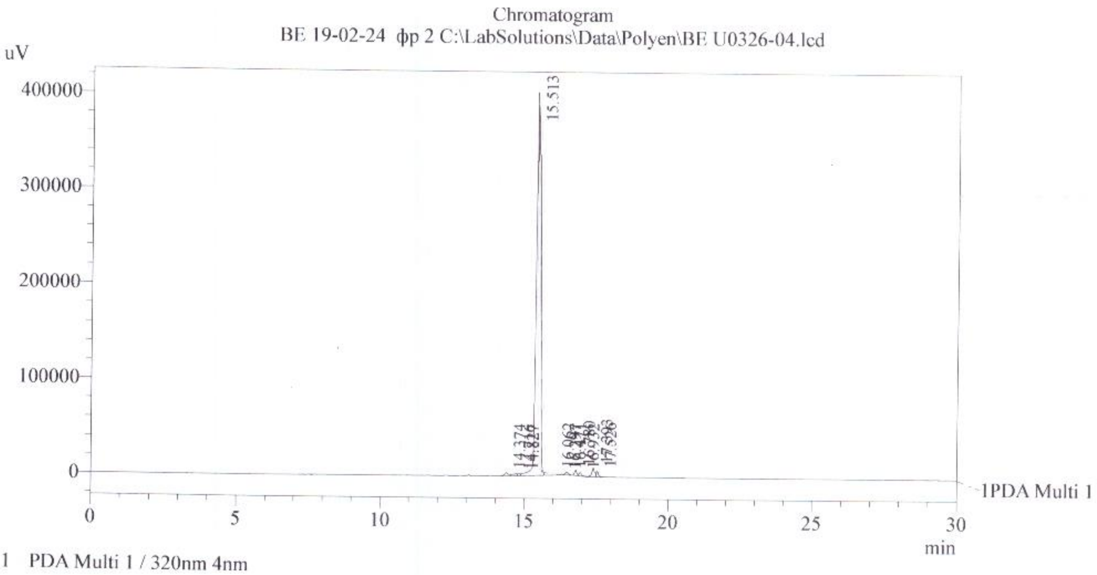

1 PDA Multi 1 / 320nm 4nm

PDA Ch1 320nm 4nm

Peak#	Ret. Time	Area	Height	Area %
1	14.374	33389	3321	0.775
2	14.716	25454	2077	0.591
3	14.827	14840	2176	0.345
4	15.513	4000049	677446	92.900
5	16.062	48008	3189	1.115
6	16.297	9623	1807	0.223
7	16.471	21666	2991	0.503
8	16.780	33320	5783	0.774
9	16.932	19188	3510	0.446
10	17.393	67290	9422	1.563
11	17.526	32922	5752	0.765
Total		4305750	717473	100.000

Figure S49. HPLC chromatogram of the Nys derivative **9**.

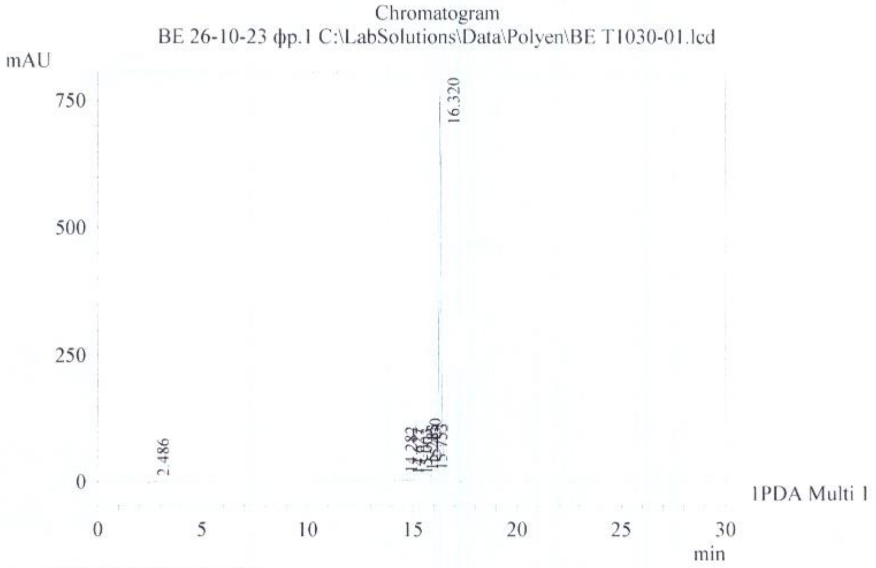

1 PDA Multi 1 / 320nm 4nm

PeakTable

PDA Ch1 320nm 4nm

Peak#	Ret. Time	Area	Height	Area %
1	2.486	48741	4979	0.532
2	14.282	46746	6327	0.510
3	14.627	12944	2094	0.141
4	15.003	26426	3088	0.288
5	15.283	96164	10960	1.049
6	15.430	278074	23004	3.034
7	15.753	120926	12052	1.319
8	16.320	6709705	759688	93.126
Total		9165033	1029704	100.000

Figure S50. HPLC chromatogram of the AmB derivative **10**.

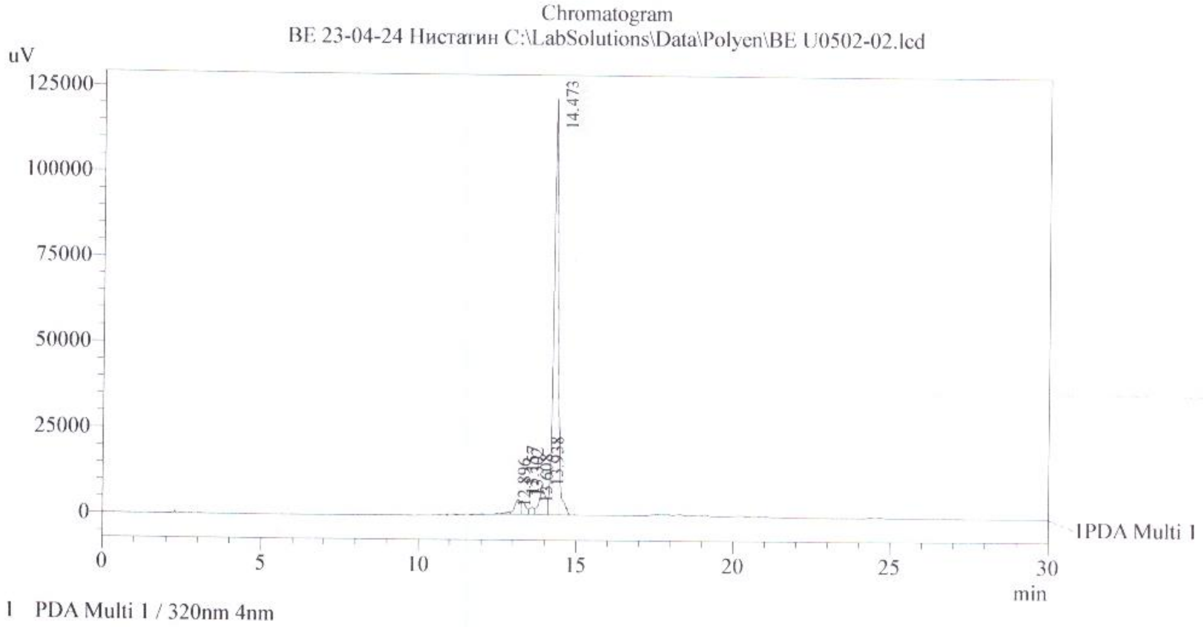

PDA Ch1 320nm 4nm

Peak#	Ret. Time	Area	Height	Area %
1	12.896	7143	697	0.241
2	13.157	54137	4271	1.823
3	13.302	39670	3824	1.336
4	13.608	21073	1986	0.710
5	13.938	114276	7026	3.849
6	14.473	2732706	251553	92.041
Total		2969005	269359	100.000

Figure S51. HPLC chromatogram of the AmB derivative **11**.

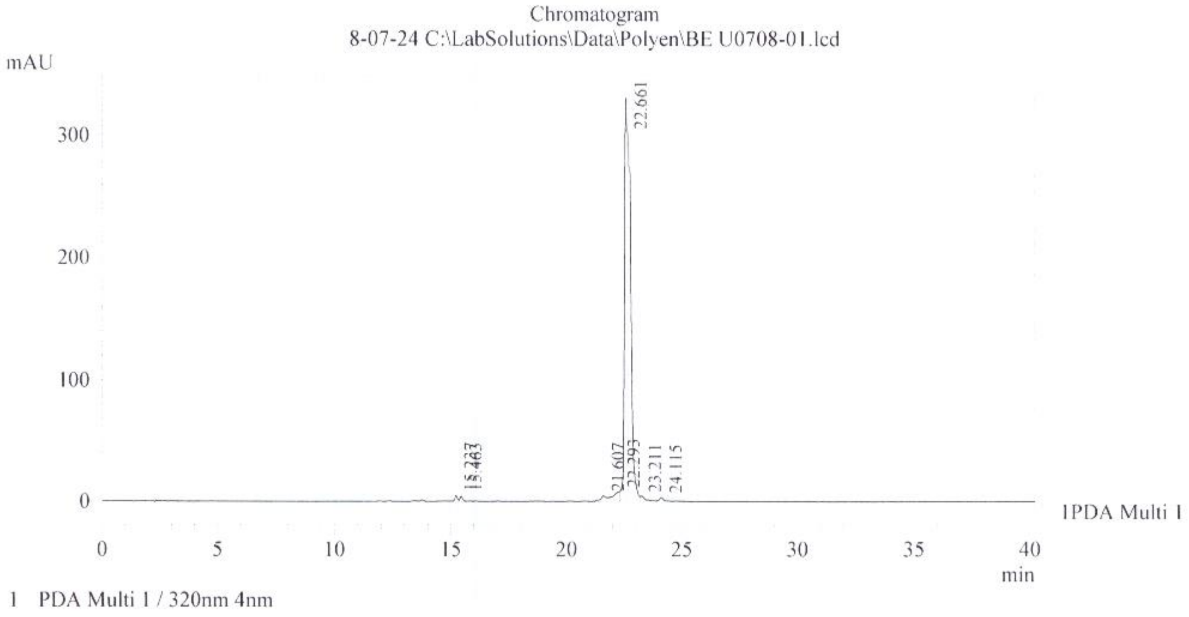

PeakTable

PDA Ch1 320nm 4nm

Peak#	Ret. Time	Area	Height	Area %
1	15.237	36946	4700	0.575
2	15.463	33602	4255	0.523
3	21.607	77009	4423	1.199
4	22.293	142867	7512	2.224
5	22.661	6053761	655444	94.228
6	23.211	53227	3327	0.828
7	24.115	27115	2607	0.422
Total		6424528	682268	100.000

Figure S52. HPLC chromatogram of the AmB derivative **12**.

Synthesis of 2-substituted (9H-fluoren-9-yl)methyl (3-oxopropyl)carbamates

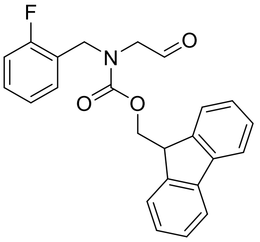

(9H-fluorene-9-yl)methyl-N-(2-fluorobenzyl)-N-(2-oxoethyl)carbamate: 2-((2-Fluorobenzyl)amino)ethanol 1.08 g (0.006 mol) was dissolved in 15 mL of MeCN, then triethylamine (0.61 g, 0.006 mol) was added and the reaction mixture was cooled to 0°C. Then Fmoc-OSu 2.30 g (0.006 mol) was added to the reaction mixture while maintaining cooling and stirring for 30 minutes. Afterward, the cooling source was removed and reaction was allowed to stir at room temperature for 3 hours. Then the solvent was removed using a rotary evaporator. The product was extracted with ethyl acetate (2 x 80 mL), then washed with 0.5% $NaHCO_3$ solution (40 mL) and water (2 x 40 mL). Organic layer was dried over anhydrous sodium sulfate and the solvent was removed using a rotary evaporator. The crude product was dissolved in 20 mL of methylene chloride and Dess-Martin periodinane 5.35 g (0.012 mol) was added to this solution. The reaction mixture was stirred at room temperature for 40 minutes. Then, 20 mL of chloroform and a pre-prepared solution of 9.88 g (0.060 mol) of sodium thiosulfate in 20 mL of saturated $NaHCO_3$ solution were added to the reaction and resulted mixture was stirred vigorously for 10 minutes. The product was extracted with chloroform (2 x 60 mL) and washed with water (3 x 30 mL). The organic layer was dried over anhydrous sodium sulfate and the solvent was removed using a rotary evaporator. The product was purified by column chromatography on silica gel using a petroleum ether:ethyl acetate system with a gradient from 3:1 to 2:1. The pure fractions were combined and evaporated, then the product was dried in a vacuum desiccator for 24 hours. Yield: 1.92 g (83%), a light yellow oil. NMR ^{1}H, (400 MHz, $CDCl_3$), δ, ppm. (*J*, Hz): 9.47 (1H, br s); 7.78-7.75 (2H, m); 7.56-7.51 (2H, m); 7.43-7.39 (2H, m); 7.34-7.25 (3H, m); 7.13-7.10 (1H, m); 7.06-6.99 (1H, m); 6.84-6.81 (1H, m); 4.63--4.20 (5H, m); 4.04 (1H, s); 3.77 (1H, s). NMR ^{13}C (100 MHz, $CDCl_3$), δ, ppm.: 197.79; 162.01, $J_{C\text{-}F}$ = 246.1; 156.38; 143.71; 141.38; 130.93, 130.13; 129.70; 127.76; 127.16; 124.80; 124.69; 124.55; 123.65, $J_{C\text{-}F}$ = 14.6; 119.97; 115.55, $J_{C\text{-}F}$ = 21.5; 67.73; 57.11; 47.29; 45.57. HRMS (ESI) m/z: $[M+H]^+$ Calc. for $C_{24}H_{21}FNO_3^+$ 390.1500, Found 390.1526.

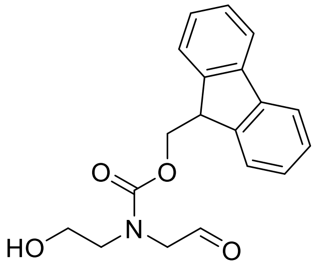

(9H-fluoren-9-yl)methyl (2-hydroxyethyl)(2-oxoethyl)carbamate: Diethanolamine (1.10 g, 0.010 mol) was dissolved in 30 ml of a 1:1 mixture of 1,4-dioxane and water. Then, $NaHCO_3$ (1.26 g, 0.015 mol) was added and the solution was cooled to 0°C. Next, Fmoc-OSu (3.37 g, 0.010 mol) was added to the mixture while maintaining the cooling. After 30 minutes, the cooling was removed, and the reaction mixture was stirred at room temperature for 17 hours. 1,4-Dioxane was evaporated using a rotary evaporator, and the product was extracted with ethyl acetate (2 x 80 ml). The extract was washed with saturated $NaHCO_3$ solution (40 ml), 0.5% HCl solution (40 ml) and water (2 x 40 ml). The organic layer was dried over anhydrous sodium sulfate, and the solvent was removed using a rotary evaporator. The crude product was dissolved in 20 ml of methylene chloride, and Dess-Martin periodinane (4.24 g, 0.010 mol) was added. The reaction mixture is stirred at room temperature for 1 hour. Then, 20 ml of chloroform and a pre-prepared solution of sodium thiosulfate (7.91 g, 0.050 mol) in 20 ml of saturated $NaHCO_3$ solution were added and stirred vigorously for 10 minutes. The product was extracted with chloroform (2 x 60 ml), washed with water (3 x 30 ml), and the organic layer is dried over anhydrous sodium sulfate. The solvent is removed using a rotary evaporator. The product was purified by column chromatography on silica gel using petroleum ether: ethyl acetate system (3:1 → 1.5:1). The pure fractions were combined and evaporated, and the product was dried in a vacuum desiccator for 24 hours. Yield: 3.25 g (81%), a colorless oil. NMR ^{1}H, (400 MHz, $CDCl_3$), δ, ppm. (*J*, Hz): 9.51 (1H, br s); 7.79-7.70 (2H, m); 7.61-7.50 (2H, m); 7.43-7.33 (2H, m); 7.32-7.25 (2H, m); 4.84 (1H, br s); 4.52-4.32 (2H, m); 4.26-4.17 (1H, m); 3.86-3.60 (2H, m), 3.56-3.37 (2H, m), 3.37-3.14 (2H, m). NMR ^{13}C (100 MHz, $CDCl_3$), δ, ppm.: 194.81; 155.43; 143.75; 141.25; 127.68; 127.01; 124.88; 119.94; 67.54; 61.55; 48.19; 47.20; 42.99. HRMS (ESI) m/z: $[M+H]^+$ Calc. for $C_{19}H_{20}NO_4^+$ 326.1387, Found 326.1369.
